# Dorsal Raphe VIP Neurons Are Critical for Survival‐Oriented Vigilance

**DOI:** 10.1002/advs.202523809

**Published:** 2026-01-25

**Authors:** Adriane Guillaumin, Emma Perrot, Thibault Dhellemmes, Laura Boi, Daniel De Castro Medeiros, Christelle Glangetas, Sylvie Dumas, Sandra Dovero, Nathalie Biendon, Elodie Ladeveze, Maëlle Hardel, Marc Landry, Erwan Bezard, Jérôme Baufreton, Gilberto Fisone, François Georges

**Affiliations:** ^1^ Univ. Bordeaux, CNRS, IMN, UMR 5293 Bordeaux France; ^2^ Department of Neuroscience Karolinska Institutet Stockholm Sweden; ^3^ ORAMACELL Paris France

**Keywords:** amygdala, anxiety, bed nucleus of the stria terminalis, central extended amygdala, defensive behavior, dopaminergic neurons heterogeneity, escape, NREM sleep, risk assessment, vasoactive intestinal peptide

## Abstract

Defensive behaviors are essential for survival, relying on risk assessment to detect and respond to threats. The dorsal raphe nucleus (DRN), a brain region involved in sleep‐wake regulation, contains dopaminergic neurons (DRN_DA_) with unclear roles in threat evaluation. A subset of these neurons expresses vasoactive‐intestinal‐peptide (VIP) and projects to two key regions for adaptive threat responses: the central amygdala (CeA) and the oval nucleus of the bed nucleus of the stria terminalis (ovBNST). We hypothesized that DRN_VIP_ neurons modulate sleep‐wake regulation and play a pivotal role in coordinating activity between the CeA and ovBNST, thereby influencing risk assessment and defensive response. We found that DRN_VIP_ neurons form a distinct feedback loop with PKC‐δ neurons in the CeA and ovBNST. These DRN_VIP_ neurons release glutamate and modulate excitability in target regions. Selective ablation of DRN_VIP_ neurons disrupts active‐phase sleep architecture, enhances risk assessment behaviors that may reflect dysfunctional processing, and impairs defensive responses. Fiber‐photometry revealed direct activation of the DRN_VIP_ neurons during presentation of the visual threat‐predictive cue in behaving mice. These results suggest that DRN_VIP_ neurons regulate specific sleep phases and control defensive behaviors, acting as a neuronal alarm system that responds to threats.

## Graphical abstract

DRN_VIP_ neurons in mice and primates are strategically positioned to influence the central extended amygdala via feedback loops. They regulate the excitability of PKC‐δ neurons in the ovBNST and CeA through glutamate release. Their ablation heightens activity in these regions, disrupts active‐phase sleep architecture, enhances risk assessment behaviors that may reflect dysfunctional processing, and alters defensive responses.

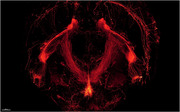

## 1 Introduction

Defensive behaviors are crucial for an organism's survival in the face of threats. In response to perceived threats, mammals – and mice in particular – display a range of defensive behaviors, including freezing, fleeing, hiding, and defensive aggression [1]. In a hostile or unfamiliar environment, risk assessment plays a central role in this repertoire of defensive responses [2]. Characterized by cautious exploration and information gathering, this behavior allows the individual to evaluate potential threats and determine the appropriate response. Research across species demonstrates that human defensive behaviors in response to threats closely mirror those observed in rodent models, with both exhibiting similar patterns of freezing, flight, and risk assessment that are modulated by factors such as threat ambiguity and escape opportunity [3, 4]. These defensive states involve heightened arousal, orchestrated by neural circuits that regulate sleep architecture supporting wakefulness during threat responses [5]. The presence of such shared mechanisms suggests that prolonged defensive states – such as anxiety‐driven risk assessment – may disrupt sleep homeostasis. This is supported by studies in rodents and non‐human primates showing that threat exposure alters sleep patterns, as well as human research linking rumination, a form of persistent non‐functional risk assessment, to sleep fragmentation [3, 5, 6].

The dorsal raphe nucleus (DRN), located within the ventromedial periaqueductal gray, is a major source of neuromodulators in the central nervous system [7] and plays a key role in regulating sleep homeostasis and diverse defensive behaviors, including aggression, escape responses, and possibly panic‐like reactions [8, 9, 10]. It contains molecularly distinct neuronal subtypes, which are classified into four main groups: serotonergic (5‐HT) neurons, followed by dopaminergic (DA), GABAergic, and glutamatergic neurons [11]. Midbrain dopaminergic neurons are crucial for encoding responses to unexpected sensory events and processing aversive experiences [12, 13, 14], however, only a few studies have explored this question for dopaminergic neurons outside the ventral tegmental area (VTA) [15], and none have specifically addressed their role in risk assessment and defensive behavior.

Significant progress in understanding the role of DA neurons in the brain has been made with the recognition of their heterogeneity [16]. This strategy over the past years has revealed that DA neurons are not a uniform population, but instead consist of subtypes with specialized roles, leading to a more nuanced comprehension of their contributions to neural circuits and behavior [14]. Over the past decades, research efforts have extensively focused on understanding the diversity of dopaminergic neurons in the VTA [17], somewhat less so for those in the substantia nigra pars compacta (SNc) [18], and has only recently begun for dopaminergic neurons in the DRN (DRN_DA_ neurons) [11, 19].

Electrophysiological, molecular, and RNA sequencing approaches have revealed substantial heterogeneity within the DRN_DA_ neurons, encompassing diverse electrophysiological properties, gene expression profiles, as well as distinct morphological and spatial characteristics. However, recent studies exploring the function of DRN_DA_ neurons have largely overlooked their cellular heterogeneity, yet have revealed a surprisingly diverse range of roles for these neurons. These include roles in modulating social behavior [20], mediating analgesia [21], encoding aversive teaching signals [15], controlling expression of incentive memory [22], promoting wakefulness [23, 24], and regulating both positive and negative motivational salience [23, 25], and more recently producing depressive phenotypes [26]. This diversity suggests an equally varied DA neurocircuitry within the DRN. Moreover, these investigations primarily relied on two transgenic mouse lines used to specifically target and manipulate dopaminergic neurons that expressed Cre under control of dopamine transporter (DAT) or tyrosine hydroxylase (TH) genes. Although these lines are highly specific for DA neurons in the VTA and SNc, both lines exhibit limited specificity for DRN_DA_, with off‐target effects on 30– 50% of non‐DA neurons [27].

Our primary goal in this study was to target a specific subpopulation of DRN_DA_ neurons using a transgenic mouse line with enhanced specificity. Recent studies have identified vasoactive intestinal peptide (VIP) mRNA, or VIP itself, as a molecular marker for a distinct subset of dopaminergic neurons within the DRN [19, 28, 29]. Using intersectional genetic labeling strategies, Poulin et al. demonstrated that VIP^+^ DA neurons located in the DRN (DRN_VIP_ neurons) project to the central amygdala (CeA) and the oval nucleus of the bed nucleus of the stria terminalis (ovBNST), thereby confirming that these cells are long‐range projection neurons – unlike cortical VIP^+^ neurons, which primarily function as local interneurons) [30]. Studies in rodents, non‐human primates, and humans have shown that the CeA and the ovBNST are key components of the central extended amygdala, essential for regulating adaptive responses to threats [31, 32]. Both structures share a common cellular lineage, a striatal‐like organization, and are predominantly composed of GABAergic neurons with similar structural and chemical features [33]. They also contain neurons expressing Protein Kinase C delta (PKC‐δ), a marker critical for emotional processing and the modulation of defensive behaviors based on the proximity of a threat [34, 35, 36]. Early work by Davis and colleagues suggests that the anatomical connection between the ovBNST and CeA is the basis of their functional interaction [37, 38]. This functional interaction becomes particularly significant as a threat approaches, prompting a shift from a hypervigilant, anxiety‐driven state governed by the BNST to a fear‐driven state mediated by the amygdala [32, 39]. However, rather than operating in a strictly sequential manner, recent findings suggest that the BNST and amygdala may be recruited simultaneously, indicating the involvement of a neural circuit capable of synchronizing the activity of both structures [40]. These findings led us to hypothesize that DRN_VIP_ neurons form a homogeneous population that, due to their anatomical projections to both the ovBNST and CeA, occupy a strategic position capable of coordinating the activity of these regions, thereby controlling risk assessment and defensive behavior. Sleep stability modulates neural and cognitive processes essential for evaluating threats and selecting adaptive defensive behaviors [41]. DRN_DA_ neurons have been characterized as a wake‐promoting system [23, 24, 42]. Optogenetic stimulation of these neurons rapidly awakens mice from sleep, while chemogenetic inhibition promotes non‐REM (NREM) sleep even in the presence of arousing stimuli, suggesting that DRN_DA_ neurons serve as a “gatekeeper” for wakefulness during environmentally relevant events [23]. Here, we investigate the function of DRN_VIP_ neurons in the context of sleep‐wake states and threat‐related behavior. In this context, we selected and validated the Vip‐Cre transgenic mouse line to target this specific subset of DRN_DA_ neurons projecting to the ovBNST and the CeA.

We used a combination of in situ hybridization and immunochemistry techniques in both mice and non‐human primate tissues, along with comprehensive whole‐brain mapping of cell type‐specific inputs and outputs. Additionally, we used in vivo electrophysiology, patch‐clamp methods for monitoring cell type‐specific circuits, and cell type‐specific genetic ablation to manipulate circuits. Our results show that, in both mice and primates, DRN_VIP_ neurons form an important subset of DRN_DA_ neurons. These neurons are strategically positioned to control the central extended amygdala through a feedback loop, receiving their main inputs from PKC‐δ neurons in the ovBNST and CeA. They also send axonal collaterals to both the ovBNST and CeA, where they regulate PKC‐δ neuron excitability via glutamate release. Selective genetic ablation of these neurons leads to increase in vivo electrophysiological activity in the BNST and CeA, and affects active‐phase sleep architecture and threat responses. Taken together, we propose that DRN_VIP_ neurons represent a neural subset that coordinates activity within the central extended amygdala, controlling sleep stability and modulating risk assessment and defensive behaviors.

## 2 Results

### 2.1 DRN_VIP_ Neurons Are a Subset of DRN_DA_ Neurons in Mice and Non‐Human Primates

**FIGURE 1:**
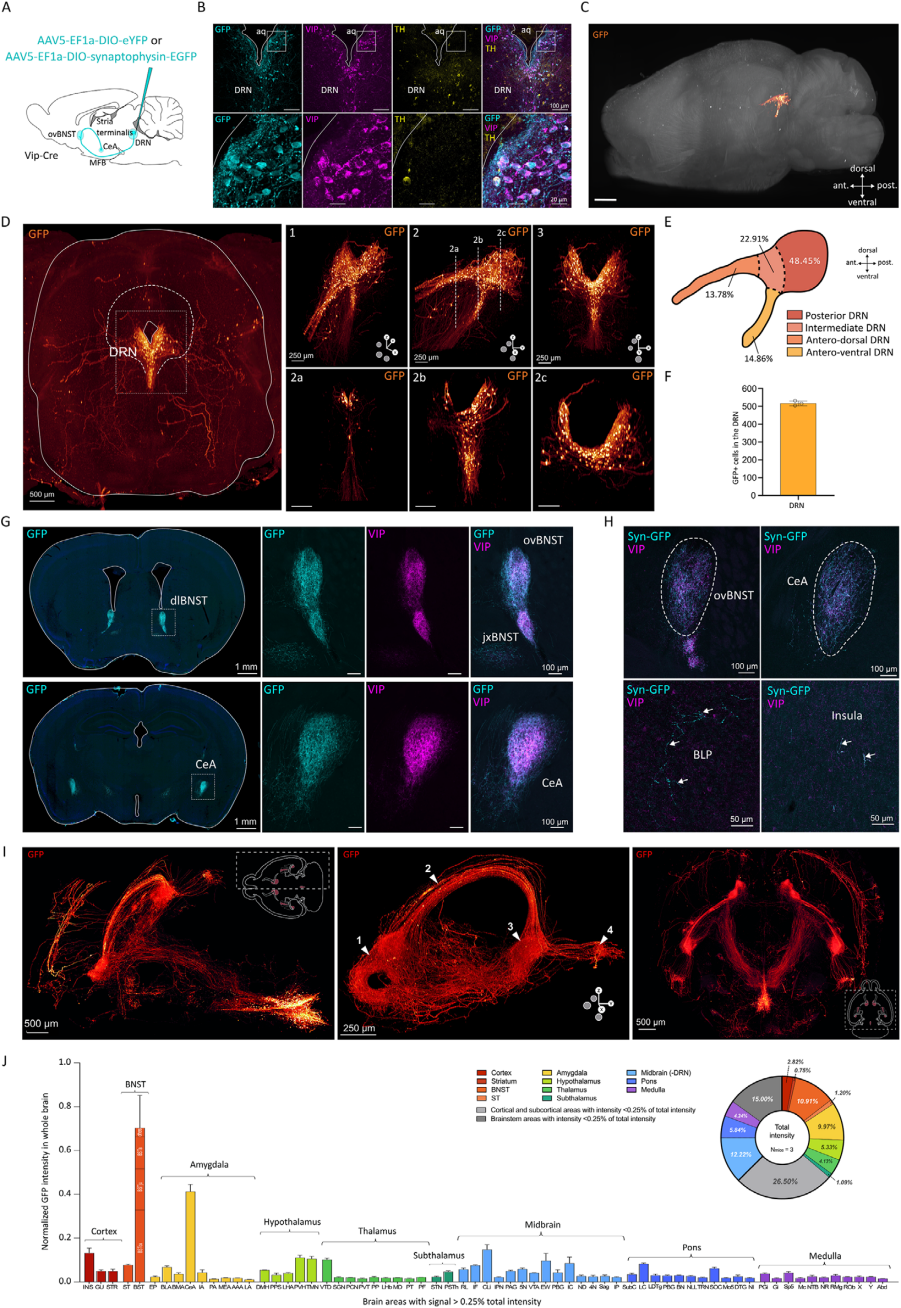
Anatomical characterization of DRN_VIP_ neurons and their projections. (A) Schematic representation of the injection site of the viral vector in Vip‐Cre mice for labeling DRN_VIP_ neurons. (B) Confocal images of DRN_VIP_ neurons showing colocalization of GFP protein with VIP and in few cases with TH. (C) Reconstructed image of the DRN of a Vip‐Cre mouse after viral transfection described in A and clearing technique. The image shows the location of DRN GFP positive cell bodies in the whole brain. (D) Left, whole DRN frontal slice showing that GFP positive neurons are located ventrally and laterally to the aqueduct of Sylvius and right, images showing the distribution pattern of DRN GFP positive neurons from different view angles: frontal slightly angled (1), sagittal (2), and frontal (3). Images 2a, 2b an 2c correspond to the three sections shown in image 2 at different antero‐posterior levels from most anterior (2a) to most posterior (2c). (E) Schematic representing the distribution of GFP neurons in the DRN from a sagittal perspective and subdivided into different subparts: antero‐dorsal, antero‐ventral, intermediate, and posterior. (F) Histogram showing the average number of transfected GFP positive cells in the DRN of Vip‐Cre mice (N = 3). (G) Epifluorescent images of GFP and VIP fibers in the dorsolateral BNST (dlBNST) and CeA from AAV5‐EF1a‐DIO‐eYFP injection. (H) Confocal images of synaptophysin‐EGFP expressed specifically in DRN_VIP_ neuron terminals in ovBNST, CeA, insula, and BLP with VIP immunostaining. (I) Images of Adipoclear technique showing DRN_VIP_ cell bodies and projections through MFB and stria terminalis to BNST, CeA, BLP, and insula from three different view angles, from left to right: a horizontal view of the right hemisphere, a sagittal view of the right hemisphere with white arrows pointing at the BNST (1), the stria terminalis (2), the CeA (3), and the BLP (4), and a horizontal view of both hemispheres. (J) Histogram showing a whole brain quantification (N = 3) of GFP fiber intensities per mm^3^ with a threshold superior to 0.25% of total intensity associated with a donut representation including all brain areas without intensity threshold. Abbreviations: MFB: medial forebrain bundle; CeA: central nucleus of the amygdala; ovBNST: oval nucleus of the bed nucleus of the stria terminalis; jxBNST: juxtacapsular nucleus of the BNST; Syn‐GFP: synaptophysin‐GFP; BLP: posterior basolateral amygdala; VIP: vasoactive intestinal peptide. DRN: dorsal raphe nucleus; DA: dopaminergic; aq: aqueduct of Sylvius; TH: tyrosine hydroxylase. Allen Brain Atlas abbreviations were used for naming brain structures.

Here, we focused our study on the dopaminergic VIP‐expressing neuronal population of the DRN. Using in situ hybridization, we demonstrated that approximately 50% of Th+ mRNA cells in the DRN also express Vip mRNA. Among these Vip+ neurons, the majority (83%) co‐express Th+ in C57BL/6 mice (Supplementary Figure 1A). To further validate this approach, we employed the Vip‐Cre mouse line to specifically target the DRN_VIP_ subpopulation, confirming their dopaminergic identity: 91% of the targeted cells expressed Th, and all (100%) co‐localized with Cre recombinase (Supplementary Figure 1B). Interestingly, while in situ hybridization identified this dopaminergic phenotype, TH immunostaining of VIP‐expressing neurons revealed a lower‐than‐expected expression of the TH enzyme (Figure 1A,B). This discrepancy suggests that VIP neurons exhibit low levels of TH protein, consistent with observations in other dopaminergic populations (Figure 1B; [43]). Using Cre‐dependent viral approaches in Vip‐cre mice (Figure 1A) combined with the AdipoClear tissue‐clearing technique, we selectively targeted DRN_VIP_ neurons and characterized the anatomical distribution of these neurons (Figure 1C–E). The overall organization of the DRN_VIP_ neuronal population resembles a “crab claw” with dense posterior and intermediate regions, accompanied by two extensions: a dorsal branch and a ventral branch, both oriented rostrally (Figure 1C–E). These DRN_VIP_ neurons are distributed along the lateral and ventral edges of the aqueduct of Sylvius, spanning the anteroposterior axis from −3.80 to −5.00 mm relative to bregma (Figure 1C–E). Quantification of GFP+ cell bodies in the DRN, performed on cleared brain samples (N = 3) and analyzed with Imaris software, revealed an average of 516 GFP+ neurons per brain (Figure 1F).

To investigate the existence of the DRN_VIP_ population in other species, we performed VIP immunostaining on non‐human primate coronal brain slices. DRN_VIP_ neurons of non‐human primates exhibit a distribution similar to that observed in mice, aligning along the lateral and ventral regions of the aqueduct of Sylvius (Supplementary Figure 2A). Notably, double immunohistochemistry for TH and VIP in the DRN showed a high degree of co‐localization between these markers, contrasting with the limited overlap observed in mice (Supplementary Figure 2B).

### 2.2 DRN_VIP_ Neurons Are Strategically Positioned to Influence the Central Extended Amygdala via a Feedback Loop

DRN_DA_ neurons project to multiple regions, including the BNST, CeA, BLA, VTA, and hypothalamus [20, 27]. Using AAV‐EF1a‐DIO‐eYFP injections in the DRN (Figure 1A) combined with the AdipoClear tissue‐clearing technique, we observed a highly restricted projection pattern for DRN_VIP_ neurons, with two primary output targets: the dorsolateral bed nucleus of the stria terminalis (dlBNST), including the oval (ovBNST) and juxtacapsular (juBNST) nuclei, and the central amygdala (CeA), particularly its lateral part (Figure 1G). Using an AAV5‐EF1a‐DIO‐synaptophysin‐GFP virus injected into the DRN of Vip‐Cre mice (Figure 1A,H), we confirmed that the observed labeling in the BNST, CeA, insular cortex, and posterior basolateral nucleus of the amygdala (BLP) corresponded to synaptic labeling rather than passing fibers. VIP immunostaining in NHPs revealed prominent VIP+ fibers in the ovBNST and CeA (Supplementary Figure 2C‐F). In cleared mice brains (Figure 1, Supplementary Figure 3), GFP+ fibers left the DRN ventrally, joined the MFB, and reached the BNST, where synaptic contacts were observed (Figure 1I). These fibers then traveled dorsally through the stria terminalis to innervate the CeA and, more caudally, the BLP. AdipoClear imaging also identified fibers diverging from the MFB before reaching the BNST, traveling directly to the CeA via the internal capsule. Secondary output targets included the BLP and insular cortex (Figure 1I). Quantitative fluorescence analysis confirmed strong projections to the dlBNST and anterior BNST, with the lateral CeA showing the highest fluorescence intensity among amygdala nuclei (Figure 1J). These results indicate that DRN_VIP_ neurons have a more restricted projection pattern compared to the entire DRN_DA_, with specific regions like the BLA excluded as targets. Images obtained from cleared brain samples strongly suggest that axons of DRN_VIP_ neurons sequentially innervate the BNST and then the CeA (Figure 1I). To confirm that individual DRN_VIP_ neurons can simultaneously project to both the BNST and CeA, we employed a modified rabies virus tracing strategy [44]. Using a Cre‐dependent helper virus lacking the glycoprotein (AAV1‐EF1a‐DIO‐TVA950‐T2A‐WPRE) along with two rabies viruses injected into the ovBNST (pSADB19dG‐GFP) and the CeA (PSADB19dG‐mCherry) of Vip‐Cre mice, we observed double‐labeled cell bodies in the DRN (Supplementary Figure 4). This finding confirms that DRN_VIP_ neurons project to both the BNST and CeA (Supplementary Figure 4B).

### 2.3 ovBNST_PKC‐δ_ and CeA_PKC‐δ_ Are the Main Synaptic Inputs to DRN_VIP_ Neurons

**FIGURE 2:**
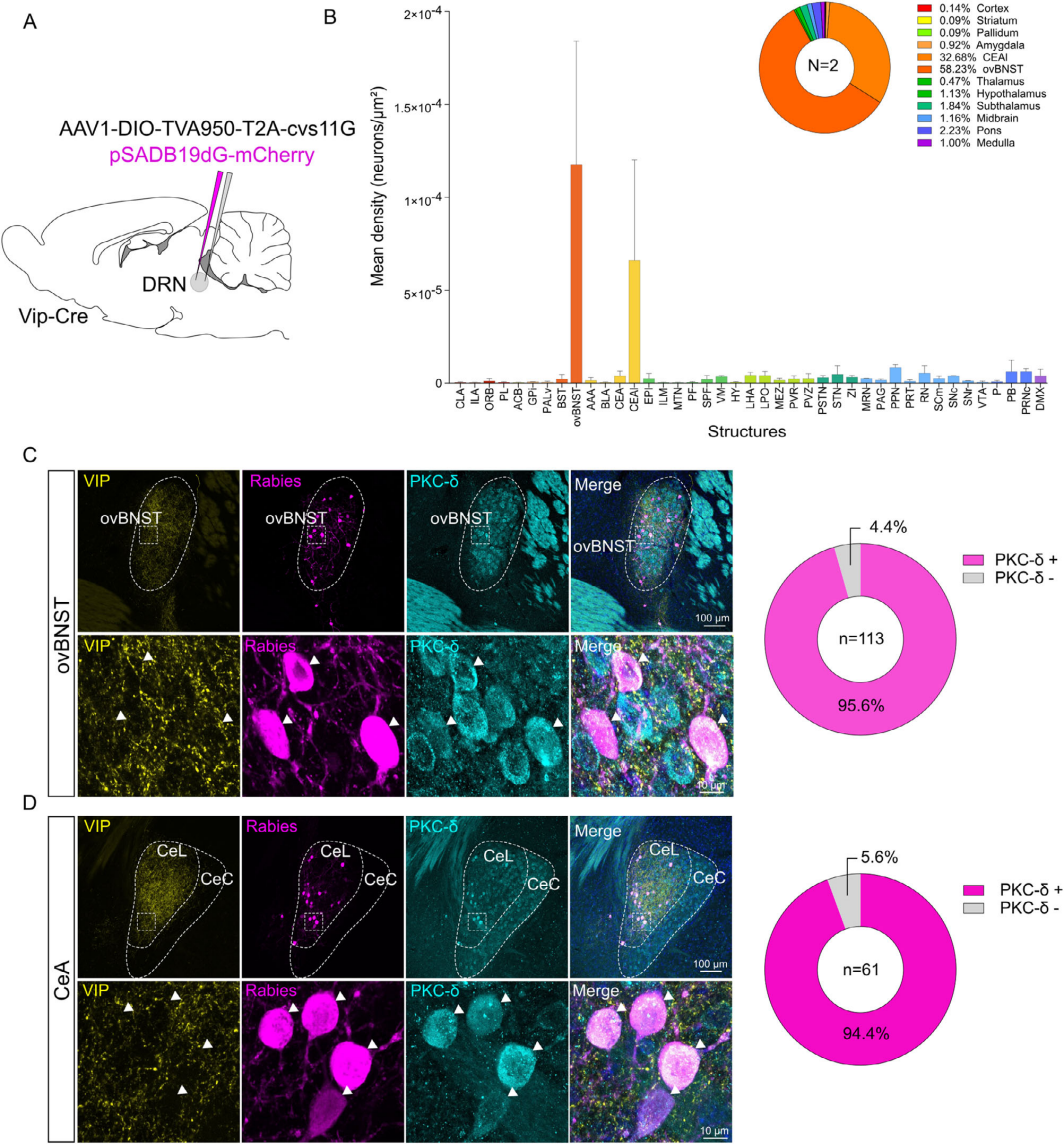
Characterization of DRN_VIP_ inputs. (A) Schematic representation of viral strategy to map inputs to DRN_VIP_ neurons using a Cre‐dependent helper virus (AAV1‐EF1a‐DIO‐TVA950‐T2A‐cvs11G) and a rabies virus (pSADB19dG‐mCherry) in Vip‐Cre mice. (B) Whole brain quantification of DRN_VIP_ neurons inputs from rabies viral strategy. Only structures with a mean density superior to 5.10^−7^ are shown in the histogram. The pie chart shows mean densities of main brain regions. (C) Confocal images and donut graphs showing co‐localization of the majority ovBNST and (D) CeA rabies positive neurons with PKC‐δ protein (N = 2 mice). Allen Brain Atlas abbreviations were used for naming brain structures.

To investigate the circuit in which DRN_VIP_ neurons are embedded, we employed a rabies‐virus‐based transsynaptic tracing approach in Vip‐Cre mice to map their presynaptic inputs (Figure 2A). First, we confirmed the presence of starter cells co‐expressing the helper virus and rabies reporter in the DRN (Supplementary Figure 5A,B). Surprisingly, our tracing experiment revealed that only two major brain structures provide substantial input to DRN_VIP_ neurons: the ovBNST and the lateral CeA (Figure 2B). While additional inputs arise from regions predominantly located in the hypothalamus and pons, these projections are comparatively sparse (Figure 2B). Previous studies have identified two main, non‐overlapping neuronal populations within the ovBNST and CeA: one expressing protein kinase C delta (PKC‐δ), which co‐expresses D2 dopamine receptors, and another expressing corticotropin‐releasing hormone (CRH) [26, 33]. Since PKC‐δ neurons constitute the largest subpopulation in both the ovBNST and CeA, we performed PKC‐δ immunohistochemistry to characterize the identity of neurons projecting to DRN_VIP_ neurons. Our analysis revealed a strikingly high degree of colocalization between rabies‐labeled neurons and PKC‐δ expression, with 95.6% in the ovBNST and 95.4% in the CeA (Figure 2C,D). These findings reveal a highly specialized circuit in which DRN_VIP_ neurons receive dense input from two GABAergic structures, the ovBNST and CeA, while simultaneously projecting back to these same regions, forming a restricted feedback loop.

### 2.4 DRN_VIP_ Neurons Release Glutamate

**FIGURE 3:**
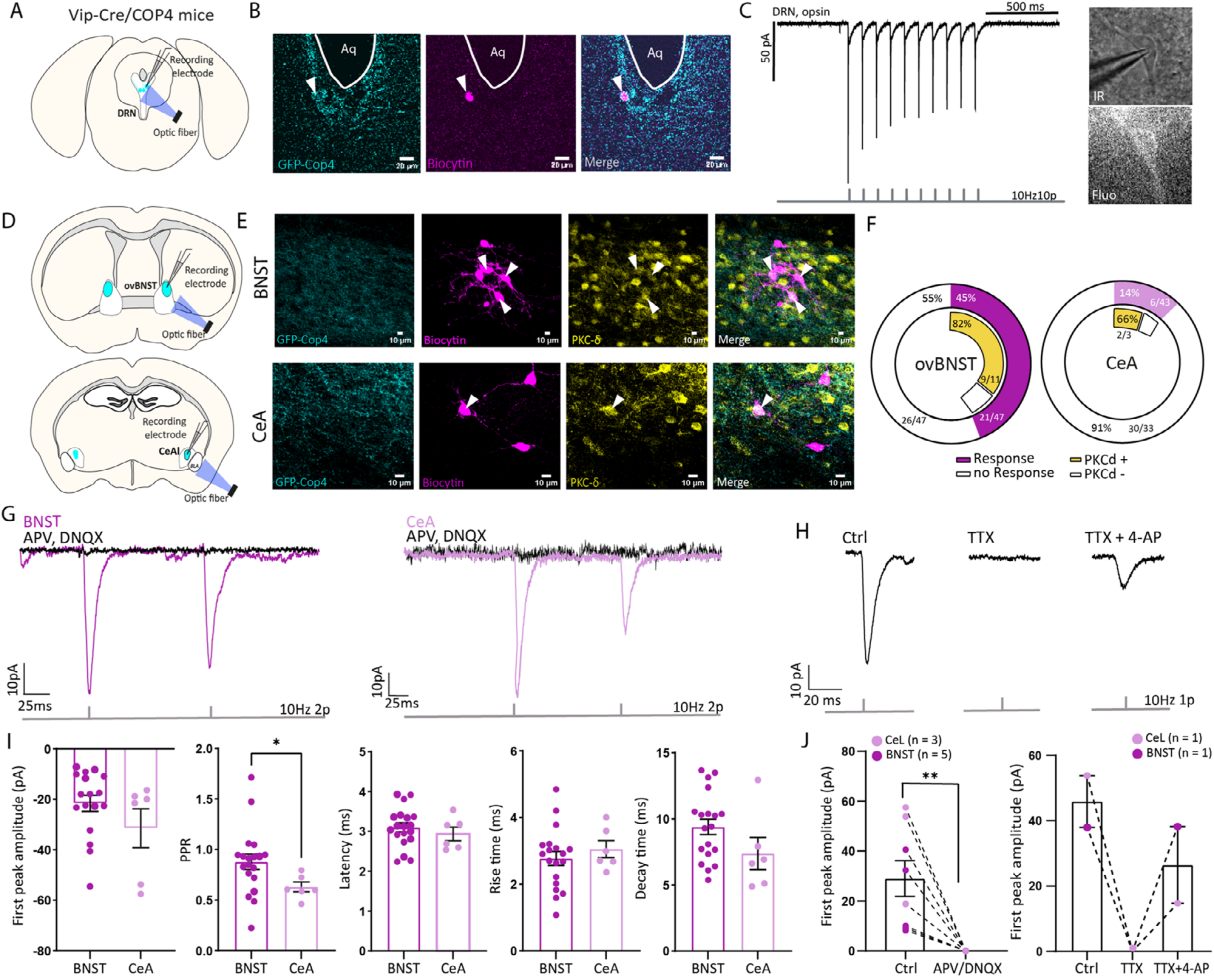
*Ex vivo* characterization of DRN_VIP_ synaptic transmission to ovBNST and CeA. (A) Schematics of a DRN slice and positioning of recording electrode and optic fiber in Vip‐Cre/COP4 mice. (B) Confocal images of a patched GFP+ neuron filled with biocytin in the DRN of a Vip‐Cre/COP4 mouse (C). Channelrhodopsin evoked currents in a DRN GFP+ neuron associated with fluorescent and infrared images (D). Schematics of ovBNST and CeA slices and positioning of recording electrode and optic fiber in Vip‐Cre/COP4 mice. (E) Confocal images of patched neurons filled with biocytin and PKC‐δ immunostaining in ovBNST and CeA. (F) Donut graphs showing the proportion of neurons responding to optogenetic stimulation of DRN_VIP_ fibers colocalizing with PKC‐δ. (G) Excitatory post‐synaptic evoked currents recorded in ovBNST and CeA neurons upon optogenetic stimulation of DRN_VIP_ fibers. Purple curves show inward currents abolished by application NMDA/AMPA antagonists, APV and DNQX (black curve). (H) Example of an excitatory post‐synaptic evoked current recorded in the CeL upon optogenetic stimulation of DRN_VIP_ fibers in a control condition, with TTX that abolished the inward current and with 4‐AP that rescued the signal to show the monosynaptic connection. (I) *Ex vivo* electrophysiological properties of ovBNST and CeA excitatory post‐synaptic currents evoked by optogenetic stimulation of DRN_VIP_ fibers (first peak amplitude (pA), Paired‐pulse ratio (PPR), Rise time (ms), and Decay time (ms)). (J) Addition of drugs in the bath shows, first, the glutamatergic nature (APV and DNQX) of the signal and second, the monosynaptic connection (TTX and 4‐AP) between DRN_VIP_ and ovBNST or CeL. Abbreviations: DRN: dorsal raphe nucleus; ovBNST oval nucleus of the bed nucleus of the stria terminalis; Aq: aqueduct of Sylvius; CeA: central nucleus of the amygdala; PPR: paired‐pulse ratio.

DRN_DA_ neurons have been shown to co‐release dopamine and glutamate in downstream structures [20]. Here, we investigated whether DRN_VIP_ neurons also release glutamate. To address this, we used *Vip‐Cre/COP4* mice, in which channelrhodopsin is selectively expressed in VIP‐expressing neurons. Brain slices containing the DRN, ovBNST, and CeA were prepared, and a blue‐emitting optical fiber was placed either over DRN cell bodies to confirm depolarization upon light stimulation (Figure 3A) or over synaptic terminals in the ovBNST and CeA to assess neurotransmitter release (Figure 3D). We validated channelrhodopsin expression in DRN_VIP_ neurons by recording evoked inward currents upon light stimulation (Figure 3B,C). Optogenetic stimulation revealed that 45% of recorded neurons in the ovBNST (n = 21/47) responded, compared to only 14% in the CeA (n = 6/43) (Figure 3F). All responsive neurons exhibited excitatory post‐synaptic currents (EPSCs), indicating a glutamatergic phenotype (Figure 3G). Furthermore, the addition of AMPA and NMDA receptor antagonists (DNQX and AP5) abolished these currents, confirming that DRN_VIP_ neurons release glutamate in both structures (Figure 3G). Moreover, these connections appear to be monosynaptic because the addition of TTX (a voltage‐gated Na+ blocker) leads to a loss of signal, which is then recovered after the addition of 4‐AP (a K+ channel blocker), both in the ovBNST and the CeL (Figure 3H,J). Characterization of synaptic transmission revealed low EPSC amplitudes and synaptic depression in both ovBNST and CeA neurons (Figure 3I). Given that a major neuronal population in these regions expresses PKC‐δ, we examined whether DRN_VIP_ neurons preferentially target PKC‐δ‐expressing cells. Immunostaining for PKC‐δ showed high colocalization with biocytin‐filled neurons, reaching 82% (n = 9/11) in the ovBNST and 66% (n = 2/3) in the CeA, suggesting that DRN_VIP_ neurons predominantly innervate PKC‐δ‐expressing cells.

### 2.5 DRN_VIP_ Neuron Ablation Leads to Sleep Stability

**FIGURE 4:**
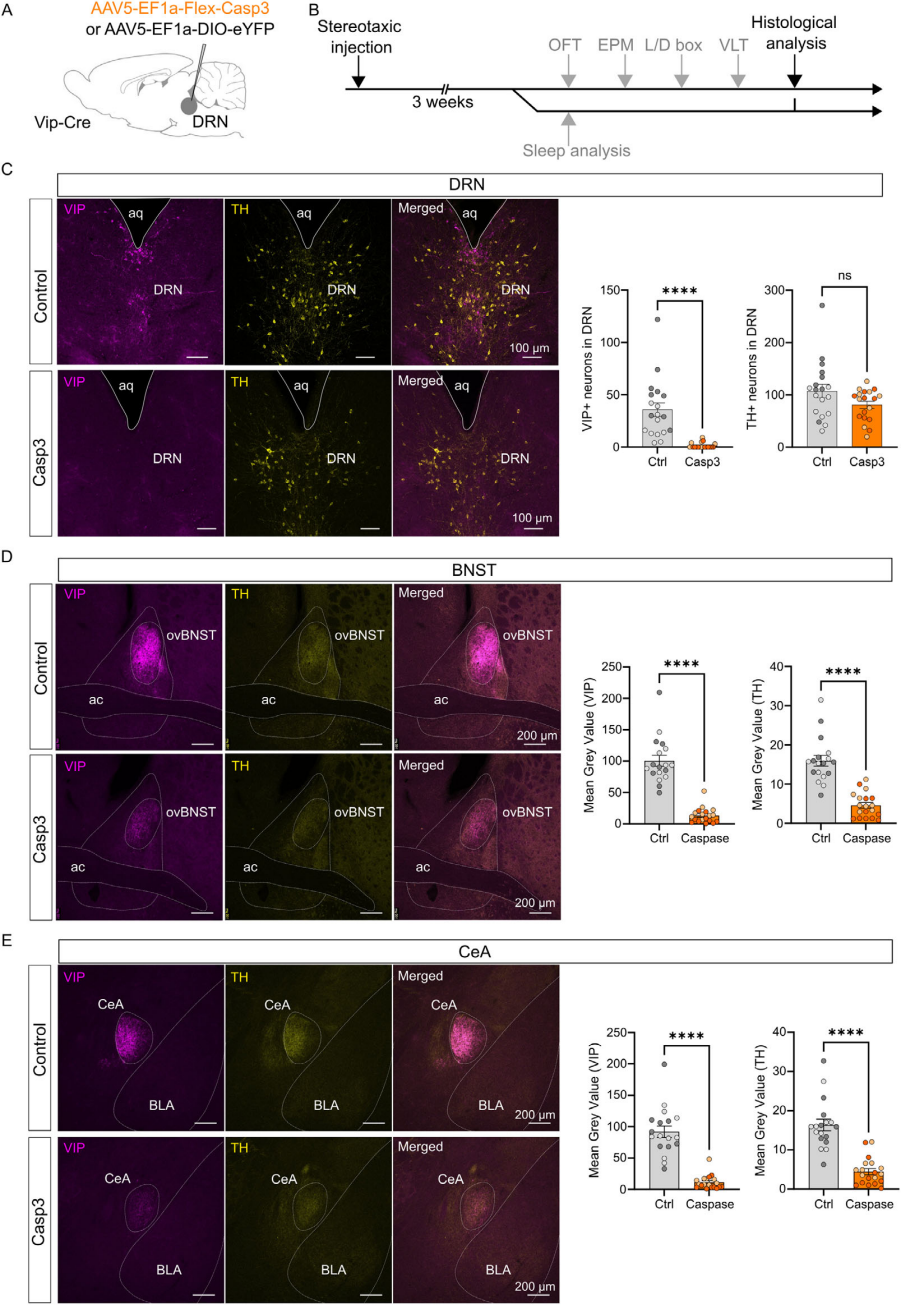
Histological analysis of Casp3 behavioral experimental group. (A) Schematic representation of AAV5‐EF1‐Flex‐Casp3 injection in the DRN of Vip‐Cre mice and control virus. (B) Chronological timeline of the experimental procedure from virus injection to histological analysis. Mice were separated into two batches, one for assessing anxiety‐related behaviors and defensive behaviors, and one for sleep analysis. (C) Confocal images and quantification of TH+ and VIP+ neurons in the DRN of AAV5‐EF1‐Flex‐Casp3 injected mice (referred as Casp3) and AAV5‐EF1a‐DIO‐eYFP injected mice (referred as Ctrl) (Mann–Whitney test, p‐value<0.0001 for VIP+ quantification; Unpaired T‐test, p‐value = 0.0753 for TH+ quantification). (D) Confocal images and quantification of mean intensity of TH+ and VIP+ fibers in the ovBNST of Casp3 mice and controls (Mann–Whitney test, p‐value<0.0001 for both VIP+ and TH+ quantification). (E) Confocal images and quantification of mean intensity of TH+ and VIP+ fibers in the CeA of Casp3 mice and controls (Mann–Whitney test, p‐value < 0.0001 for both VIP+ and TH+ quantification). In the graphs, light circles represent female mice and dark circles represent male mice. Graphs show mean ± SEM. Abbreviations: OFT: open field test; EPM: elevated plus maze; L/D box: light/dark box; VLT: visual looming test; DRN: dorsal raphe nucleus; ovBNST: oval nucleus of the bed nucleus of the stria terminalis; CeA: central nucleus of the amygdala; BLA: basolateral nucleus of the amygdala; aq: aqueduct of Sylvius; ac: anterior commissure.

**FIGURE 5:**
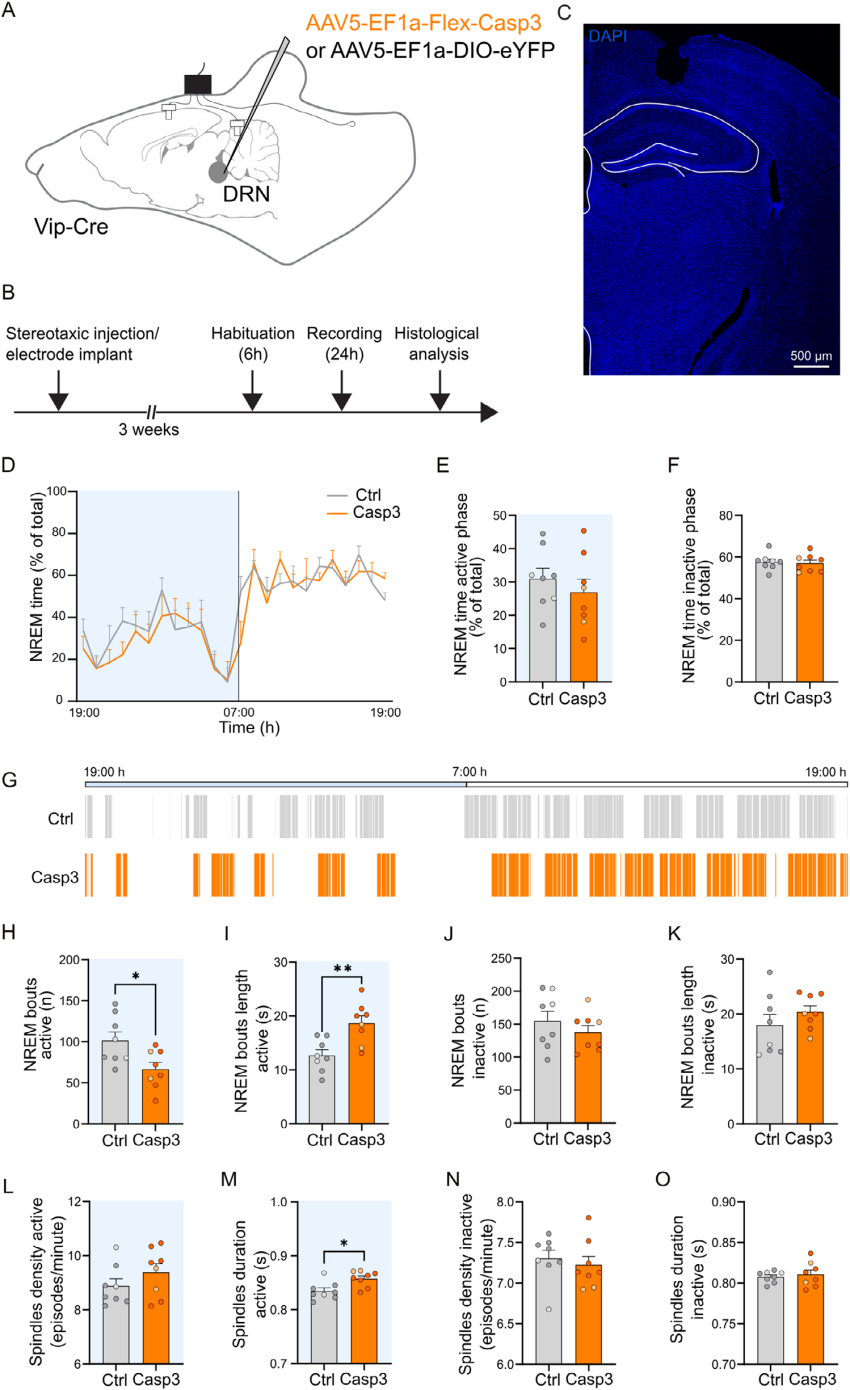
Effect of DRN_VIP_ genetic ablation on NREM state in Vip‐Cre mice. (A) Schematic representation showing AAV5‐EF1a‐Flex‐Casp3/AAV5‐EF1a‐DIO‐eYFP injections in the DRN of Vip‐Cre mice, and EEG, reference, and EMG placement. (B) Experimental timeline of sleep analysis. (C) Representative confocal picture showing cortical EEG positioning. (D) Graphics showing NREM sleep percent time during active (19‐ to 7 h) and inactive (7‐ to 19 h) phases in Ctrl and Casp3 groups. (E, F) Bar graphs showing NREM sleep percent during the active (19‐ to 7 h), (E, Unpaired T‐test p‐value = 0.4375) and the inactive (7‐ to 19 h), (F, Unpaired T‐test p‐value = 0.7605) phases in Ctrl and Casp3 groups. (G) Representative illustration of the NREM sleep bouts distribution in Ctrl and Casp3 mice during the 24‐hour circadian cycle. Bars indicate NREM sleep episode occurrences. (H, I) Bar graphs showing the number (H, Unpaired T‐test p‐value = 0.0207) and the length (I, Unpaired T‐test p‐value = 0.0038) of NREM sleep bouts during the active phase (19‐ to 7 h) in Ctrl and Casp3 groups. (J, K) Bar graphs showing the number (J, Unpaired T‐test p‐value = 0.3486) and the length (K, Unpaired T‐test p‐value = 0.3050) of NREM sleep bouts during the inactive phase (7‐ to 19 h) in Ctrl and Casp3 groups. (L, M) Bar graphs showing NREM sleep spindles density (L, Unpaired T‐test p‐value = 0.2357) and duration (M, Unpaired T‐test p‐value = 0.0118) during the active phase (19‐ to 7 h) in Ctrl and Casp3 groups. (N, O) Bar graphs showing NREM sleep spindles density (N, Unpaired T‐test p‐value = 0.5951) and duration (O, Unpaired T‐test p‐value = 0.6303) during the inactive phase (7‐ to 19 h) in Ctrl and Casp3 groups. Light circles in bars correspond to female mice, while dark circles correspond to male mice. *p‐value < 0.5, **p < 0.01. Graphs show mean ± SEM.

Population activity of DRN_DA_ neurons is closely correlated with sleep‐wake states, and chemogenetic inhibition of these neurons reduces responsiveness to external stimuli, impairing the ability to awaken from sleep [23]. Moreover, chemical lesions targeting these cells result in the onset of severe hypersomnia [24]. Building on these findings, we investigated whether ablation of DRN_VIP_ neurons could disrupt sleep architecture by altering the balance between wakefulness and sleep states. Specifically, we hypothesized that modulating the activity of these neurons could impact the stability and duration of NREM episodes, ultimately influencing the overall sleep‐wake cycle. To determine whether DRN_VIP_ dopamine subgroup exerts distinct effects on sleep‐wake regulation [23], we selectively ablated DRN_VIP_ neurons using an AAV5‐EF1a‐FLEX‐taCaspase3 viral construct (Casp3), while control mice received an AAV5‐EF1‐DIO‐eYFP virus (Ctrl) in the DRN of Vip‐Cre mice (Figure 4A). Histological analysis, conducted after a series of behavioral tests (Figure 4B), confirmed that Casp3 ablation effectively eliminated VIP+ neurons in the DRN (Figure 4C). Additionally, VIP and TH fiber density was markedly reduced in the ovBNST and CeA (Figure 5D,E), further supporting the dopaminergic phenotype of DRN_VIP_ neurons despite their low TH expression in cell bodies. When comparing the number of VIP+ and TH+ neurons in female and male mice, we noticed a higher number of neurons in control male mice than in control female mice (Supplementary Figure 7A,B). No other differences were observed between female and male mice (Supplementary Figure 7).

Caspase‐induced ablation of DRN_VIP_ neurons resulted in significant alterations of sleep architecture occurring during the active phase of the 24‐hour recording period (Figure 5). Specifically, the number of NREM sleep episodes decreased, while their average duration increased (Figure 5H,I), resulting in enhanced sleep stability in comparison to control. This increase in stability occurred in concomitance with changes in NREM spindles [45, 46], which are brief 10–15 Hz oscillatory events implicated in the modulation of sensory inputs and spontaneous sleep disruptions [47]. We observed a significant increase in spindle duration (Figure 5M), along with evidence suggesting a contribution of spindle density to NREM sleep regulation (Figure 8A’). Importantly, total NREM sleep time remained unchanged (Figure 5J,K), excluding the occurrence of excessive daytime sleepiness (EDS). In line with the decreased number of NREM episodes, we observed a reduced number of bouts in the AWAKE state (Supplementary Figure 6D). No differences were observed in the duration of wakefulness or REM sleep (Supplementary Figure 6).

### 2.6 DRN_VIP_ Neuron Ablation Changes Risk Assessment in Anxiety‐Related Tests

**FIGURE 6:**
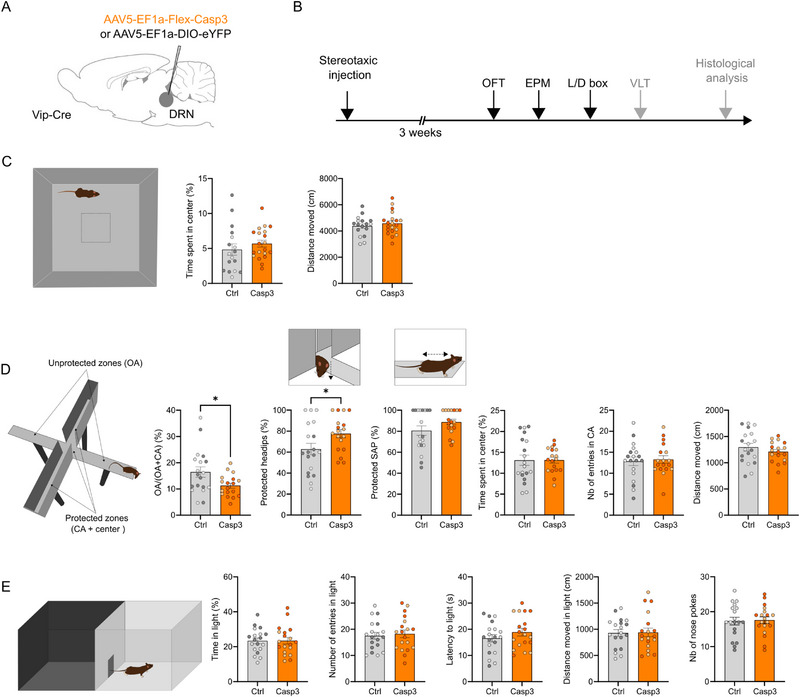
Effect of DRN_VIP_ genetic ablation on risk assessment, anxiety, and locomotion in Vip‐Cre mice. (A) Schematic representation of AAV5‐EF1a‐Flex‐Casp3 or control viruses injections in the DRN in Vip‐Cre mice. (B) Chronological timeline of the experimental procedure from virus injection to the three behavioral tests depicted below. (C) Schematic representation of the Open Field Test (OFT) arena. Quantification of the time in spent in the center (Unpaired T‐test, p‐value = 0.3591) and distance moved (Unpaired T‐test, p‐value = 0.4645) between control and Casp3 mice. (D) Schematic representation of the Elevated Plus Maze (EPM) arena with arrows indicating protected (closed arms CA and center) and unprotected (open arms OA) zones. Quantification of the time spent in OA expressed in ratio (Unpaired T‐test p‐value = 0.0267), protected headips (Unpaired T‐test p‐value = 0.0396), protected SAP (Mann–Whitney test, p‐value = 0.2841), time spent in center (Unpaired T‐test p‐value = 0.8845), number of entries in CA (Unpaired T‐test p‐value = 0.7502) and distance moved (Unpaired T‐test p‐value = 0.329) between control and Casp3 mice. (E) Schematic representation of the Light/Dark box (L/D box) arena. Quantification of the time spent in the light compartment (Unpaired T‐test p‐value = 0.9668), number of entries in light compartment (Unpaired T‐test p‐value = 0.7103), latency to light compartment (Unpaired T‐test p‐value = 0.2553), distance moved in light compartment (Unpaired T‐test p‐value = 0.9202), and number of nose pokes (Unpaired T‐test p‐value = 0.8581). Light circles in graphs correspond to female mice, while dark circles correspond to male mice. *p‐value < 0.5. Graphs show mean ± SEM.

Both human and rodent studies have demonstrated that the BNST and CeA play a key role in threat monitoring, including anxiety‐like behaviors, risk assessment, and defensive responses [15, 39, 40]. Given our finding that the ovBNST and CeA (lateral division) are the two main synaptic targets of DRN_VIP_ neurons, we employed the same genetic ablation approach (Figure 4) to investigate their role in anxiety, risk assessment, and defensive behaviors. To assess how the loss of these neurons affected BNST and CeA activity, we performed in vivo single‐cell electrophysiological recordings. Genetic ablation of DRN_VIP_ neurons led to a significant increase in neuronal firing frequency in both regions (Supplementary Figure 11). To examine the behavioral consequences of DRN_VIP_ neuron ablation, we assessed anxiety‐related and risk assessment behaviors using the open field test (OFT), elevated plus maze (EPM), and light/dark box (L/D box) (Figure 6A,B). No differences were observed between Casp3 and control mice in the OFT (Figure 6C). In the EPM, Casp3 mice spent significantly less time in the open arms (OA) and exhibited more protected head dips, indicating increased risk assessment behavior (Figure 6D) [48, 49]. However, no differences were found in other risk assessment or anxiety‐related measures, such as stretch‐attend postures (SAP), center time, closed‐arm entries, or total distance traveled (Figure 6D). In the L/D box test, Casp3 mice did not show reduced time in the lighted compartment, and none of the measured variables significantly differed from controls (Figure 6E). No differences were observed between females and males to the exception of the L/D box test in which a sex effect was observed for both control and Casp3 mice, probably related to the test in itself rather than to the ablation of DRN_VIP_ neurons (Supplementary Figure 8).

### 2.7 DRN_VIP_ Neurons are Required for Adaptive Escape Responses to Looming Threats

**FIGURE 7:**
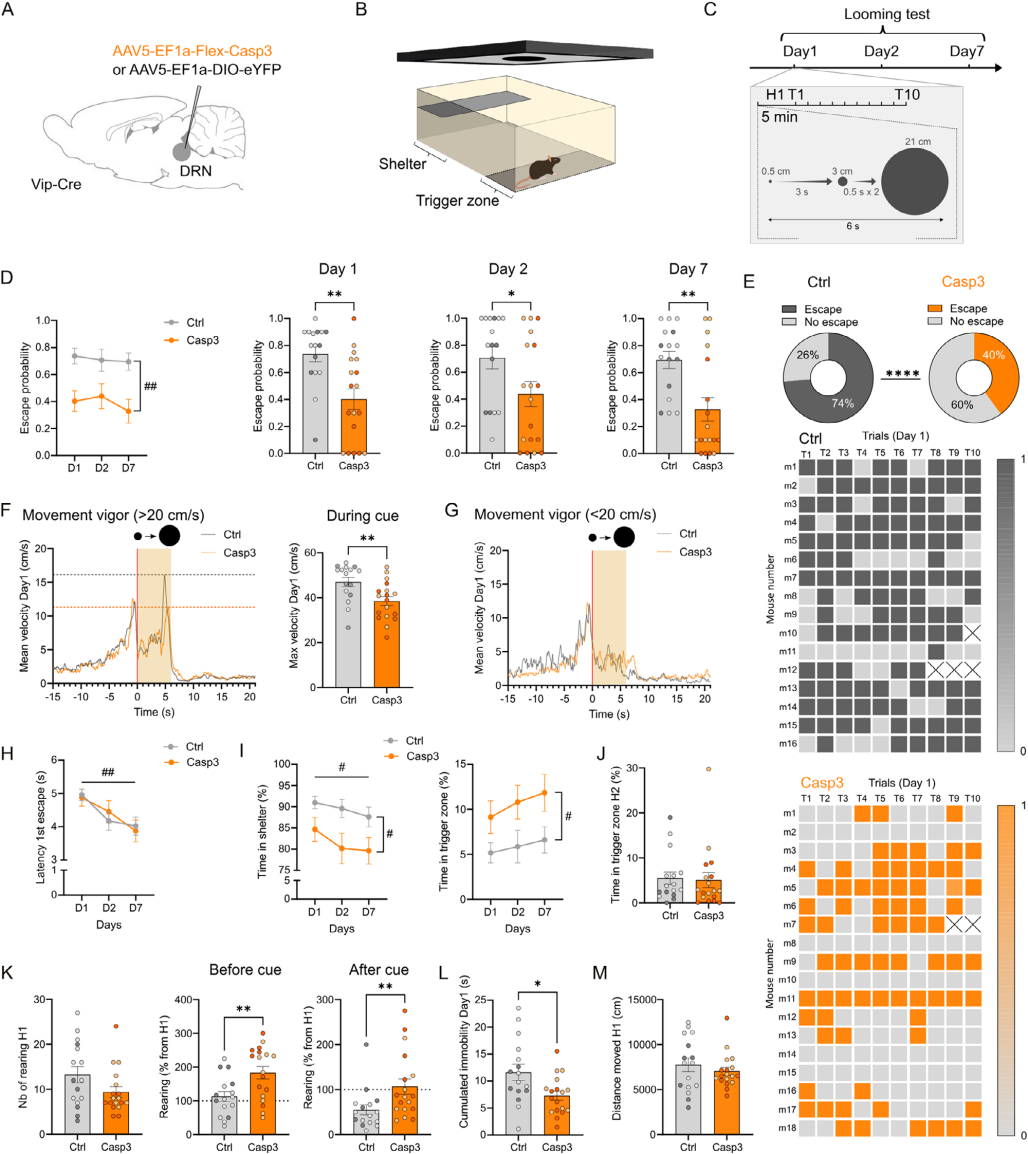
Effect of DRN_VIP_ genetic ablation on defensive behaviors in Vip‐Cre mice. (A) Schematic representation of AAV5‐EF1a‐Flex‐Casp3 and control virus injections in the DRN in Vip‐Cre mice. (B) Schematic representation of the visual looming test setup. (C) Experimental timeline of the visual looming test with a detailed view of the procedure for Day 1 showing 5 min of habituation followed by 10 cues (= 10 trials) represented by a black expanding round. (D) Quantification of the escape probability between control and Casp3 mice along days (Mixed‐effects analysis, F_group_ (1, 32) = 10.20, p‐value = 0.0031) and for each day (Mann–Whitney test, Day1 p‐value = 0.0013, Day2 p‐value = 0.0423, Day7 p‐value = 0.0034). (E) On the top, donut graphs showing the percentage of escaping and not escaping trials in control mice (left) and Casp3 mice (right) (Fisher's exact test, p‐value < 0.0001). Below are two heatmaps showing the detailed behavior at Day 1, escaping (dark color) or not escaping (light color) for each mouse and for each trial with control mice in the top heatmap and Casp3 mice in the bottom heatmap. Trials with a cross mean that the mouse did not perform the trial. (F) Graphs showing the average velocity of control and Casp3 mice at Day 1 for “escape” trials on the left and no escape trials on the right. The histogram on the right shows the max velocity at Day 1 for “escape” trials, representing the escape vigor (Mann–Whitney test, p‐value = 0.0056). In these three graphs only, the velocity threshold for “escape” trials was set on 20 cm/s to measure the vigor of the return to the shelter. No escape trials correspond to a velocity below 20 cm/s for the return to the shelter or the absence of return to the shelter. Light orange rectangles correspond to the period when the visual looming cue is presented. (G) Graph showing the latency of the first escape at Days 1, 2, and 7 (Mixed‐effects analysis, F_Days_ (1.928, 53,03) = 7.372, p‐value = 0.0017). (H) Graphs showing the total time in shelter (2Way RM ANOVA, F_Group_ (1, 32) = 5.343 p‐value = 0.0274, F_Days_ (1.892, 60,53) = 3.747 p‐value = 0.0314) and trigger zone (2Way RM ANOVA, F_Group_ (1, 32) = 4.616, p‐value = 0.0393) at Days 1, 2, and 7. (I) Graph showing the time in trigger zone during the 5 min habituation of Day 2 (H2) (Mann–Whitney test, p‐value = 0.4174). (J) Graphs showing quantification of rearing behavior during the 5 min habituation of Day 1 (H1) (Mann–Whitney test, p‐value = 0.4375) and as a percentage from H1 before and after the visual looming cue (Before cue: Unpaired T‐test, p‐value = 0.0062, One sample T‐test, p‐value = 0.3974 for controls and 0.0003 for Casp3; After cue: Mann–Whitney test, p‐value = 0.0046, One sample T‐test, p‐value = 0.0013 for controls and 0.6910 for Casp3). (K) Graph showing the cumulated immobility at Day 1 during the visual looming cues (Unpaired T‐test, p‐value = 0.0135). (L) Graph showing the distance moved during the 5 min habituation of Day 1 (H1) (Mann–Whitney test, p‐value = 0.6212). Light circles in graphs correspond to female mice, while dark circles correspond to male mice. Significant effects using 2Way RM ANOVA or Mixed‐effects analyses are represented with a hash symbol. Graphs show mean ± SEM. Significant effects using Unpaired t‐test or Mann–Whitney test are represented with an asterisk symbol. #*p*‐value < 0.05, ##*p*‐value < 0.01, *p‐value < 0.5, **p‐value < 0.01.

The BNST is critical for threat anticipation, particularly in response to uncertain threats [50, 51, 52, 53, 54]. To assess this function in Casp3 mice, we employed a visual looming test (VLT) (Figure 7A‐C) [55, 56]. In this task, a visual stimulus mimicking an approaching predator is triggered when the mouse enters a designated zone, prompting escape or other defensive behaviors (Figure 7B). The looming stimulus was specifically designed to favor escape responses over freezing. Casp3 mice exhibited a significantly lower probability of escape compared to controls across all three testing days (Figure 7D). Control mice displayed escape responses in 74% of trials, whereas Casp3 mice escaped in only 40% of trials (Figure 7E). Furthermore, escape vigor – quantified as the velocity of the return to the shelter – was reduced in Casp3 mice compared to controls. While control mice returned to the shelter with increased velocity, Casp3 mice moved at a similar speed during both the outward trip and the escape (Figure 7F). Escape latency at the first escape trial was similar between groups and decreased over repeated testing (Figure 7G). In addition to reduced escape probability, Casp3 mice spent less time in the shelter and more time in the trigger zone than control mice (Figure 7H). Since both control and Casp3 mice were exposed to the visual cue between 7 and 10 times on Day 1 of the VLT, we hypothesized that the VLT chamber had acquired an aversive valence by Day 2. However, no significant differences were observed between groups in the time spent in the trigger zone during the habituation phase on the second day (H2) of the VLT (Figure 7I). To further investigate risk assessment, we quantified rearing behavior, a relevant measure in the VLT since the visual stimulation mimics an overhead airborne predator. No differences in rearing behavior were observed between groups during habituation of day 1 (H1) (Figure 7J); however, Casp3 mice performed significantly more rearing both before and after visual cues, suggesting increased risk assessment (Figure 7J). To assess defensive responses, we measured cumulative immobility within 7s of stimulus onset across 10 trials. Casp3 mice spent significantly less time immobile than controls (Figure 7K). Importantly, no differences were observed in general locomotor activity between groups (Figure 7L).

We sought to determine whether long‐term molecular or post‐synaptic compensations, rather than the loss of DRN_VIP_ inputs themselves, accounted for the behavioral phenotype in Casp3 mice. To exclude this possibility, we repeated the visual looming test while transiently inhibiting DRN_VIP_ neurons optogenetically during each visual cue (Supplementary Figure 9A,C,D). Mice injected with the opsin eNpHR showed a lower escape probability at Day 1 compared to control mice. At Day 1, eNpHR mice escaped 14% of the time while control mice escaped 34% of the time (Supplementary Figure 9E,G). Other measured variables were not different between eNpHR mice and controls (Supplementary Figure 9H–L). Histological analysis confirmed mCherry expression in the DRN and the correct placement of the optic (Supplementary Figure 9B). For escape probability (Supplementary Figure 9E), statistical comparisons were performed using an unpaired one‐tailed t‐test. This approach was justified by a strong a priori, directional hypothesis predicting a decrease in escape probability following optogenetic inhibition of DRN_VIP_ neurons, based directly on our genetic ablation experiments (Figure 7). Statistical significance was not reached with a two‐tailed test, likely due to a lower baseline escape probability in the control group of the optogenetic experiment compared to that of the genetic ablation experiment. We attribute this difference to the presence of the optic fiber, which may influence baseline behavior. Nevertheless, optogenetic inhibition of DRN_VIP_ neurons still produced a significant reduction in escape responses relative to its matched control (Supplementary Figure 9G). These results collectively support the conclusion that the reduced escape probability observed in Casp3 mice is primarily attributable to the loss of DRN_VIP_ neuronal activity, rather than adaptive changes.

### 2.8 Chemogenetic Activation of DRN_VIP_ Neurons Increases Escape Responses to Looming Threats

Results from the caspase experiment showed a maladaptive response to a looming threat, evidenced by a decreased escape probability. We then hypothesized that increasing the excitability of DRN_VIP_ neurons using an excitatory chemogenetic approach would enhance escape probability in the VLT. We injected an AAV1‐EF1a‐DIO‐hM3D(Gq)‐mCherry (N = 14) or the control virus AAV1‐EF1a‐DIO‐mCherry (N = 19) in the DRN of Vip‐Cre mice (Supplementary Figure 10A). Histological analysis confirmed the colocalization of mCherry reporter with DRN_VIP_ neurons (Supplementary Figure 10C). Four weeks post‐surgeries, we first tested potential changes in anxiety, risk assessment, and locomotion parameters in a series of behavioral tests (Supplementary Figure 10B). No differences were observed in anxiety‐related behaviors or locomotion, however the percentage of headips in the EPM, a marker of risk assessment, was significantly higher in hM3D(Gq) mice, suggesting an increase in risk assessment compared to controls (Supplementary Figure 10D–F). Then we performed the VLT to investigate the defensive responses of the mice to a looming threat (Supplementary Figure 10G). hM3D(Gq) mice significantly escaped more than control mice at Day 1, despite having a plateau effect due to the already high escape probability observed in control mice (Supplementary Figure 10H,I). Surprisingly, the latency to escape in hM3D(Gq) mice was higher at Day 1 compared to control mice and tended to evolve in opposite directions along days for the two groups (Supplementary Figure 10J). No differences were observed in the max velocity during the escape between hM3D(Gq) mice and controls (Supplementary Figure 10K). To the contrary of Casp3 mice we did not observe any differences in the time spent in shelter or trigger zone or cumulated immobility (Supplementary Figure 10L–N). The traveled distance during the habituation of Day 1 was not affected either (Supplementary Figure 10O). Overall, chemogenetic activation of DRN_VIP_ neurons had an opposite effect on defensive behaviors in hM3D(Gq) mice compared to what we observed in Casp3 mice.

### 2.9 DRN_VIP_ Neurons Are Essential for Controlling Sleep Stability and Coordinating Risk Assessment and Defensive Responses

**FIGURE 8:**
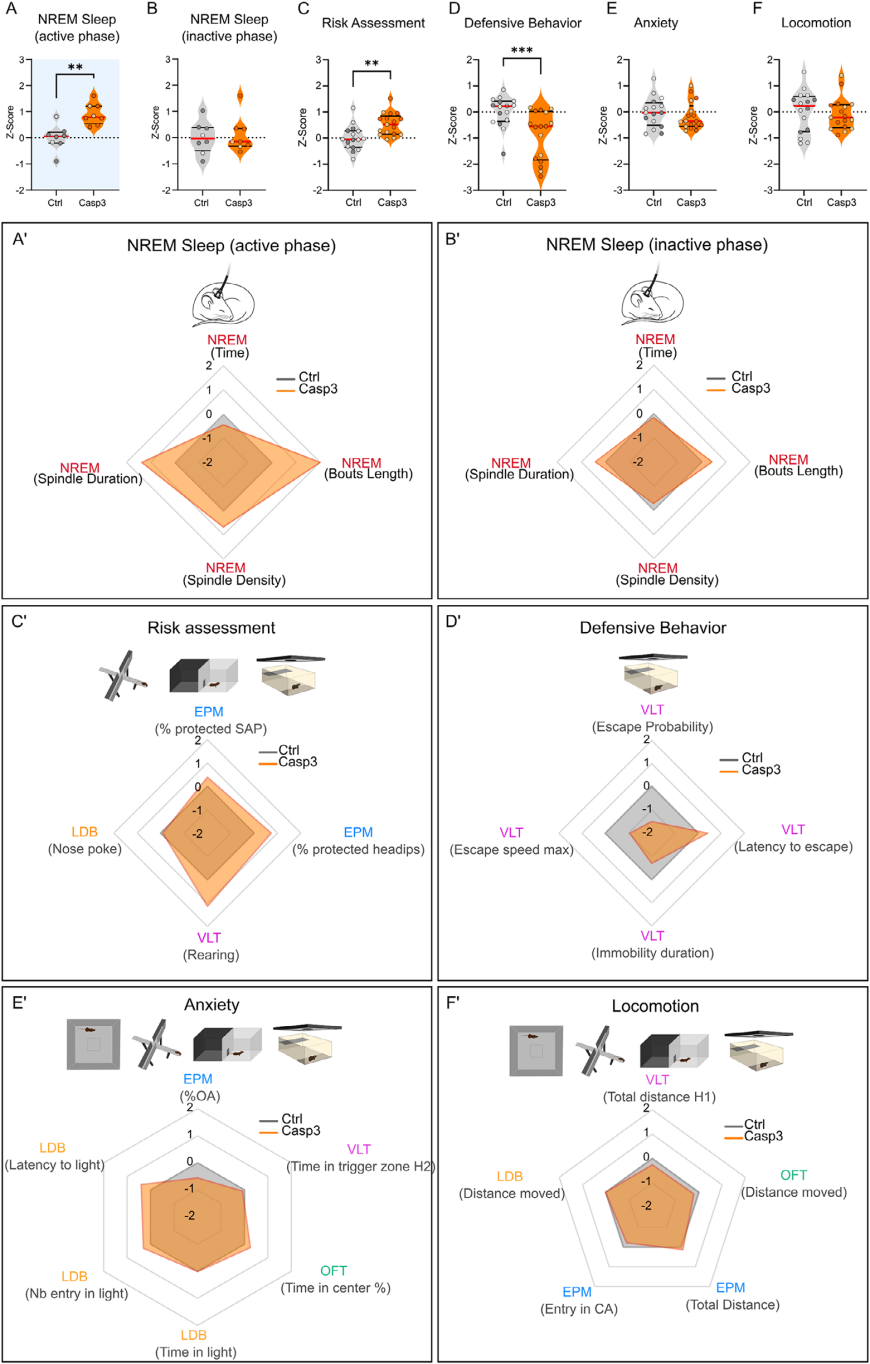
Scoring of the effect of DRN_VIP_ genetic ablation on behavior. Graph showing Z‐score of NREM sleep (active phase (A) and inactive phase (B)), risk assessment (C), Defensive Behavior (D), Anxiety (E) and Locomotion (F) measured from several variables from different behavioral tests as shown in A’ for NREM sleep (active phase) and B’ for NREM sleep (inactive phase), C’ for risk assessment, D’ for defensive behavior, E’ for anxiety and F’ for locomotion (NREM sleep (active phase), p‐value = 0.012; NREM sleep (inactive phase), T‐test, p‐value = 0.7542), Risk assessment, Unpaired T‐test, p‐value = 0.0075; Defensive behavior, anxiety and locomotion, Mann–Whitney, respective p‐values = 0.009, 0.4654 and 0.5334). Selected variables for each test are shown in parentheses below the test. Abbreviations: EPM: elevated plus maze; LDB: Light/Dark box; VLT: visual looming test; OFT: open field test; SAP: stretch attend posture; pHD: protected headips; H1: Habituation Day1; H2: Habituation Day2; OA: open arms; CA: closed arms.

To systematically evaluate behavioral changes following DRN_VIP_ neuron ablation, we computed z‐scores across multiple tests, categorizing behaviors into sleep stability, defensive behavior, risk assessment, anxiety, and locomotion (Figure 8) [57]. Interestingly, Casp3 mice exhibited a significant increase in NREM sleep specifically during the active phase (Figure 8A,A’), but not the inactive phase (Figure 8B,B’). Additionally, they showed a reduction in defensive behaviors together with an increase in risk assessment (Figure 8C,D,C’,D’). However, no significant differences were found in anxiety‐related behaviors, despite the reduced open‐arm time in the EPM or locomotion (Figure 8E,F,E’,F’). Together, these findings suggest that DRN_VIP_ neurons play a crucial role in controlling sleep stability and balancing threat assessment and defensive responses, ensuring an adaptive response to visual threats.

### 2.10 DRN_VIP_ Neurons Are Activated by Visual Threat‐Predictive Cues in Behaving Mice

**FIGURE 9:**
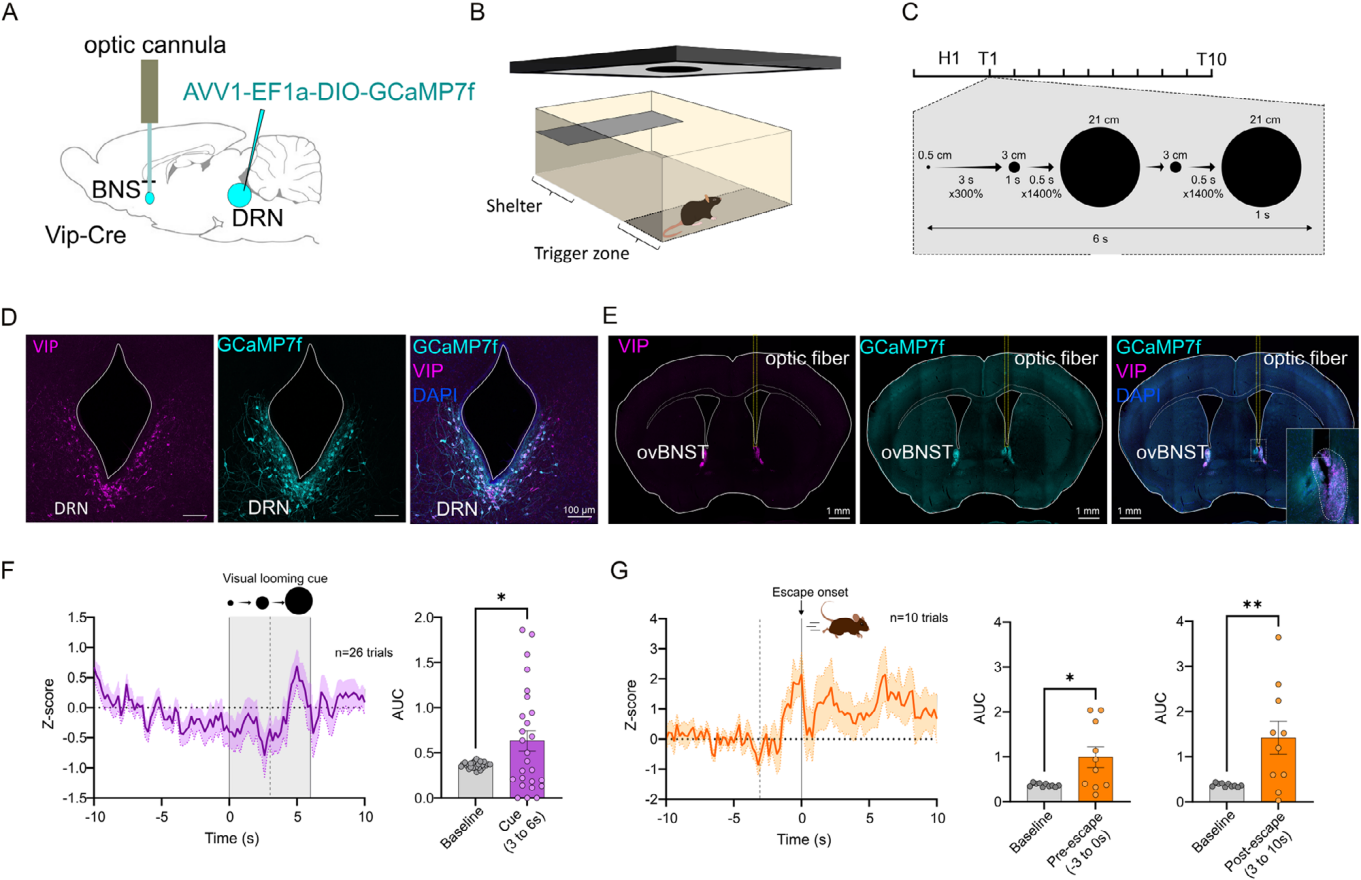
DRN_VIP→ovBNST_ calcium changes during the visual looming test. (A) schematic representation of the injection of AAV1‐EF1a‐DIO‐GCaMP7f virus in the DRN and implantation of an optic fiber in the ovBNST in Vip‐Cre mice. (B) Schematic representation of the visual looming test arena. (C) Description of the timeline of the test with the 5 min habituation period as H1, the 10 trials, and the detailed protocol for the visual cue. (D) Confocal images showing colocalization of GCaMP7f and VIP in DRN neurons. (E) Epifluorescent images of GCaMP7f and VIP fibers in the ovBNST with the optic fiber track in yellow and a close‐up image of the optic fiber location on the right image. (F) Graphs showing on the left the z‐score of the recorded fiber photometry signal averaged for 3 mice and 26 trials centered on the onset of the visual looming cue. The grey area represents the visual looming cue, and the dotted line is placed at 3s. On the right a comparison of the area under the curve between the baseline (−10 to −3 s before visual cue) and the period where most mice escape (3 to 6 s after the onset of the visual cue). (G) Graphs showing on the left the z‐score of the recorded fiber photometry signal averaged for 2 mice and 10 escapes centered on the onset of the escape. The dotted line is placed at −3s. On the right are comparisons of the area under the curve between the baseline (−10 to −3 s before visual cue) and the pre‐escape period (−3 to 0) and the post‐escape period (3 to 10 s). Graphs show mean ± SEM. *p‐value < 0.5, **p‐value < 0.01.

In order to investigate the specific moment DRN_VIP_ neurons are activated during the visual looming test, we recorded calcium changes at the level of DRN_VIP→ovBNST_ terminals during the visual looming behavioral task. We injected Vip‐Cre mice with an AAV1‐EF1a‐DIO‐GCaMP7f in the DRN and implanted an optic fiber in the right ovBNST (Figure 9A). The same VLT arena and visual looming cue protocol were used for the fiber photometry experiment as for the Casp3 and hM3D(Gq) (Figure 9B,C). Histological analysis confirmed the transfection of the GCaMP7f in DRN_VIP_ neurons and the correct location of the optic fiber (Figure 9D,E). Fiber photometry signal recorded in the ovBNST was significantly increased during the looming cue, specifically 3 to 6 s after the onset of the cue. This period corresponds to the moment where the visual looming cue size increases by 1400% two quick times (Figure 9C,F). When centering the fiber photometry signal on the onset of the escape, we observed a first peak just before the onset of the escape as well as a longer period with an increased signal after the escape (Figure 9G). The timing of the first peak suggests a role for the DRN_VIP→ovBNST_ neurons in alertness.

## 3 Discussion

Evaluating risks in unfamiliar, complex, or hazardous environments and selecting appropriate behavioral responses to mitigate potential threats are essential for animal survival and may be influenced by homeostatic sleep pressure [6]. In this study, we present a comprehensive analysis of a relatively unexplored subpopulation of DRN neurons located in the midbrain. Our findings show that, in both mice and primates, VIP‐expressing neurons represent a significant subset of DA neurons within the region encompassing the ventral periaqueductal gray and dorsal raphe. In mice, this population of approximately 500 DRN_VIP_ neurons, representing 40% of all DRN_DA_ neurons, is located in a crab‐claw‐shaped region beneath the cerebral aqueduct, occupying a volume of 0.155 mm^3^. This specific neuronal population is strategically positioned to regulate the central extended amygdala through a feedback circuit. It primarily receives input from PKC‐δ neurons in ovBNST and the CeA (lateral division). In turn, DRN_VIP_ neurons send axonal projections back to both the ovBNST and CeA (lateral division), where they modulate PKC‐δ neuronal activity via glutamate release. Selective genetic ablation of DRN_VIP_ neurons leads to several key effects: 1) increased electrophysiological activity in the BNST and CeA, 2) disruption of sleep architecture during the active phase, characterized by a decrease in the frequency of NREM episodes and an increase in their duration, which suggests enhanced sleep stability without a rise in total NREM sleep time or excessive daytime sleepiness, and 3) enhancement of risk assessment behaviors that may reflect dysfunctional processing, and 4) alteration of defensive responses. We conducted a fiber photometry experiment to monitor the activity of DRN_VIP_ neurons at the level of their axonal projections in the BNST. This approach allowed us to demonstrate direct activation of the DRN_VIP_ system during presentation of the visual threat‐predictive cue in behaving mice. These findings offer valuable insight into the neural circuits controlling sleep stability and underlying risk‐assessment behavior under normal physiological conditions.

### 3.1 DRN_VIP_ Neurons are Strategically Positioned to Influence the Central Extended Amygdala via a Feedback Loop

Our study revealed that DRN_VIP_ neurons represent a critical subset of DRN_DA_ neurons in mice and are also present in non‐human primates, thereby suggesting potential evolutionary conservation. It has been found that DRN_VIP_ neurons primarily project to the CeA and BNST [19, 29]. Our findings further reveal that the dorsolateral BNST (including the ovBNST and juxtacapsular BNST), anterior BNST, and the CeA (lateral division) are the principal target regions of DRN_VIP_ neurons, followed by the insular cortex and posterior basolateral amygdala. Our in situ hybridization results indicate that DRN_VIP_ neurons can be classified as a subset of DRN_DA_ neurons, given their colocalization with Th mRNA in over 80% of cases. Based on their molecular profile, three distinct subsets of dopaminergic neurons have been identified within the dorsal DRN [11]. Our findings further confirm this heterogeneity and reveal that DRN_VIP_ neurons constitute 40% of the total dopaminergic neuron population in the DRN. The classification of DRN_VIP_ neurons as dopaminergic has been a topic of recent debate [29]. Our observations in mice indicate that these neurons exhibit low levels of TH protein within the DRN, as evidenced by weak immunohistochemical labeling (Figure 1B). In contrast, TH expression is more prominent at their axonal terminals in the BNST and CeA, where a clear (∼50%) and significant reduction in labeling is observed in the Casp3 group (Figure 4). This pattern does not appear to hold in non‐human primates, where DRN_VIP_ neurons show robust TH expression within the DRN itself (Supplementary Figure 2).

Although there is a clear link between TH expression levels and the ability of dopamine neurons to synthesize and release dopamine, this relationship is complex and influenced by regulatory mechanisms as well as compartmental differences within neurons [58]. Further experiments will be required to determine whether the influence of DRN_VIP_ neurons on specific sleep states and defensive behavior is mediated by glutamate, VIP, dopamine, another transmitter, or a combination of these signaling mechanisms. Both the ovBNST and CeA (lateral division) are predominantly composed of GABAergic neurons, yet they are both highly heterogeneous in terms of cellular composition. For instance, PKC‐δ‐expressing neurons represent a distinct subpopulation with a similar organizational pattern in both the ovBNST and CeA (lateral division) [59]. Our findings reveal that DRN_VIP_ neurons are monosynaptically connected to PKC‐δ neurons and use glutamate as a neurotransmitter in both the ovBNST and CeA (Figure 4). Moreover, over 90% of the synaptic inputs to DRN_VIP_ neurons originate from the ovBNST and CeL, and approximately 95% in the ovBNST and in the CeA (lateral division) of the neurons synaptically connected to DRN_VIP_ neurons are PKC‐δ‐expressing cells (Figure 3). PKC‐δ neurons in the BNST and CeA (lateral division) are proposed to constitute a parallel microcircuit within the central extended amygdala [59, 60]. BNST PKC‐δ neurons are activated during risk assessment [36] and, together with CeA (lateral division) PKC‐δ neurons, may form a retrograde inhibitory pathway that acts as a brake to prevent over‐excitation and sustain the phasic neural activity of DRN_VIP_ neurons during risk assessment. Alternatively, it is likely that PKC‐δ neurons in the CeA (lateral division) and ovBNST are heterogeneous, with divergent projections in each structure. Additional experiments will be necessary to distinguish between feedback loop versus divergent projection organization. Finally, although PKC‐δ^+^ neurons in the CeA and BNST are major targets of DRN_VIP_ neurons (Figure 3), they are unlikely to be the sole mediators of the observed behavioral effects. Other cell types within these regions likely contribute, offering a plausible explanation for differences between our findings and prior studies focused solely on PKC‐δ^+^ neuron manipulation [34, 60]. Notably, we also demonstrated that DRN_VIP_ neurons occupy a central position, with the unique characteristic of individual neurons projecting to both the CeA (lateral division) and ovBNST (Supplementary Figure 4). In both regions, these neurons appear to exhibit coordinated activity and function as threat detectors in experimental paradigms that elicit defensive behaviors [35, 36]. Collectively, these findings suggest that DRN_VIP_ neurons play a critical role in threat evaluation and the initiation of defensive responses.

### 3.2 DRN_VIP_ Neurons Orchestrate Active‐Phase Sleep Architecture, Risk Assessment and Defensive Behaviors

Our findings indicate that caspase‐induced ablation of DRN_VIP_ neurons selectively alters NREM sleep architecture during the active‐phase, prolonging individual sleep episodes while reducing their overall frequency. Notably, total NREM sleep time remained unchanged, ruling out excessive daytime sleepiness. This suggests that DRN_VIP_ neurons contribute to shortening the duration of sleep bouts, and conversely that their loss enhances sleep stability. Given that sleep stability is crucial for modulating cognitive and neural processes involved in threat evaluation and adaptive defensive behaviors [6], DRN_VIP_ activity may serve as a regulatory mechanism balancing restorative sleep with wakefulness in dynamic environments. Previous work has demonstrated that DRN_DA_ neurons play a key role in arousal control, acting as a “gatekeeper” for wakefulness by promoting rapid awakening in response to salient stimuli [23]. In fact, accumulating evidence suggests that NREM sleep during the active phase differs qualitatively from NREM sleep during the inactive phase, likely due to enhanced vigilance mechanisms. For instance, phenomena such as the “first‐night effect” reveal interhemispheric asymmetries in sleep depth and vigilance during NREM sleep, pointing to a covert surveillance system that enables monitoring of the external environment even during sleep [61]. This modulation appears more prominent during states of heightened arousal or activity, and may underlie the adaptive variation in sleep architecture across behavioral contexts [61]. Our findings align with existing models of threat‐processing circuitry, particularly the BNST–CeA axis, which is known to gate sustained anxiety‐like states [62]. Furthermore, the literature supports an evolutionarily conserved adaptive compromise between defensive arousal and sleep homeostasis [63]. The anatomical reciprocity we observed may thus support a mechanism for dynamically reallocating neural resources between vigilance states during sleep and threat responsiveness.

Our results suggest that DRN_VIP_ neurons could function as a neuronal alarm system, reacting to threats or unexpected stimuli – such as movement or noise from conspecifics – that are more likely to occur during the active phase. By maintaining NREM sleep episodes short, these neurons may ensure heightened vigilance when environmental disturbances are most frequent. Consequently, in their absence, increased sleep stability emerges specifically during the active phase without affecting overall homeostatic sleep need (Figure 5).

Our behavioral tests rely on an approach/avoidance conflict, balancing the drive to explore novel areas against aversion to potentially threatening environments. These tests inherently involve risk assessment and threat evaluation as the animal decides whether to enter a potentially unsafe area [1, 48, 64]. The EPM has been validated to quantify risk assessment and anxiety‐like behaviors [48], with impaired risk assessment typically linked to increased time and more frequent entries into the open arms, reflecting heightened risk‐taking behavior [65]. Our study found that risk assessment behaviors (Figure 8) were affected in mice with genetic ablation of DRN_VIP_ neurons, consistent with the established role of DRN_DA_ neurons in detecting salient stimuli and assigning them positive or negative valence [25]. Moreover, the BNST and CeA, primary targets of DRN_VIP_ neurons, are central to risk assessment, valence attribution, and defensive behaviors [66]. Furthermore, it has been shown in humans that changes in BNST and CeA connectivity during threat anticipation have been associated with anxiety and risk evaluation processes [67, 68]. Together, these findings underscore the pivotal role of DRN_VIP_ neurons and their targets, in regulating risk assessment. They suggest that ablation of DRN_VIP_ neurons disrupts the threat detection system, leading to a dysfunctional alarm response that may drive increased risk assessment behavior in mice.

Overall, the genetic ablation of DRN_VIP_ neurons does not appear to affect the mice's general anxiety state, except perhaps in the EPM, where lesioned mice spend less time in the open arms (Figure 6D). While this outcome may seem counterintuitive, our findings suggest that disrupting DRN_VIP_ neurons – a key component of the risk assessment circuit – impairs information gathering, reducing risky behavior in the EPM and discouraging exploration of riskier areas. The visual looming test is highly effective at triggering defensive behaviors while also offering valuable insights into exploration and risk assessment, making it a useful tool for studying impaired information gathering [56]. Here, we show that in the visual looming test, selective genetic ablation of DRN_VIP_ neurons leads to increased risk assessment and impaired defensive behavioral responses. We interpret these findings as evidence that threat detection is disrupted by the loss of DRN_VIP_ neurons. As a result, mice may engage in more risk assessment behavior to compensate for reduced threat sensitivity, which ultimately leads to fewer escape responses. However, we cannot exclude the possibility that genetic ablation of DRN_VIP_ neurons also reduces general arousal, impairs the processing of salient environmental cues, or biases defensive strategies toward lower‐threat responses such as risk assessment rather than escape [23]. Here, we show that the DRN_VIP_→ovBNST pathway is rapidly and selectively activated during the most threatening phase of the visual looming stimulus, preceding escape onset (Figure 9). The precise timing of this activation supports a role for DRN_VIP_ neurons in mediating heightened alertness and triggering adaptive defensive responses. These results support our hypothesis that DRN_VIP_ neurons function as a neuronal alarm system, responding to threats or unexpected stimuli.

The looming test assesses the balance between exploration and safety, a critical component of risk assessment. Since risk assessment is a critical component in modulating escape behavior [69], the impaired escape behavior observed in mice lacking DRN_VIP_ neurons during the looming test could be influenced by an underlying maladaptive risk assessment behavior. Understanding which neural circuits transmit visual information to DRN_VIP_ neurons remains a key question. One potential answer lies in findings from mice, where retinal projections to the superior colliculus and the DRN are believed to play a key role in processing visual looming stimuli and mediating defensive behaviors [70, 71, 72]. Our results reveal that neurons in the superior colliculus form synaptic connections with DRN_VIP_ neurons (Figure 2B), supporting the idea that the superior colliculus may serve as a relay for visual information to DRN_VIP_ neurons.

In conclusion, our study provides novel insights into the neural circuitry underlying sleep stability, risk assessment, and defensive behaviors. Our findings underscore the importance of DRN_VIP_ neurons as central integrators of risk assessment circuits. The relevance of this circuitry extends beyond rodent models. Human studies link BNST and CeA connectivity to risk evaluation and anxiety processes, suggesting that similar neural mechanisms may underlie these behaviors across species. Importantly, our findings may provide valuable insights into autism spectrum disorder (ASD). Studies have shown that BNST synaptic functions are disrupted in an ASD mouse model [73], associated with abnormal risk‐assessment behaviors [74]. Additionally, a translational study reported that children with autism do not exhibit typical looming‐evoked defensive responses [75]. By elucidating the neural substrates of risk assessment, our study opens avenues for exploring targeted interventions in conditions like ASD, where these processes are disrupted.

## 4 Star Methods

### 4.1 Animals

C57BL/6JRj (≥ 8 week‐old; Elevage Janvier, France), Vip‐IRES‐Cre and Vip‐IRES‐Cre/COP4 mice were used. Vip‐IRES‐Cre and Vip‐IRES‐Cre/COP4 mice were maintained on a C57BL/6J genetic background. Animals were housed two to five per cage in standard temperature and humidity conditions (22‐23°C, 40% relative humidity, 12h light/dark illumination cycle) and had access to food and water ad libitum. Both female and male mice were used in this study. Female rhesus monkeys (Macaca mulatta: mean age = 5 ± 1years, mean weight = 5.3 ± 0.8 kg, n = 2) were euthanized with an overdose of pentobarbital (150 mg/kg). Brains were quickly removed after death, immediately frozen in dryice‐cooled isopentane, and then stored at −80 °C. The time between euthanasia and freezing of the brains was 10 min in all cases.

### 4.2 Double‐Probe Fluorescent In Situ Hybridization (sdFISH)

sdFISH was carried out as described previously (Dumas et al., 2019).

Probes: sdFISH was performed using antisense riboprobes for the detection of Vip mRNA: NM_011702.3 sequence 402–1320, Cre: AB449974.1 sequence 245–1060, and Th: NM_012740.4 sequence 456–1453. Synthesis of digoxigenin and fluorescein‐labeled RNA probes was made by a transcriptional reaction with incorporation of digoxigenin or fluorescein labeled nucleotides. Specificity of probes was verified using NCBI blast.

Procedure: C57BL/6J (N = 4) or Vip‐Cre (N = 5) mice were sacrificed by cervical dislocation. Brains were frozen in −30 to −35 °C cold 2‐methylbutane (≥99%, Honeywell) and stored at ‐80°C until sectioned with cryostat (Leica). 16 µm thin cryosections were cut and directly mounted on glass slides in a serial way (9 slices of DRN distributed on 8 slides), air‐dried, and kept at −80 °C until used. Slices were fixed in 4% paraformaldehyde and acetylated in 0.25% acetic anhydride/100 mM triethanolamine (pH 8). Sections were hybridized for 18 h at 65°C in 100 µL of formamide‐buffer containing 1 µg/ml digoxigenin‐labeled riboprobe (DIG) and 1 µg/ml fluorescein‐labeled riboprobe. Sections were washed at 65 °C with SSC buffers of decreasing strength and blocked with 20% fetal bovine serum and 1% blocking solution. Fluorescein epitopes were detected with horseradish peroxidase (HRP) conjugated anti‐fluorescein antibody at 1:5000 and revealed using Cy2‐tyramide at 1:250. HRP‐activity was stopped by incubation of sections in 0.1 M glycine followed by a 3% H_2_O_2_ treatment. DIG epitopes were detected with HRP anti‐DIG Fab fragments at 1:2000 and revealed using Cy3 tyramide at 1:100. Nuclear staining was performed with 4' 6‐diamidino‐2‐phenylindole (DAPI).

Data Analysis: All slides were scanned at 20× resolution using the Hamamatsu NanoZoomer S60 Digital slide scanner (Hamamatsu). Laser intensity and time of acquisition were set separately for each riboprobe. Images were analyzed using the NDP.view2 software (Hamamatsu Photonics). Regions of interest were identified according to the Paxinos mouse brain atlas. Positive cells refer to a staining in a cell body clearly above the background and surrounding a DAPI‐stained nucleus. Colocalization was determined by the presence of the signals for both probes in the soma of the same cell.

### 4.3 Stereotaxic Injections

Surgeries for behavioral experiment (Caspase3 and excitatory DREADDs), anatomy (synaptophysin and rabies), and in vivo electrophysiology were performed as follows: mice were anesthetized with a mixture of air/isoflurane (maintained at 1–2% isoflurane, 1 min L^−1^) and placed in a stereotaxic frame. After receiving subcutaneous doses of Meloxicam (20 mg/Kg) and Buprenorphine (0.1 mg/Kg) and a local injection of Lurocaine (7 mg/Kg) the skin was incised. Coordinates for targeting the following structures were: BNST, +0.15 mm from Bregma, −0.85 mm from sagittal vein and −3.85 mm from brain surface; CeA, −1.65 mm from Bregma, −0.85 mm from sagittal vein and −3.85 mm from brain surface; DRN, −4.20 to −4.60 mm from bregma, −1.00 mm from sagittal vein and −2.80 for targeting VIP neurons or −3.00 for targeting the whole DRN dopaminergic neurons, angle: 20° in the coronal plane. Injected volumes were 3 × 300 nL for AAV‐EF1‐DIO‐Caspase3 and control injections in the DRN, 2 × 240 nL for AAV1‐EF1a‐DIO‐hM3D(Gq)‐mCherry and control virus, 3 × 180 nL for AAV1‐EF1A‐DIO‐synaptophysin‐GFP, helper virus (AAV1‐EF1a‐DIO‐TVA950‐T2A‐cvs11G‐WPRE, a bicistronic construct that expresses only the fusion protein TVA‐T2A and the glycoprotein CVS11G), modified helper virus (AAV1‐EF1a‐DIO‐TVA950‐T2A‐WPRE, a monocistronic construct that expresses only the fusion protein TVA‐T2A) and tracing virus used for brain clearing (AAV5‐EF1a‐DIO‐eYFP) in the DRN, 1 × 250 nL for rabies injections in DRN (PSADB19dG‐mCherry) and 120 nL for rabies injections in ovBNST (PSADB19dG‐GFP) and CeA (PSADB19dG‐mCherry) (Table 1).

### 4.4 Surgery for Fiber Photometry and Optogenetics Experiments

Vip‐Cre mice (N = 5 for fiber photometry, N = 18 for optogenetics) were anesthetized with a mixture of air/isoflurane (maintained at 1–2% isoflurane, 1 min L^−1^) and placed in a stereotaxic frame. After receiving subcutaneous doses of Meloxicam (20 mg/Kg) and Buprenorphine (0.1 mg/Kg) and a local injection of Lurocaine (7 mg/Kg), the skin was incised. Injection of 2 × 180 nL of AAV1‐EF1α‐DIO‐jGCaMP7f (VVF ETH Zurich, cat# v398‐1) for fiber photometry experiment and AAV5‐EF1a‐DIO‐eNpHR‐mCherry or AAV5‐EF1‐DIO‐eYFP for optogenetics experiment, were done in the DRN at the following coordinates: AP: −4.30 and −4.60 mm from bregma, ML: −0.75 mm from sagittal vein, DV: −2.70 mm from brain surface and 20° angle in the frontal plane. During the same surgery, mice were implanted with an optic fiber in the right ovBNST at the following coordinates: AP: +0.15 mm from bregma, −0.85 mm from sagittal vein and −3.60 mm from brain surface (200 µm core diameter, 0.39 NA, 5 mm long, Doric Lenses) for fiber photometry experiment. For the optogenetics experiment, an optic fiber was implanted in the DRN at the following coordinates: AP: −4.50 mm, ML: 0.60 mm, and DV: −2.45 mm, 15° angle in the frontal plane (400 µm core, 0.5NA, 5 mm long, Doric Lenses). Once the skull was dried and the optic fiber in position, a first layer of air‐dried opaque cement (Superbond Universal Kit, Sun Medical) was applied. We then applied a UV‐responsive layer of cement (Filtek Supreme Flowable 3M, Solventum).

### 4.5 Immunohistochemistry

Mice were first anesthetized with a mixture of isoflurane and air (4%, v/v isoflurane/air) and received s.c injection of Meloxicam, followed 10 min later by i.p injection of a lethal dose of Euthasol (300mg/Kg). After perfusion with 1X Phosphate‐buffered saline (PBS), or 1X PBS followed by 4% formaldehyde for fiber photometry animals, brains were extracted and post‐fixed in 4% formaldehyde for at least 24 hours. 40 µm coronal brain slices were cut at the vibratome and transferred to 1X PBS wells. After 1X PBS washes an antigen retrieval step was performed for immunohistochemistry labeling VIP and PKC‐δ. Afterward, slices were incubated in a blocking solution for 90 min (5‐10% normal goat serum in PBS‐TritonX100 0.3% (S26‐100ML, EMD Millipore Corp.). Directly after, slices were incubated in the primary antibody solution for 72 hours (Table 1): for the histological analysis of the Casp3 behavioral group: guinea‐pig anti‐TH (1:2000, SYSY 213104) and rabbit anti‐VIP (1:1000, ab 272726 Abcam); for control behavioral group (synaptophysin‐GFP mice), chicken anti‐GFP (1:1000, GFP‐1020 Aves), guinea‐pig anti‐TH, and rabbit anti‐VIP. For excitatory DREADD behavioral experiment: rat anti‐RFP (1:1000, 5f8 Chromotek) and rabbit anti‐VIP (1:1000). For fiber photometry experiment: chicken anti‐GFP (1:1000) and rabbit anti‐VIP (1:1000). For optogenetics experiment: rat anti‐RFP (1:1000, 5f8, Chromotek). For modified rabies experiments, chicken anti‐GFP (1:1000), rat anti‐RFP (1:1000) and rabbit anti‐VIP (1:1000); for rabies experiments, mouse anti‐PKC‐δ (1:500, 610398 BD Biosciences), rat anti‐RFP, rabbit anti‐somatostatin (1:1000, NBP1‐87022 Novus) for BNST and CeA or rabbit anti‐2A peptide (1:1000, ABS31 Sigma), guinea‐pig anti‐TH for the DRN; for patch‐clamp experiments, chicken anti‐GFP, mouse anti‐PKC‐δ for BNST slices or chicken anti‐GFP and rabbit anti‐VIP for DRN slices. Slices were washed in 1X PBS and incubated in a secondary antibody solution (1:500 in 1X PBSTritonX‐100) for all secondary antibodies (Table 1) for 2h containing antibodies matching the primary antibodies and desired fluorescent reporter listed here in the same order as above: goat anti‐guinea‐pig A568 (A11075 Life technologies), and goat anti‐rabbit A647; goat anti‐chicken A488, goat anti‐guinea‐pig A568 and goat anti‐rabbit A647; goat anti‐chicken A488, goat anti‐rat A568 and goat anti‐rabbit A647; goat anti‐mouse A488, goat anti‐rat A568, goat anti‐rabbit A647; goat anti‐rabbit A488, goat anti‐guinea‐pig A568; goat anti‐chicken A488, streptavidin A568 (1:1000), goat anti‐mouse A647; goat anti‐chicken A488, streptavidin A568. Afterward slices were washed in 1X PBS, incubated in Hoestch solution for 5 min (1:10 000, 33258 Invitrogen), washed again, and mounted on glass slides and coverslipped (Fluoromount‐G, Sigma). Acquisitions were performed using either a Nanozoomer scanner (Hamamatsu), an epifluorescent microscope (Evident), or confocal microscope (Leica).

For non‐human primate immunohistochemistry, double immunofluorescence staining was performed to visualize TH and VIP immunoreactivity simultaneously. Briefly, 50 µm free‐floating sections were first washed in PBS and incubated in a blocking buffer containing 0.3% Triton X‐100 and BSA (1/50 in 0.1 M PBS) to prevent nonspecific staining. The sections were then incubated overnight at room temperature with a mixture of primary antibodies diluted at 1:1000 against TH and VIP (guinea pig anti‐TH, Synaptic system; rabbit anti‐VIP, Abcam). After thorough rinsing in PBS, the sections were incubated for 1 hour at room temperature with a cocktail of corresponding secondary antibodies conjugated to either Alexa Fluor 488 or 568 fluorophores (Molecular Probes). Following PBS washes, the sections were mounted on gelatin‐coated slides and incubated for 5 minutes in a 0.3% Sudan Black B/70% ethanol solution to reduce tissue autofluorescence. Counterstaining was performed by immersing the slides in Hoechst solution diluted in PBS for 5 minutes. Finally, the slides were coverslipped using a fluorescence mounting medium (Fluoromount‐G, Sigma). Images were acquired using a Pannoramic Scanner II (3D Histech) at 20X magnification in extended mode, with five layers spaced 1.4 µm apart.

### 4.6 Histological Analysis

Casp3 behavioral experiment quantification: Slices containing DRN, ovBNST, and CeA were used to quantify the amount of VIP and TH neurons (DRN), and fiber mean grey value (ovBNST and CeA) on images acquired with Leica confocal microscope SP5‐MP at 20X magnification. Counting of VIP and TH neurons in the DRN was done manually using Fiji software. One slice of an intermediate level of the DRN was selected for each mouse. The mean grey value in the ovBNST and CeA was measured on 20 µm z‐stack images using Fiji software.

Excitatory DREADD behavioral experiment: DRN brain slices from mice injected with AAV1‐EF1a‐DIO‐hM3D(Gq)‐mCherry were checked at an epifluorescent microscope to verify the presence of the mCherry fluorescent reporter. Confocal images were acquired to confirm colocalization of VIP and mCherry in the DRN.

Quantification of DRN_VIP_ neurons inputs: After immunolabeling for PKC‐δ and amplifying rabies reporter with RFP antibody, images of coronal brain slices were acquired using either an epifluorescent microscope (Evident) for quantifying whole brain inputs to DRN_VIP_ neurons (xy mosaic images of whole slices at 10X magnification) or confocal microscope (SP5‐MP, Leica) for quantifying colocalization of RFP and PKC‐δ in the ovBNST and CeA (20X magnification). Whole brain quantification of DRN_VIP_ neurons was performed by using Fiji/QuPath software and an experimental plugin “Aligning Big brains and atlases” (ABBA) to apply the Allen Brain atlas classification to the brain slice images [76]. RFP positive neurons were manually labeled on QuPath. The injection site (DRN) and the closely surrounding regions were excluded from the quantification. Structures with a mean density of neurons below 5 × 10^−7^ were excluded from Figure 3B. Fiber photometry: the presence of GCaMP7f‐positive neurons colocalizing with VIP in the DRN was verified at a confocal microscope. In the BNST, a visible track allowed the localization of the optic fiber in all the mice, but two of them were excluded because of an anterior location.

### 4.7 Brain Clearing

Brain clearing of the three brains has been done following Adipoclear protocol including several delipidation steps and GFP amplification [77]. Whole brain acquisitions have been done using UltraMicroscope II from Biotech–Milteniy with a 1.26X magnification. Before whole brain quantification, fluorescence of injections sites has been measured and compared at 3 ventro‐dorsal planes of the DRN per mouse (Supplementary Figure 3). Stack of acquisitions has been automatically aligned through the Napari viewer following elastix registration to adapt the acquired brain to the Allen brain atlas. Counting of VIP+ cell bodies has been done using spot detection in Imaris software, with a diameter filter of 20um. Mean grey value quantification of each region of the Allen brain atlas has been done with a homemade plugin on Fiji. Whole brain quantification histograms have been obtained after min‐max normalization of the dataset composed of brain regions with a signal intensity superior to 0.25% of total brain intensity (Figure 2).

### 4.8 Ex Vivo Electrophysiological Recording

Brain slice preparation: The brain was quickly removed from the skull and mounted on the stage of a vibratome (VT1200; Leica Microsystems), immersed in a cutting solution at 4°C saturated with carbogen (N = 4 mice for ovBNST slices, N = 3 mice for CeA slices, and N = 1 mouse for DRN slices). It was then sectioned into 300 µm thick slices in the coronal plane. Slices containing the ovBNST, CeA, and DRN were then transferred to a holding chamber filled with standard carbogen‐saturated ACSF and placed in a water bath at 32 °C for 1 hour. The ACSF was composed of (in mM) NaCl (126), KCl (2.5), NaH_2_PO_4_·H_2_O (1.25), CaCl_2_·H_2_O (2), MgSO_4_·7H_2_O (2), NaHCO_3_ (26), D‐glucose (10), glutathione (5), and sodium pyruvate (1). The pH of the ACSF was adjusted to 7.4, and its osmolarity was between 310 and 315 mOsm. The slices were then maintained in this solution at room temperature until electrophysiological recordings.

Electrophysiology: The slices were then transferred one by one to the recording chamber of an upright microscope (AxioExaminer Z1; Zeiss) and continuously perfused with equilibrated ACSF heated to 32°C. In some experiments, AMPA/NMDA receptor antagonists, DNQX (50 µM) and D‐APV (20 µM), were also used to demonstrate the glutamatergic nature of synaptic currents recorded in the ovBNST. Tetrodotoxin (TTX – Voltage gated sodium channel blocker) at 1µM and 4‐aminopyridine (4‐AP – Voltage gated potassium channels blocker) at 50 µM are also used to show the monosynaptic nature of the connection between DRN_VIP_ and ovBNST or CeL. Neurons were visualized using a 63X water immersion objective (APO‐CHROMAT; Zeiss) with differential interference contrast (DIC) illumination or an epifluorescence system (HXP120; Kubler). Recordings of ovBNST neurons were made using patch electrodes made from borosilicate glass capillaries (GC150F10; Phymep) on a horizontal puller (Model P97; Sutter instruments) to obtain a pipette resistance of 5–8MΩ. In this project, the intracellular solution used is “KGluconate” which served for whole‐cell recordings in current‐clamp mode. It is composed of (in mM) KGluconate (135), NaCl (3.8), MgCl2 6H2O (1), HEPES (10), EGTA (0.1), Na2GTP (0.4), Mg1.5ATP (2). This solution also contains biocytin (2 mg/ml) and has a pH of 7.25 and an osmolarity of 290–295 mOsm3. Electrophysiological signals were amplified and filtered with a 4KHz low‐pass filter using a Multiclamp 700B amplifier (Molecular Devices), then digitized at 20 kHz (Digidata 1550, Molecular Devices) using Clampex 10.7 software (Molecular Devices). Whole‐cell and voltage‐clamp recordings were performed at a holding potential of −60 mV, and glutamatergic EPSCs evoked by optogenetic stimulation of VIP neurons in the DRN were measured. Their amplitude and kinetics were measured following a train of 10 light pulses at 10 hertz. After electrophysiological recordings, the slices were fixed overnight in a 4% paraformaldehyde (PFA) solution, then stored at 4 °C in a 0.03% PBS‐sodium azide solution until immunohistochemical processing.

### 4.9 In Vivo Single‐Cell Electrophysiological Recording

BNST and CeA neurons were recorded in Casp3 (N = 17 for ovBNST and N = 15 for CeA) and control mice (N = 15 for ovBNST and N = 5 for CeA). Mice were first anesthetized with a mixture of isoflurane, air, and oxygen (4% isoflurane for anesthesia induction and maintained at 1‐1.5% isoflurane 1 min L^−1^ 50% air/oxygen). Mice were placed on a stereotaxic frame and a subcutaneous injection of a local analgesic (lidocaine, 7 mg/Kg) was done at the skin incision location. A craniotomy was made above the BNST and CeA on the right hemisphere. Recording glass micropipette (GC150F‐10, Harvard Apparatus) was filled with 2% pontamine sky blue solution in 0.5 M sodium acetate and lowered into either BNST or CeA. Single‐cell extra‐cellular recordings were performed in these two structures for Casp3 mice and controls. Single‐neuron spikes were amplified with an Axoclamp2B (300 Hz / 0.5 kHz), filtered (AM system), and collected online (CED 1401, Spike2, Cambridge Electronic Design). Validation of the correct location of the recordings was done a posteriori from the pontamine sky blue spot. The blue spot marking was performed on the last neuron of the final recording track. This marking was then used as a reference point to reconstruct the locations of all recorded neurons and to validate their correct positioning within the BNST or CeA.

### 4.10 Electrode Implant for Sleep Recording

After AAV‐EF1‐DIO‐Caspase3 and control injections in DRN, Vip‐Cre mice (N = 8 per group) were implanted with micro‐screws over the left parietal cortex (AP −2.0 mm, ML 1.8 mm from bregma) for cortical activity recordings and the occipital bone as a reference. Stainless‐steel electrodes with Teflon insulation (Model 791400, A‐M Systems Inc., Carlsborg, WA, USA) were implanted in the neck muscles to record electromyographic (EMG) signals. The electrodes and micro‐screws were then soldered onto a Straight Male PCB Header‐4 connector, which was secured to the skull with dental cement and acrylic.

### 4.11 Polysomnographic and Behavioral Data Acquisition

EEG and EMG signals were amplified at a 5000× gain and processed with a bandpass filter – applying a 1 Hz high‐pass for all channels and setting low‐pass filters at 3 kHz for EEG and 500 Hz for EMG – using signal conditioners (ERS100C and EMG 100 C amplifiers, Biopac MP160). These signals were digitized at a 6250 Hz sampling rate via AcqKnowledge software (v4.1, Biopac Systems) and stored for subsequent offline analysis. Each mouse underwent two consecutive recording sessions over a three‐day period. On the first day, animals were acclimated to the recording environment and the apparatus from 10:00 to 16:00 h (the light‐on, inactive phase). Recording started the following day at 18:00 h and continued until 19:00 h the next day. The complete 24‐hour recording cycle consisted of 12 h of lights‐off (from 19:00 to 7:00 h, corresponding to the active period) followed by 12 h of lights‐on (from 7:00 to 19:00 h, representing the inactive period), with the initial hour (from 18:00 to 19:00 h) discarded to minimize stress‐induced artifacts. For each session, four freely moving mice from the same home cage were placed in individual cages outfitted with bedding, nesting material, food, and water. Behavioral activity during polysomnography was captured using 1080p Logitech Brio webcams, and the EEG, EMG, and behavioral data were merged into a single video file at 10 frames per second using OBS Studio (v27.0.1).

### 4.12 State Sorting

The 24‐hour polysomnography and behavioral recording period was manually divided into 10‐second segments, each classified as AWAKE, NREM, or REM sleep. AWAKE was identified by low‐amplitude, desynchronized EEG signals paired with continuous EMG activity, whether during movement or rest. NREM sleep was characterized by an increase in EEG amplitude dominated by slow waves, low EMG tone, and a lack of movement. In contrast, REM sleep was marked by immobility, consistent theta‐dominated EEG patterns, and virtually no EMG activity. The placement of a cortical electrode over the dorsal hippocampus enabled optimal detection of theta oscillations and distinction between REM and NREM stages. This integrated approach allowed for accurate sleep state classification and a detailed analysis of sleep architecture.

### 4.13 Analysis of Sleep Architecture and EEG Microstructure

A custom MATLAB script was used to analyze sleep architecture and cortical activity. The duration of AWAKE, NREM, and REM states was calculated over the full 24‐hour cycle by segmenting data into 1‐hour bins and then averaging these values over 12‐hour periods based on the light conditions (19:00–07:00 for lights off and 07:00–19:00 for lights on). NREM sleep fragmentation was evaluated in 1‐hour bins by counting the number and measuring the duration of sleep bouts, with the results averaged over 12‐hour periods.

Cortical EEG signals were processed using standard MATLAB functions. Initially, a digital low‐pass infinite impulse response (IIR) filter (implemented with MATLAB's designfilt function set to a 625 Hz cutoff) was applied to limit the frequency range, and the signal was then down‐sampled to 1250 Hz. The signal was divided into 10‐second segments, and for each epoch, the power spectral density was estimated using the pwelch function (with no overlap and 1 Hz steps). The power for each frequency bin was normalized by dividing by the total power of that epoch, and these normalized values were categorized into specific frequency bands: delta (1–4 Hz), theta (6–9 Hz), sigma (10–15 Hz), beta (16–30 Hz), and gamma (40–80 Hz). Each 10‐second EEG segment was subsequently classified as AWAKE, NREM, or REM sleep based on manual scoring.

Spindle analysis was performed with a custom MATLAB script [78]. In brief, contiguous NREM EEG segments were filtered using a bandpass IIR filter (via designfilt) to isolate the spindle sigma frequency range. The signal envelope was then computed using a 700 ms moving root‐mean‐square (RMS) window, and the RMS value was cubed. Spindles were identified as events exceeding twice the cubed RMS average (calculated over the entire NREM period) with durations between 0.5 and 5 seconds. Finally, spindle density (number of events per minutes) and duration (in seconds) were quantified across the full 24‐hour cycle during NREM sleep.

### 4.14 Anxiety‐Related Behavioral Tests for Casp3 Experiment

Casp3 (N = 19) and control (N = 19) mice performed an open field test (OFT), a light/dark box test (L/D box), and an elevated plus maze test (EPM) described below. Prior to the first test, mice were handled progressively for three days. Before each test, mice were habituated to the experimental room for 30 min. Video acquisition was done using Polyvision software (Imetronics) and video analysis was performed by using Ethovision XT17 (Noldus) or Deeplabcut software. Number of mice for L/D box and EPM tests was N = 19 controls and N = 19 Casp3 mice and number of mice for the OFT was N = 17 controls and N = 20 Casp3 mice.

### 4.15 Open Field Test

The test consists of a square arena (40 × 40 × 40) where the mouse can freely explore for 10 min. The arena is artificially divided into zones: center, borders, and corners. Traveled distance, velocity, and time in the different zones were measured. The ROUT method was used on the “Time spent in center” variable to exclude potential outliers.

### 4.16 Elevated Plus Maze

The apparatus consists of an elevated plus shape platform with two closed arms (25 cm walls) and two open arms (30 cm long, 5 cm wide). The mouse is placed in the center facing an open arm and can freely explore the arena for 10 min. Measured variables were time spent in open arms, closed arms, and center, protected and unprotected headips, and stretch attend postures (SAP) as well as rearing. Headips and SAP were quantified manually. Protected zones correspond to the closed arms and center area, while unprotected zones correspond to the open arms. ROUT method was used on the “time spent in open arms” variable to exclude potential outliers.

### 4.17 Light/Dark Box

The arena is composed of two equally sized compartments (20 × 20 × 20 for each compartment), a light compartment strongly lit up (900–1000 lux), and a dark compartment. Both compartments are connected through a little aperture. The mouse is placed in the light compartment and can freely explore the arena for 10 min. Time spent in compartments, latency to light compartment, entries to the light compartment, and nose pokes to the light compartment were measured. The ROUT method was used on the “time spent in the light compartment” variable to exclude potential outliers.

### 4.18 Visual Looming Test

The visual looming protocol was adapted from [55]. The visual looming test (VLT) setup consists in a transparent rectangular arena (36 × 19.7 × 20cm) divided into three zones: a shelter zone (12.7 × 19.7 cm) which has a roof over, an intermediate zone (13.3 × 19.7 cm), and a trigger zone (9.9×19.7 cm). A screen was placed about 30 cm above the ground of the cage with a light grey background. Mice (N = 19 controls and N = 19 Casp3 mice) had three days of habituation to the apparatus, 5 min every time (one day of collective habitation two to three mice together and 2 days of individual habituation). On day 1, the mouse was placed in the trigger zone and could freely explore the arena during 5 min. After 5 min of habituation (corresponding to H1 for Day1, H2 for Day2, and H7 for Day7), a visual cue, which corresponds to a trial, was manually triggered on the screen every time the mouse entered the trigger zone. A refractory period of 30 s was set after each visual cue. The test was considered completed either when the mouse performed 10 trials or after 60 min. The same protocol was done on days 2 and 7. The visual cue consists an expanding black circular region (0 grayscale intensity) presented against a mid‐gray background (127 grayscale intensity). The Michelson contrast was calculated using the formula C = ((I_max_ − I_min_)/(I_max_ + I_min_))×100, resulting in a contrast of 100%. The cue increases from 0.5 to 3 cm in diameter over 3 seconds (corresponding to an area expansion rate of 2.291 cm^2^/s). It then expands twice from 3 to 21 cm in 0.5 seconds, corresponding to an area expansion rate of 678.6 cm^2^/s. The whole duration of the visual cue lasted for 6 s. From the center of the arena, the visual angle covered by the circle is approximately 0.9° at the onset of the cue (0.5 cm diameter), 5.7° when the circle reaches 3 cm in diameter, and finally 38.6° at the end of the cue (21 cm diameter). The test was filmed using a GoPro camera and analyzed with DeepLabCut and Python scripts (Aquineuro) for the following parameters: escape probabilities, time spent in zones, latency to escape, distance traveled, velocity, and immobility were measured manually for the rearing. Trials had to fulfill three criteria to be considered as escaping trials: a speed above 35 cm/s, a head orientation towards the shelter, and the mouse position in the shelter in at least 7 s after the onset of the visual cue. Escape probability corresponds to the number of escapes divided by the number of trials. A mouse was considered immobile when velocity was below 1 cm/s for at least 1 s. Additionally, the vigor of the “escape” was measured by lowering the velocity threshold of the “escape” to 20 cm/s in order to measure the vigor of the return to the shelter. For rearing quantification, the before cue period includes 30 s before trials 3, 4, 5, 6, and 7, while the during cue period includes 30 s after trials 1, 2, 3, 4, and 5. ROUT method was used on the “rearing” variable to exclude potential outliers.

### 4.19 Fiber Photometry Recordings during Visual Looming Test

Fiber photometry recordings were performed 8 weeks after surgery. The visual looming test was performed as described above to the exception of the duration of test, which was lowered to 30 min maximum per mouse in order to prevent important photobleaching. Before starting the behavioral test, the efficiency of the optic fiber was assessed, and the power of the LED was adjusted to obtain enough power at the tip of the optic fiber: For the 470 nm LED: 100 µW, for the 405nm: 35µW. The bulk fluorescence activity of DRN_VIP→ovBNST_ fibers was recorded via the expression of the GCaMP7f with Doric fiber photometry systems. Light was transmitted to the optical fiber through two light emitting diodes, using alternating emission of 20 Hz of the 405 and 470 nm wavelengths. The 470 nm LED was used to record activity changes, as the GCaMP optimal excitation wavelength is 470 nm, and the 405 nm LED was used as our control signal, to remove unrelated signal artifacts (such as motion artifacts) as this isosbestic wavelength is calcium independent. The LEDs were coupled to optical fiber patch cords (400 µm, NA: 0.57), which were connected to the optical fiber ferrules (200 µm, NA: 0.39). Fiber photometry recordings started at the end of the 5 min habituation period and was stopped either after 10 trials or after 25 min. To synchronize the video with the fiber photometry recordings, the camera was externally triggered by uEye Cockpit software, which emitted a TTL for each frame to the Doric Neuroscience Studio. Camera recordings were done at 20 fps and at 1280 × 1024 resolution.

To analyze fiber photometry signals we first determined the fractional ΔF/F, a least‐squares linear fit was applied to the 405 nm control signal and fitted to the 470 nm signal over the whole behavioral recording. This was done to align the two signals and correct for motion artifacts, auto‐fluorescence, and photo‐bleaching. It was calculated as follows: ΔF/F = ([470 nm signal − 405 nm fitted]/405 nm fitted). Then we down‐sampled the signal from 20 to 5 Hz and calculated a z‐score of the ΔF/F from the baseline. The baseline corresponds to a window from −10 to −3 s before the observed event. The observed event was either the onset of the visual cue or the onset of the escape. Different periods centered on the event were compared to the baseline by calculating areas under the curve (AUC) using GraphPad Prism 10 software (Threshold minimum peak height:10%, Total positive peak area was used). Unpaired t‐test or Mann–Whitney test was used to compare AUC from baseline to the different periods.

### 4.20 Optogenetics in the Visual Looming Test

Vip‐Cre mice injected with an inhibitory opsin (eNpHR) and control mice (Ctrl) were tested in the visual looming test. The behavioral procedure was identical to the one described above, with 3 days of habituation followed by three test days, Days 1, 2, and 7. Mice were habituated to the connection to the patch cord during habituation days. On test days, optogenetic inhibition was induced through a 594 nm yellow laser (MGL‐G‐594, CNI) at the onset of the visual cue and during its whole duration (6 s, 10 mW, continuous). The synchronization of the laser and the visual cue was done using a photodetector on the screen detecting the visual cue, which sends a TTL signal to an Arduino card connected to the laser. Mice behavior was recorded with a GoPro camera, and escape probability, velocity, latency, and time in zones were automatically analyzed with DeepLabCut and Python scripts (Aquineuro).

### 4.21 Behavioral Tests for Excitatory DREADDs Experiment

Vip‐Cre mice injected with either AAV1‐EF1a‐DIO‐hM3D(Gq)‐mCherry virus (N = 14) or control virus AAV1‐EF1a‐DIO‐mCherry (N = 19) went through the same series of behavioral experiments as the Casp3 mice: OFT, EPM, L/D box. The EPM tests was, however, slightly different with the addition of 0.5 cm edges on the open arms in order to increase the entries in the open arms. 30 min before each behavioral test, both hM3D(Gq) and control mice received an intraperitoneal injection of clozapine N‐oxyde (1 mg/Kg, Tocris Biosciences).

### 4.22 Sleep, Risk Assessment, Defensive Behavior, Anxiety, and Locomotion Z‐Score Calculation

To assess behavioral dimensions such as sleep, risk assessment, defensive behavior, anxiety, and locomotion, we employed the integrated behavioral z‐scoring method as described by [57]. This approach standardizes individual behavioral measures by calculating z‐scores, which represent the number of standard deviations a data point deviates from the mean of a control group. Specifically, for each behavioral parameter, the raw data were transformed into z‐scores using the formula: z = (X – μ) / σ, where X is the individual score, μ is the mean of the control group, and σ is the standard deviation of the control group.

For the calculation of the NREM Sleep score during the active and inactive phases, the following raw data were used: time spent in NREM sleep, spindle duration during NREM, spindle density during NREM, and bout length during NREM sleep. The risk assessment score was derived from the percentage of protected stretch‐attend postures (SAP) in the elevated plus maze (EPM), the number of nose pokes in the light‐dark box (LDB), the number of rearings in the visual looming test (VLT), and the percentage of protected head dips in the EPM. Defensive behavior was assessed using raw data from the VLT, including escape probability, maximum escape speed, immobility duration, and latency to escape. The anxiety score was calculated using the percentage of time spent in the open arms of the EPM, latency to enter the light compartment in the LDB, number of entries into the light compartment in the LDB, total time spent in the light compartment in the LDB, percentage of time spent in the center in the open field test (OFT), and time spent in the trigger zone during habituation on day 2 of the VLT. Lastly, the locomotion score was derived from total distance moved in the VLT during habituation on day 1, distance moved in the LDB, number of entries into the closed arms of the EPM, total distance traveled in the EPM, and distance moved in the OFT.

**TABLE 1:** Key resources table.

Reagent or resource	Source	Identifier
**Antibodies**		
Rabbit anti‐VIP	Abcam	Cat. #ab272726
Mouse anti‐PKC‐δ	BD Biosciences	Cat. #610398; RRID: AB_397781
Guinea‐pig anti‐TH	Synaptic system	Cat. #213104; RRID:AB_2619897
Mouse anti‐TH	Milipore	Cat. #MAB318; RRID: AB_2201528
Chicken anti‐GFP	Aves Lab	Cat. #GFP‐1020; RRID: AB_10000240
Rat anti‐RFP	Chromotek	Cat. #5f8; RRID: AB_2336064
Rabbit anti‐2A peptide	Sigma‐Aldrich	Cat. #ABS31; RRID: AB_11214282
Goat anti‐guinea‐pig A568	Life technologies	Cat. #A11075; RRID: AB_141954
Goat anti‐mouse A488	Invitrogene	Cat. #A11001; RRID: AB_2534069
Goat anti‐chicken A488	Invitrogene	Cat. #A11039; RRID: AB_2534096
Goat anti‐rabbit A488	Invitrogene	Cat. #A11008; RRID: AB_143165
Goat anti‐rat A568	Invitrogene	Cat. #A11077; RRID: AB_2534121
Goat anti‐guinea‐pig A568	Life technologies	Cat. #A11075; RRID: AB_141954
Goat anti‐mouse A647	Life technologies	Cat. #A21236; RRID: AB_2535805
Goat anti‐rabbit A647	Life technologies	Cat. #A21245; RRID: AB_2535813
**Viral vectors**		
AAV1‐EF1a‐FLEX‐synaptophysine	IMN VectorCore	
AAV5‐EF1a‐DIO‐EYFP	Addgene	Cat. #v147551
pSADB19dG‐mCherry	Viral Core Facility, Charité ‐ Universitätsmedizin Berlin	Cat. #RV03
pSADB19dG‐GFP	Viral Core Facility, Charité ‐ Universitätsmedizin Berlin	Cat. #RV03
AAV1‐EF1a‐DIO‐TVA950‐T2A‐WPRE	IMN VectorCore	Cat#PV351
AAV1‐EF1a‐DIO‐TVA950‐T2A‐cvs11G‐WPRE	IMN VectorCore	
AAV5‐pAAV‐flex‐taCasp3‐TEVp	Addgene	v143243// 45580‐AAV5
AAV1‐EF1α‐DIO‐jGCaMP7f	VVF ETH Zurich	cat. #v398‐1; Addgene #104483
AAV1‐EF1a‐DIO‐hM3D(Gq)‐mCherry	VVF ETH Zurich	cat. #v98‐1; Addgene (Plasmid #50460)
AAV1‐EF1a‐DIO‐mCherry	VVF ETH Zurich	v114‐1; Addgene (Plasmid #50462)
AAV5‐EF1a‐DIO‐eNpHR3.0‐mCherry	UNC Vector Core	
**Chemicals**		
Streptavidin A568	Life technologies	Cat#S11226, RRID: AB_2315774
Normal goat serum	Sigma‐Aldrich	S26‐100ML
Hoechst	Invitrogene	RRID: AB_2651133
DAPI	Invitrogene	Cat. D1306, RRID:AB_2629482
DNQX disodium salt	Tocris Bioscience	Cat. #2312
D‐AP5	Tocris Bioscience	Cat. #0106
TTX	Tocris Bioscience	Cat. #1078
4‐AP	Ascent	Ref: Asc‐122
Pontamine sky blue	Sigma‐Aldrich	Cat. #C8679
Euthasol	centravet	
Meloxicam	centravet	
Lurocaine	centravet	
Buprenorphine	virbac	
Isoflurane	virbac	
Clozapine N‐oxyde	Tocris Bioscience	Cat. #4936
**Experimental models: Organisms/strains**		
C57BL/6JRj	Janvier Labs	SC‐C57J‐F and M
Vip‐IRES‐Cre	On site production (PIV‐EXPE)	RRID:IMSR_JAX:010908; Strain #:010908
Vip‐IRES‐Cre/COP4	On site production (PIV‐EXPE)	COP4: RRID:IMSR_JAX:024109; Strain #:024109
**Software and algorithms**		
Spike2	Cambridge Electronic Design Limited	RRID: SCR_000903
ImageJ		RRID:SCR_003070
Fiji		RRID: SCR_002285
Qupath v5.0	Bankhead, P. et al. 2017	RRID: SCR_018257
Abba	BioImaging & Optics Platform, EPFL	Atlas V3p1; RRID: SCR_023857
Imaris	Oxford Instruments	RRID: SCR_007370
Ethovision XT v17.0	Noldus	RRID: SCR_004074
Deeplabcut	Deeplabcut	RRID: SCR_021391
Graphpad Prism	Graphpad Prism	RRID: SCR_002798
NDP.view2	Hamamatsu	RRID: SCR_025177
Inkscape	Inkscape	RRID: SCR_014479
**Imaging systems**		
Epifluorescent microscope	Olympus BX63	
Confocal microscope	Leica TCS SP5	
Digital Slide Scanner	Hamamatsu Nanozoomer 2.0HT	
UltraMicroscope II	Biotech‐Milteniy	

### 4.23 Quantification and Statistical Analysis

We performed the statistical analysis using GraphPad Prism 5 (GraphPad, LaJolla, CA). Sample size (n) generally represents the number of experimental replicates, as indicated in the figure legends. In all behavioral experiments, (n) refers to the number of mice used, while in slice recording experiments, (n) denotes the number of recorded cells. Data are presented as mean ± SEM in all figures. For two‐group comparisons, statistical significance was determined by two‐tailed paired or unpaired Student's t‐tests or non‐parametric analogs, when assumptions for parametric testing were not satisfied. Normality was tested using the D'Agostino and Pearson omnibus normality test. For multiple group comparisons, one‐way analysis of variance (ANOVA) tests were used for normally distributed data, followed by post hoc analyses. For data that were not normally distributed, non‐parametric tests for the appropriate group types were used instead, such as Mann–Whitney. Exact p‐values and the corresponding statistical methods are provided in the figure captions and legends. A detailed description of the statistical tests can be found in Table 2.

**TABLE 2:** Summary of statistical analyses.

Figure	Experiment	Compared groups	Measured variable (graph title)	Statistic test	Source of variation	p‐value
Figure 3	Ex vivo electrophysiology	BNST vs. CeL neurons	1st peak amplitude (pA)	Mann–Whitney		0.3193
Figure 3	Ex vivo electrophysiology	BNST vs. CeL neurons	Paired Pulse Ratio (PPR)	Mann–Whitney		**0.0361**
Figure 3	Ex vivo electrophysiology	BNST vs. CeL neurons	Latency (ms)	Mann–Whitney		0.6063
Figure 3	Ex vivo electrophysiology	BNST vs. CeL neurons	Decay time (ms)	Mann–Whitney		0.1060
Figure 3	Ex vivo electrophysiology	BNST vs. CeL neurons	Rise time (ms)	Mann–Whitney		0.8438
Figure 3	Ex vivo electrophysiology	Ctrl vs. AP5‐CNQX	1st peak amplitude (pA)	Paired T‐test		**0.0050**
Figure 3	Ex vivo electrophysiology	Ctrl vs. TTX vs. TTX+4A‐P	1st peak amplitude (pA)	N too small		
Figure 4C	Histological quantification	Ctrl vs. Casp3	VIP+ neurons DRN	Mann–Whitney		**<0.0001**
Figure 4C	Histological quantification	Ctrl vs. Casp3	TH+ neurons DRN	Unpaired T‐test		0.0753
Figure 4D	Histological quantification	Ctrl vs. Casp3	VIP+ fibers ovBNST	Mann–Whitney		**<0.0001**
Figure 4D	Histological quantification	Ctrl vs. Casp3	TH+ fibres ovBNST	Mann–Whitney		**<0.0001**
Figure 4E	Histological quantification	Ctrl vs. Casp3	VIP+ fibers CeA	Mann–Whitney		**<0.0001**
Figure 4E	Histological quantification	Ctrl vs. Casp3	TH+ fibres CeA	Mann–Whitney		**<0.0001**
Figure 5E	Sleep	Ctrl vs. Casp3	NREM time active (%)	Unpaired T‐test		0.4375
Figure 5F	Sleep	Ctrl vs. Casp3	NREM time inactive (%)	Unpaired T‐test		0.7605
Figure 5H	Sleep	Ctrl vs. Casp3	NREM bouts active (n)	Unpaired T‐test		**0.0207**
Figure 5I	Sleep	Ctrl vs. Casp3	NREM bouts duration active	Unpaired T‐test		**0.0038**
Figure 5J	Sleep	Ctrl vs. Casp3	NREM bouts inactive (n)	Unpaired T‐test		0.3486
Figure 5K	Sleep	Ctrl vs. Casp3	NREM bouts duration inactive	Unpaired T‐test		0.3050
Figure 5L	Sleep	Ctrl vs. Casp3	Spindles density active	Unpaired T‐test		0.2357
Figure 5M	Sleep	Ctrl vs. Casp3	Spindles duration active	Unpaired T‐test		**0.0118**
Figure 5N	Sleep	Ctrl vs. Casp3	Spindles density inactive	Unpaired T‐test		0.5951
Figure 5O	Sleep	Ctrl vs. Casp3	Spindles duration inactive	Unpaired T‐test		0.6303
Figure 6C	OFT	Ctrl vs. Casp3	Time spent in center (%)	Unpaired T‐test		0.3591
Figure 6C	OFT	Ctrl vs. Casp3	Distance moved (cm)	Unpaired T‐test		0.4645
Figure 6D	EPM	Ctrl vs. Casp3	OA/(OA+CA) *100 (%)	Unpaired T‐test		**0.0267**
Figure 6D	EPM	Ctrl vs. Casp3	Protected Headips (%)	Unpaired T‐test		**0.0396**
Figure 6D	EPM	Ctrl vs. Casp3	Protected SAP (%)	Mann–Whitney		0.2841
Figure 6D	EPM	Ctrl vs. Casp3	Time spent in center (%)	Unpaired T‐test		0.8845
Figure 6D	EPM	Ctrl vs. Casp3	Nb of entries in CA	Unpaired T‐test		0.7502
Figure 6D	EPM	Ctrl vs. Casp3	Distance moved (cm)	Unpaired T‐test		0.3290
Figure 6E	L/D box	Ctrl vs. Casp3	Time in light (%)	Unpaired T‐test		0.9668
Figure 6E	L/D box	Ctrl vs. Casp3	Nb of entries in light	Unpaired T‐test		0.7103
Figure 6E	L/D box	Ctrl vs. Casp3	Latency to light (s)	Unpaired T‐test		0.2553
Figure 6E	L/D box	Ctrl vs. Casp3	Distance moved in light (cm)	Unpaired T‐test		0.9202
Figure 6E	L/D box	Ctrl vs. Casp3	Nb of nose pokes	Unpaired T‐test		0.8581
Figure 7D	VLT	Ctrl vs. Casp3 (Group) and Days	Escape probability along days	Mixed‐effects analysis	F_Group_ (1, 32) = 10.20	**0.0031**
Figure 7D	VLT	Female vs. Male (Sex) and Days	Escape probability along days	Mixed‐effects analysis	F_Sex_ (1, 16) = 2.407	0.1404
Figure 7D	VLT	Ctrl vs. Casp3	Escape probability Day1	Mann–Whitney		**0.0013**
Figure 7D	VLT	Ctrl vs. Casp3	Escape probability Day2	Mann–Whitney		**0.0423**
Figure 7D	VLT	Ctrl vs. Casp3	Escape probability Day7	Mann–Whitney		**0.0034**
Figure 7E	VLT	Ctrl vs. Casp3	Pie charts percentage escaping/no escaping Day1	Fisher's exact test (Contingency)		**<0.0001**
Figure 7F	VLT	Ctrl vs. Casp3	Max velocity during cue Day1 (cm/s)	Mann–Whitney		**0.0056**
Figure 7G	VLT	Ctrl vs. Casp3 (Group) and Days	Latency 1st escape along days	Mixed‐effects analysis	F_Days_ (1.928, 53,03) = 7.372	**0.0017**
Figure 7H	VLT	Ctrl vs. Casp3 (Group) and Days	Time in shelter along days (%)	2Way RM ANOVA	F_Group_ (1, 32) = 5.343, F_Days_ (1.892, 60,53) = 3.747	**Group: 0.0274, Days: 0.0314**
Figure 7H	VLT	Ctrl vs. Casp3 (Group) and Days	Time in trigger zone along days (%)	2Way RM ANOVA	F_Group_ (1, 32) = 4.616	**0.0393**
Figure 7I	VLT	Ctrl vs. Casp3	Time in trigger zone H2 (%)	Mann–Whitney		0.4174
Figure 7J	VLT	Ctrl vs. Casp3	Nb of rearing H1	Mann–Whitney		0.4375
Figure 7J	VLT	Ctrl vs. Casp3	Rearing (% from H1) Before cue	Unpaired T‐test; One sample t test		**0.0062;** 0.3974 (Ctrl)**, 0,0003 (Casp3)**
Figure 7J	VLT	Ctrl vs. Casp3	Rearing (% from H1) After cue	Mann–Whitney; One sample t test		**0.0046; 0.0013 (Ctrl),** 0.6910 (Casp3)
Figure 7K	VLT	Ctrl vs. Casp3	Cumulated immobility Day1 (s)	Unpaired T‐test		**0.0135**
Figure 7L	VLT	Ctrl vs. Casp3	Distance moved H1 (cm)	Mann–Whitney		0.6212
Figure 8A	Z‐Score NREM (active)	Ctrl vs. Casp3	Z‐Score NREM active	Unpaired T‐test		**0.0012**
Figure 8B	Z‐Score NREM (inactive)	Ctrl vs. Casp3	Z‐Score NREM inactive	Unpaired T‐test		0.7542
Figure 8C	Z‐Score Risk assessment	Ctrl vs. Casp3	Z‐Score Risk assessment	Unpaired T‐test		**0.0075**
Figure 8D	Z‐Score Defensive Behavior	Ctrl vs. Casp3	Z‐Score Defensive Behavior	Mann–Whitney		**0.0009**
Figure 8E	Z‐Score Anxiety	Ctrl vs. Casp3	Z‐Score Anxiety	Mann–Whitney		0.4654
Figure 8F	Z‐Score Locomotion	Ctrl vs. Casp3	Z‐Score Locomotion	Mann–Whitney		0.5334
Supplementary Figure 3B	Brain clearing	AAV5‐EF1‐DIO‐eYFP injected brains (DRN)	Mean grey value in DRN	Brown‐Forsythe ANOVA test		0.9579
Supplementary Figure 6B	Sleep	Ctrl vs. Casp3	AWAKE time active (%)	Unpaired T‐test		0.4488
Supplementary Figure 6C	Sleep	Ctrl vs. Casp3	AWAKE time inactive (%)	Unpaired T‐test		0.7368
Supplementary Figure 6D	Sleep	Ctrl vs. Casp3	AWAKE bouts active (n)	Unpaired T‐test		**0.0232**
Supplementary Figure 6E	Sleep	Ctrl vs. Casp3	AWAKE bouts duration active	Unpaired T‐test		0.1809
Supplementary Figure 6F	Sleep	Ctrl vs. Casp3	AWAKE bouts inactive (n)	Unpaired T‐test		0.4889
Supplementary Figure 6G	Sleep	Ctrl vs. Casp3	AWAKE bouts duration inactive	Mann–Whitney		0.1949
Supplementary Figure 6B	Sleep	Ctrl vs. Casp3	REM time active (%)	Unpaired T‐test		0.6160
Supplementary Figure 6C	Sleep	Ctrl vs. Casp3	REM time inactive (%)	Unpaired T‐test		0.6850
Supplementary Figure 6D	Sleep	Ctrl vs. Casp3	REM bouts active (n)	Unpaired T‐test		0.3492
Supplementary Figure 6E	Sleep	Ctrl vs. Casp3	REM bouts duration active	Unpaired T‐test		0.0521
Supplementary Figure 6F	Sleep	Ctrl vs. Casp3	REM bouts inactive (n)	Unpaired T‐test		0.2228
Supplementary Figure 6G	Sleep	Ctrl vs. Casp3	REM bouts duration inactive	Unpaired T‐test		0.5133
Supplementary figure 7A	Histological quantification	Female vs. Male and Ctrl vs. Casp3	VIP+ neurons DRN	2Way ANOVA and Uncorrected Fisher's LSD	F_Interaction_ (1, 34) = 10.27, F_Group_ (1, 34) = 42.24, F_sex_ (1, 34) = 8.323	**Interaction: 0.0029, Group: <0.0001, Sex: 0.0067 Female Ctrl vs. Casp3: 0.0259, Male Ctrl vs. Casp3: <0.0001, Ctrl Female vs. Male: 0.0001**
Supplementary figure 7B	Histological quantification	Female vs. Male and Ctrl vs. Casp3	TH+ neurons DRN	2Way ANOVA and Uncorrected Fisher's LSD	F_Interaction_ (1, 34) = 7.668, F_Group_ (1, 34) = 4.765, F_Sex_ (1, 34) = 4.497	**Interaction: 0.0090, Group: 0.0360, Sex: 0.0413 Male Ctrl vs. Casp3: 0.0013, Ctrl Female vs. Male: 0.0015**
Supplementary figure 7 C	Histological quantification	Female vs. Male and Ctrl vs. Casp3	VIP+ fibers ovBNST	2Way ANOVA	F_Interaction_ (1, 33) = 0.9810, F_Group_ (1,33) = 91.72, F_Sex_ (1,33) = 0.1646	Interaction: 0.3292, **Group: < 0.0001,** Sex: 0.6876
Supplementary figure 7 D	Histological quantification	Female vs. Male and Ctrl vs. Casp3	TH+ fibres ovBNST	2Way ANOVA	F_Interaction_ (1, 33) = 0.8754, F_Group_ (1,33) = 53.66, F_Sex_ (1,33) = 0.5440	Interaction: 0.3563, **Group: < 0.0001,** Sex: 0.4660
Supplementary figure 7 E	Histological quantification	Female vs. Male and Ctrl vs. Casp3	VIP+ fibers CeA	Mann–Whitney	F_Interaction_ (1, 33) = 0.7421, F_Group_ (1,33) = 73.37, F_Sex_ (1,33) = 0.01625	Interaction: 0.3952, **Group: < 0.0001,** Sex: 0.8993
Supplementary figure 7 F	Histological quantification	Female vs. Male and Ctrl vs. Casp3	TH+ fibres CeA	Mann–Whitney	F_Interaction _(1, 33) = 0.9287, F_Group_ (1,33) = 50.01, FSex (1,33) = 0.7193	Interaction: 0.3422, **Group: < 0.0001,** Sex: 0.7193
Supplementary figure 8A	OFT	Female vs. Male and Ctrl vs. Casp3	Time spent in center (%)	2Way ANOVA	F_Interaction_ (1, 33) = 1.764, F_Group_(1, 33) = 1.560, F_Sex_ (1, 33) = 0.2575	Interaction: 0.1933, Group: 0.2205, Sex: 0.6152
Supplementary figure 8B	OFT	Female vs. Male and Ctrl vs. Casp3	Distance moved (cm)	2Way ANOVA	F_Interaction_ (1, 33) = 2.496, F_Group_(1, 33) = 1.306, F_Sex_ (1, 33) = 0.4129	Interaction: 0.1237, Group: 0.2614, Sex: 0.5249
Supplementary figure 8C	EPM	Female vs. Male and Ctrl vs. Casp3	OA/(OA+CA) *100 (%)	2Way ANOVA	F_Interaction_ (1, 33) = 0.7137, F_Group_(1, 33) = 4.769, F_Sex_ (1, 33) = 1.688	Interaction: 0.4043, **Group: 0.0362**, Sex: 0.2028
Supplementary figure 8D	EPM	Female vs. Male and Ctrl vs. Casp3	Protected Headips (%)	2Way ANOVA	F_Interaction_ (1, 33) = 0.01261, F_Group_ (1, 33) = 4.075, F_Sex_ (1, 33) = 3.577	Interaction: 0.09113, Group: 0.0517, Sex: 0.0674
Supplementary figure 8E	EPM	Female vs. Male and Ctrl vs. Casp3	Protected SAP (%)	2Way ANOVA	F_Interaction_ (1, 33) = 0.8405, F_Group_ (1, 33) = 2.035, F_Sex_ (1, 33) = 1.007	Interaction: 0.03659, Group: 0.1631, Sex: 0.3228
Supplementary figure 8F	EPM	Female vs. Male and Ctrl vs. Casp3	Time spent in center (%)	2Way ANOVA	F_Interaction_ (1, 33) = 1.308, F_Group_ (1, 33) = 0.0008212, F_Sex_ (1, 33) = 0.5989	Interaction: 0.02610, Group: 0.9773, Sex: 0.4445
Supplementary figure 8G	EPM	Female vs. Male and Ctrl vs. Casp3	Nb of entries in CA	2Way ANOVA	F_Interaction_ (1, 33) = 0.04299, F_Group_ (1, 33) = 0.1104, F_Sex_ (1, 33) = 0.1104	Interaction: 0.8370, Group: 0.7417, Sex: 0.7417
Supplementary figure 8H	EPM	Female vs. Male and Ctrl vs. Casp3	Distance moved (cm)	2Way ANOVA	F_Interaction_ (1, 33) = 0.03415, F_Group_ (1, 33) = 0.7495, F_Sex_ (1, 33) = 0.1709	Interaction: 0.8545, Group: 0.3929, Sex: 0.1709
Supplementary figure 8I	L/D box	Female vs. Male and Ctrl vs. Casp3	Time in light (%)	2Way ANOVA	F_Interaction_ (1, 34) = 0.07283, F_Group_ (1, 34) = 0.01491, F_Sex_ (1,34) = 10.26	Interaction: 0.7889, Group: 0.9035, **Sex: 0.0029**
Supplementary figure 8J	L/D box	Female vs. Male and Ctrl vs. Casp3	Nb of entries in light	2Way ANOVA	F_Interaction_ (1, 34) = 0.4018, F_Group_ (1, 34) = 0.1364, F_Sex_ (1,34) = 0.005135	Interaction: 0.5304, Group: 0.7142, Sex: 0.9433
Supplementary figure 8K	L/D box	Female vs. Male and Ctrl vs. Casp3	Latency to light (s)	2Way ANOVA	F_Interaction_ (1, 34) = 0.2905, F_Group_ (1, 34) = 1.180, F_Sex_ (1,34) = 1.568	Interaction: 0.5934, Group: 0.2850, Sex: 0.2191
Supplementary figure 8L	L/D box	Female vs. Male and Ctrl vs. Casp3	Distance moved in light (cm)	2Way ANOVA	F_Interaction_ (1, 34) = 0.003916, F_Group_ (1, 34) = 0.05925, F_Sex_ (1,34) = 0.1614	Group: 0.9391, Sex: 0.6904
Supplementary figure 8M	L/D box	Female vs. Male and Ctrl vs. Casp3	Nb of nose pokes	2Way ANOVA and Uncorrected Fisher's LSD	F_Interaction _(1, 34) = 7.798, F_Group_ (1, 34) = 0.1519, F_Sex_ (1,34) = 10.75	**Interaction: 0.0085**, Group: 0.6991, **Sex: 0.0024 Male Ctrl vs. Casp3: 0.0310, Ctrl Female vs. Male: 0.0001**
Supplementary figure 8N	VLT	Female vs. Male and Ctrl vs. Casp3	Escape probability Day1	2Way ANOVA	F_Interaction _(1, 30)= 0.06601, F_Group_ (1,30) = 10.36, F_Sex_ (1,30) = 1.844	Interaction: 0.7990, **Group: 0.0031,** Sex: 0.1846
Supplementary figure 8O	VLT	Female vs. Male and Ctrl vs. Casp3	Escape probability Day2	2Way ANOVA	F_Interaction _(1, 30)= 0.5643, F_Group_ (1,30) = 4.199, F_Sex_ (1,30) = 2.165	Interaction: 0.4584, **Group: 0.0493,** Sex: 0.1516
Supplementary figure 8P	VLT	Female vs. Male and Ctrl vs. Casp3	Escape probability Day7	2Way ANOVA	F_Interaction_ (1, 30)= 0.8769, F_Group_ (1,30) = 10.55, F_Sex_ (1,30) = 2.511	Interaction: 0.3565, **Group: 0.0029,** Sex: 0.1235
Supplementary figure 8Q	VLT	Female vs. Male and Ctrl vs. Casp3	Max velocity during cue Day1 (cm/s)	2Way ANOVA	F_Interaction _(1, 30)= 0.1355, F_Group_ (1,30) = 7.728, F_Sex_ (1,30) = 2.938	Interaction: 0.7154, **Group: 0.0093,** Sex: 0.0974
Supplementary figure 8R	VLT	Female vs. Male and Ctrl vs. Casp3	Time in trigger zone H2 (%)	2Way ANOVA	F_Interaction _(1, 30)= 0.2398, F_Group_ (1,30) = 0.01005, F_Sex_ (1,30) = 1.667	Interaction: 0.6279, Group: 0.9208, Sex: 0.2065
Supplementary figure 8S	VLT	Female vs. Male and Ctrl vs. Casp3	Nb of rearing H1	2Way ANOVA	F_Interaction _(1, 30)= 1.389, F_Group_ (1,30) = 2.174, F_Sex_ (1,30) = 3.942	Interaction: 0.2479, Group: 0.1508, Sex: 0.0563
Supplementary figure 8T	VLT	Female vs. Male and Ctrl vs. Casp3	Rearing (% from H1) Before cue	2Way ANOVA	F_Interaction_ (1, 30)= 5.633e‐6, F_Group_ (1,30) = 7.310, F_Sex_ (1,30) =2.306	Interaction: 0.9981, **Group: 0.0112**, Sex: 0.1393
Supplementary figure 8U	VLT	Female vs. Male and Ctrl vs. Casp3	Rearing (% from H1) After cue	2Way ANOVA	F_Interaction_ (1, 30)= 0.003867, F_Group_ (1,30) = 5.035, F_Sex_ (1,30) = 3.889	Interaction: 0.9508, **Group: 0.0324**, Sex: 0.0579
Supplementary figure 8V	VLT	Female vs. Male and Ctrl vs. Casp3	Cumulated immobility Day1 (s)	2Way ANOVA	F_Interaction_ (1, 30)= 0.3944, F_Group_ (1,30) = 5.394, F_Sex_ (1,30) = 2.508	Interaction: 0.5347, **Group: 0.0272**, Sex: 0.1237
Supplementary figure 8W	VLT	Female vs. Male and Ctrl vs. Casp3	Distance moved H1 (cm)	2Way ANOVA	F_Interaction _(1, 30)= 0.7164, F_Group_ (1,30) = 0.2803, F_Sex_ (1,30) = 1.830	Interaction: 0.4040, Group: 0.6004, Sex: 0.1862
Supplementary figure 9E	VLT	Ctrl vs. eNpHR	Escape probability along days	Mixed‐effects analysis	F_Interaction_ (1.965, 31.45) = 4.098, F_Group_ (1, 18) = 0.1843, F_Days_ (1.965, 31.45) = 6.566	**Interaction: 0.0268**, Group: 0.6728, **Days: 0.0043 eNpHR (D1 vs. D2 and D2 vs. D7): 0.0239 and 0.0307**
Supplementary figure 9E	VLT	Ctrl vs. eNpHR	Escape probability Day 1	Unpaired T‐test, One‐tailed		**0.0463**
Supplementary figure 9E	VLT	Ctrl vs. eNpHR	Escape probability Day 2	Unpaired T‐test, One‐tailed		0.1415
Supplementary figure 9E	VLT	Ctrl vs. eNpHR	Escape probability Day 7	Mann–Whitney, One ‐tailed		0.9620
Supplementary figure 9F	VLT	Ctrl vs. eNpHR	Max velocity during cue Day1 (cm/s)	Unpaired T‐test, One‐tailed		0.3527
Supplementary figure 9G	VLT	Ctrl vs. eNpHR	Pie charts percentage escaping/no escaping Day1	Fisher's exact test (Contingency), one‐sided		**0.0007**
Supplementary figure 9H	VLT	Ctrl vs. eNpHR	Latency 1st escape along days	Mixed‐effects analysis	F_Interaction_ (1.978, 14.84) = 0.1471, F_Group_ (1, 11) = 0.005027, F_Days_ (1.978, 14.84) = 2.990	Interaction: 0.8624, Group: 0.9447, Days: 0.0815
Supplementary figure 9I	VLT	Ctrl vs. eNpHR	Time in shelter along days (%)	2Way RM ANOVA	F_Interaction_ (1.366, 25.85) = 0.05529, F_Group_ (1, 16) = 3.313, F_Days_ (1.366, 25.85) = 1.650	Interaction: 0.8852, Group: 0.0875, Days: 0.2164
Supplementary figure 9I	VLT	Ctrl vs. eNpHR	Time in trigger zone along days (%)	2Way RM ANOVA	F_Interaction_ (1.290, 20.64) = 0.7379, F_Group_ (1, 16) = 2.133, F_Days_ (1.290, 20.64) = 1.735	Interaction: 0.4337, Group: 0.1635, Days: 0.2043
Supplementary figure 9J	VLT	Ctrl vs. eNpHR	Time in trigger zone H2 (%)	Mann–Whitney		0.2863
Supplementary figure 9K	VLT	Ctrl vs. eNpHR	Cumulated immobility Day1 (s)	Mann–Whitney, One ‐tailed		0.4317
Supplementary figure 9L	VLT	Ctrl vs. eNpHR	Distance moved H1 (cm)	Mann–Whitney		0.3401
Supplementary figure 10D	OFT	Ctrl vs. hM3D(Gq)	Time spent in center (%)	Mann–Whitney		0.5573
Supplementary figure 10D	OFT	Ctrl vs. hM3D(Gq)	Distance moved (cm)	Unpaired T‐test		0.3253
Supplementary figure 10E	EPM	Ctrl vs. hM3D(Gq)	OA/(OA+CA) *100 (%)	Unpaired T‐test		0.0513
Supplementary figure 10E	EPM	Ctrl vs. hM3D(Gq)	Protected Headips (%)	Mann–Whitney		**0.0034**
Supplementary figure 10E	EPM	Ctrl vs. hM3D(Gq)	Protected SAP (%)	Mann–Whitney		0.4696
Supplementary figure 10E	EPM	Ctrl vs. hM3D(Gq)	Time spent in center (%)	Unpaired T‐test		0.6294
Supplementary figure 10E	EPM	Ctrl vs. hM3D(Gq)	Nb of entries in CA	Unpaired T‐test		0.5662
Supplementary figure 10E	EPM	Ctrl vs. hM3D(Gq)	Distance moved (cm)	Unpaired T‐test		0.1753
Supplementary figure 10F	L/D box	Ctrl vs. hM3D(Gq)	Time in light (%)	Unpaired T‐test		0.6877
Supplementary figure 10F	L/D box	Ctrl vs. hM3D(Gq)	Nb of entries in light	Mann–Whitney		0.3016
Supplementary figure 10F	L/D box	Ctrl vs. hM3D(Gq)	Latency to light (s)	Mann–Whitney		0.6398
Supplementary figure 10F	L/D box	Ctrl vs. hM3D(Gq)	Distance moved in light (cm)	Unpaired T‐test		0.6689
Supplementary figure 10F	L/D box	Ctrl vs. hM3D(Gq)	Nb of nose pokes	Unpaired T‐test		0.4775
Supplementary figure 10H	VLT	Ctrl vs. hM3D(Gq) Groups and Days	Escape probability along days	Mixed‐effects analysis	F_Interaction_ (1.635, 49.87) = 6.901, F_Group_ (1, 32) = 1.405, F_Days_ (1.635, 49.87) = 23.06	**Interaction: <0.0001,** Group: 0,2447, **Days: 0.004 Day 1 (Ctrl vs. hM3D(Gq)): 0.0059, Ctrl (D2 vs. D7): 0,0009, hM3D(Gq) (D1 vs. D7 and D2 vs. D7): 0,0006 and <0.0001**
Supplementary figure 10H	VLT	Ctrl vs. hM3D(Gq)	Escape probability Day 1	Mann–Whitney, One ‐tailed		**0.0355**
Supplementary figure 10H	VLT	Ctrl vs. hM3D(Gq)	Escape probability Day 2	Mann–Whitney, One ‐tailed		0.2675
Supplementary figure 10H	VLT	Ctrl vs. hM3D(Gq)	Escape probability Day 3	Mann–Whitney, One ‐tailed		0.2648
Supplementary figure 10I	VLT	Ctrl vs. hM3D(Gq)	Pie charts percentage escaping/no escaping Day1	Fisher's exact test (Contingency), one‐sided		**<0.0001**
Supplementary figure 10J	VLT	Ctrl vs. hM3D(Gq) Groups and Days	Latency 1st escape along days	Mixed‐effects analysis	F_Interaction_ (1.698, 40.74) = 6.115, F_Group_ (1, 30) = 0.3459, F_Days_ (1.698, 40.74) = 0.09847	**Interaction: 0.0069**, Group: 0.5608, Days: 0.8797 **Day 1 (Ctrl vs. hM3D(Gq)): 0.016**
Supplementary figure 10K	VLT	Ctrl vs. hM3D(Gq)	Max velocity during cue Day1 (cm/s)	Unpaired T‐test, one tailed		0.1662
Supplementary figure 10L	VLT	Ctrl vs. hM3D(Gq)	Time in shelter along days (%)	Mixed‐effects analysis	F_Interaction_ (1.565, 47.73) = 3.175, F_Group_ (1,32) = 0.04954, F_days_ (1.565, 47.73) = 7.237	Interaction: 0.0623, Group: 0.8253, **Days: 0.0036**
Supplementary figure 10L	VLT	Ctrl vs. hM3D(Gq)	Time in trigger zone along days (%)	Mixed‐effects analysis	F_Interaction_ (1.468, 44.78) = 1.974, F_Group_ (1,32) = 0.02397, F_days_ (1.468, 44.78) = 7.744	Interaction: 0.1609, Group: 0.8779, **Days: 0.0032**
Supplementary figure 10M	VLT	Ctrl vs. hM3D(Gq)	Time in trigger zone H2 (%)	Unpaired T‐test		0.4847
Supplementary figure 10N	VLT	Ctrl vs. hM3D(Gq)	Cumulated immobility Day1 (s)	Unpaired T‐test, one tailed		0.1376
Supplementary figure 10O	VLT	Ctrl vs. hM3D(Gq)	Distance moved H1 (cm)	Unpaired T‐test		0.9155
Supplementary Figure 11	In vivo electrophysiology	Ctrl vs. Casp3	Firing rate (BNST)	Mann–Whitney		**0.0045**
Supplementary Figure 11	In vivo electrophysiology	Ctrl vs. Casp3	Firing rate (CeA)	Mann–Whitney		**0.0227**

### 4.24 Animals and Ethics Approval

All animal procedures were conducted in accordance with the European directive 2010‐63‐EU and with approval from the Bordeaux University Animal Care and Use Committee (license authorization 21134). All efforts were made to minimize animal suffering and reduce the number of animals used. Non‐human primate experiments were performed on tissue from a previously published brain bank [79, 80, 81]. Experiments were carried out in accordance with the European Communities Council Directive of November 24, 1986 (86/609/EEC) regarding care of laboratory animals in an AAALAC‐accredited facility following acceptance of the study design by the Institute of Lab Animal Science (Chinese Academy of Science, Beijing, China) IACUC. All procedures adhered to the 3Rs principles (Replacement, Reduction, and Refinement) to ensure ethical and humane research practices.

## Author Contributions

Conceptualization: A.G.; C.G. G.F. and F.G.; Investigation: A.G., F.G; E.P, S.D, S.D.; N.B.; E.L.; L.B.; D.D.C.M.; Resources: F.G., J.B., M.L. and E.B; Writing – Original Draft: A.G. and F.G.; Visualization: A.G., T.D., E.P.; Supervision: F.G.; Funding Acquisition: F.G., J.B. and G.F.

## Conflicts of Interest

The authors declare no conflicts of interest.

## Supporting information




**Supporting File**: advs73959‐sup‐0001‐SuppMat.docx.

## Data Availability

The data that support the findings of this study are available from the corresponding author upon reasonable request.
