# Supplementary material for: Dorsal Raphe VIP Neurons Are Critical for Survival‐Oriented Vigilance
Source: Adv Sci (Weinh). 2026 Jan 25;13(18):e23809. doi: 10.1002/advs.202523809 (PMC13042892; doi:10.1002/advs.202523809)
Supplement: Supplementary file 1 — Supporting File: advs73959‐sup‐0001‐SuppMat.docx. [file ADVS-13-e23809-s001.docx]

**Dorsal raphe VIP neurons are critical for survival-oriented vigilance.**

Adriane GUILLAUMIN^1^, Emma PERROT^1^, Thibault DHELLEMMES^1^, Laura BOI^2^, Daniel DE CASTRO MEDEIROS^2^, Christelle GLANGETAS^1^, S. DUMAS^3^, Sandra DOVERO^1^, Nathalie BIENDON^1^, Elodie LADEVEZE^1^, Maëlle HARDEL^1^, Marc LANDRY^1^, Erwan BEZARD^1^, Jérôme BAUFRETON^1^, Gilberto FISONE^2^ and François GEORGES^1*^

^1^ Université de Bordeaux, CNRS, IMN, UMR5293 F-33000 Bordeaux, France

^2^ Department of Neuroscience, Karolinska Institutet, 17177 Stockholm, Sweden

^3^ ORAMACELL, 75006 Paris, France

***Corresponding author:**

[francois.georges@u](mailto:francois.georges@u)-bordeaux.fr

# SUPPLEMENTAL INFORMATION TITLES AND LEGENDS


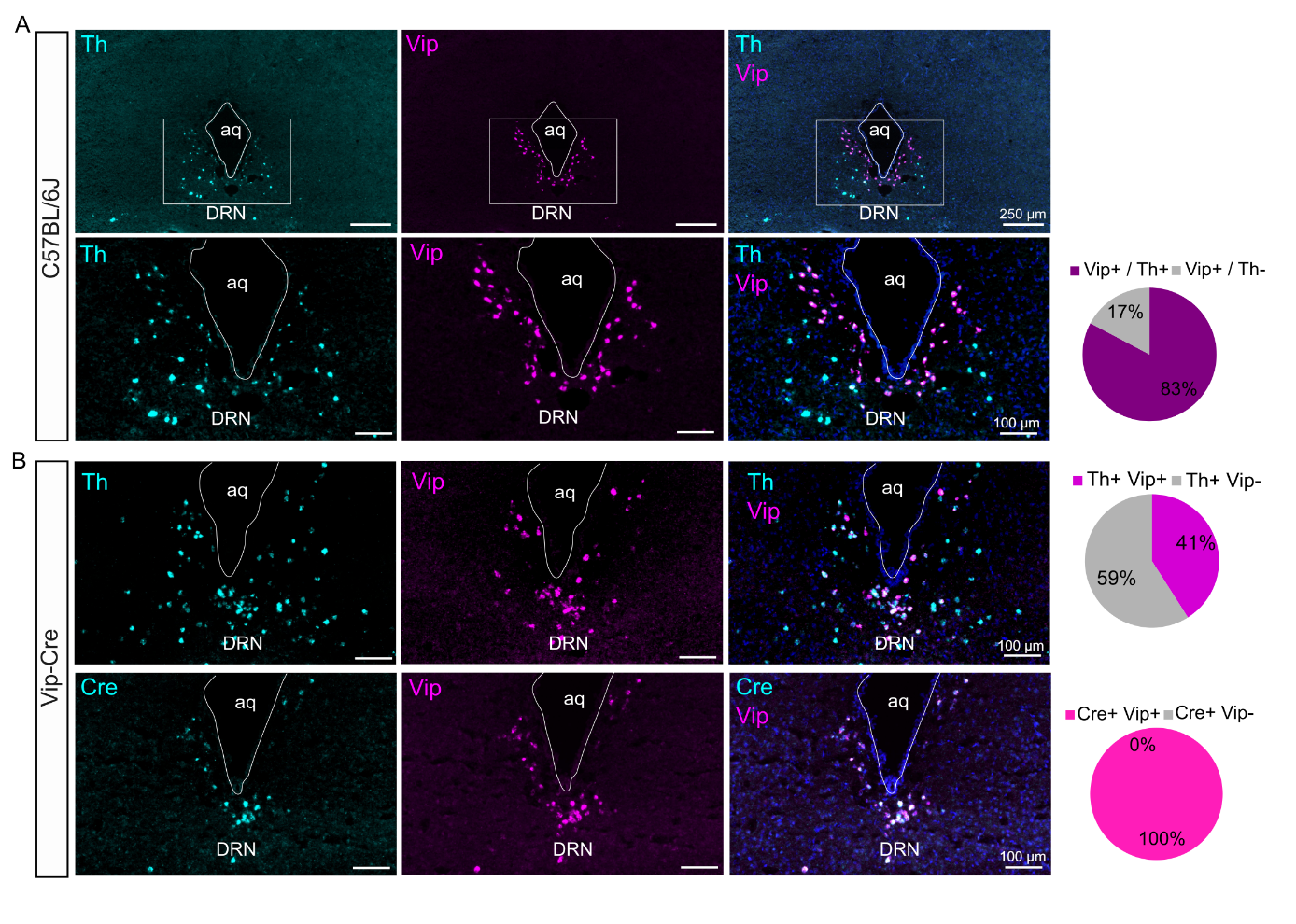


**Supplementary Figure 1. *In situ* hybridization of Vip and Th mRNA in the DRN in wild-type (C57BL/6J) and Vip-Cre mice. (A)** Fluorescent images of Th and Vip mRNAs in the DRN in C57BL/6J mice with close-ups of the DRN associated to a pie chart showing co-localization quantification. (B) Fluorescent images of Th and Vip mRNAs in the DRN in Vip-Cre mice on the top associated to a pie chart showing co-localization quantification. Below, fluorescent images of Cre recombinase and Vip mRNAs in the DRN in Vip-Cre mice associated to a pie chart showing co-localization quantification. Th: tyrosine hydroxylase; Vip: vasoactive intestinal peptide; Cre: Cre recombinase; aq: aqueduct of Sylvius.

**
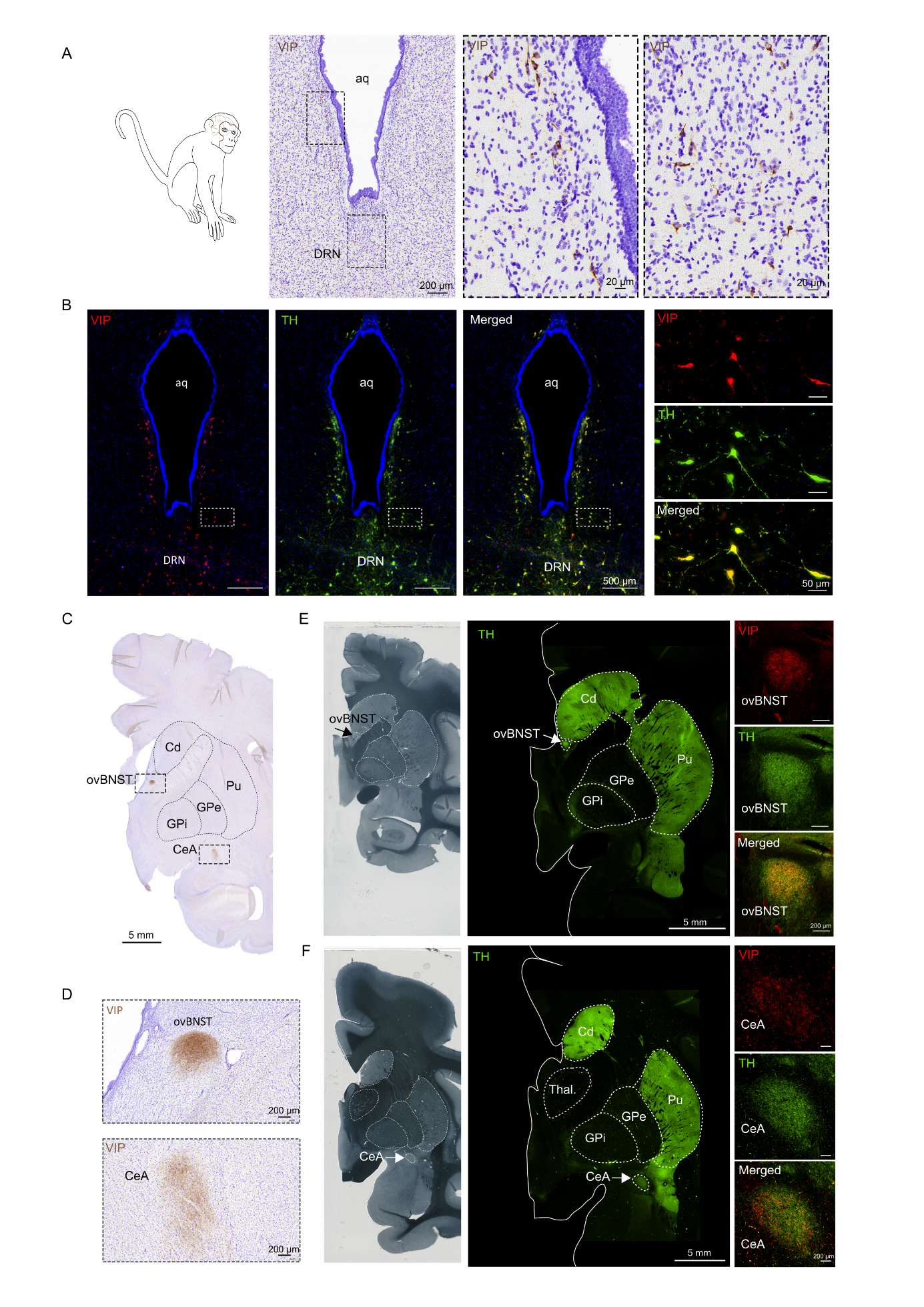
**

**Supplementary figure 2. Characterization of DRN_VIP_ neurons and terminals in the non-human primate (NHP). (A)** Schematics of a NHP and colorimetric immunostaining of VIP in the DRN (in brown) with a Nissl staining (purple). **(B)** Double immunofluorescence of VIP and TH in the DRN in the NHP with closeup images showing colocalization of VIP and TH. **(C)** Colorimetric immunostaining of VIP fibers in the ovBNST and CeA in the NHP. **(D)** Close-up images of VIP fibers in the ovBNST (on the top) and CeA (on the bottom) from C. **(E)** On the left, overview brightfield image of one hemisphere at the level of the ovBNST with a Black Sudan staining. On the right, same image with TH fluorescent immunostaining associated to close-up fluorescent images of the ovBNST with TH and VIP immunostaining. **(F)** On the left, overview brightfield image of one hemisphere at the level of the CeA with a Black Sudan staining. On the right, same image with TH fluorescent immunostaining associated to close-up fluorescent images of the CeA with TH and VIP immunostaining. Abbreviations: VIP: vasoactive intestinal peptide; TH: tyrosine hydroxylase; aq: aqueduct of Sylvius; DRN: dorsal raphe nucleus; ovBNST: oval nucleus of the bed nucleus of the stria terminalis; Cd: caudate putamen; Pu: putamen; GPe: external globus pallidus; GPi; internal globus pallidus; CeA: central nucleus of the amygdala; Thal: thalamus.


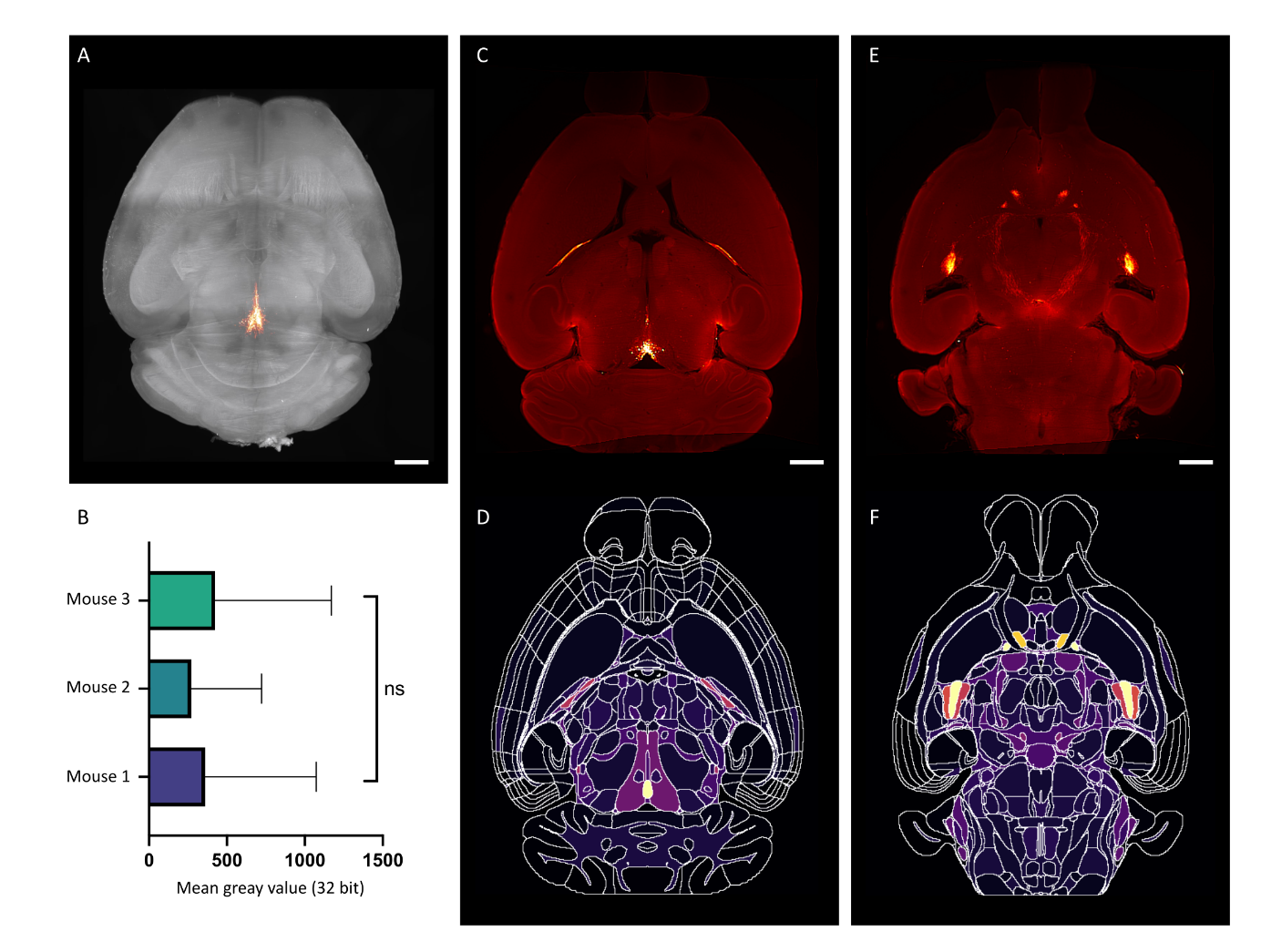


**Supplementary figure 3. Brain clearing and quantification approach of DRNVIP projections.** **(A)** Whole brain acquisition of autofluorescence (in grey) and DRN VIP cell bodies (in red). **(B)** Comparison of intensity level in DRN injection sites (Brown-Forsythe test, p-value = 0.9579). **(C, E)** Mean signal intensity of the three brains at DRN/ST terminalis **(C)** and CeA/BNST level **(E)**. **(D, F)** Mean heatmaps of labeling with atlas annotations. Scale bars: 1000 µm.

**
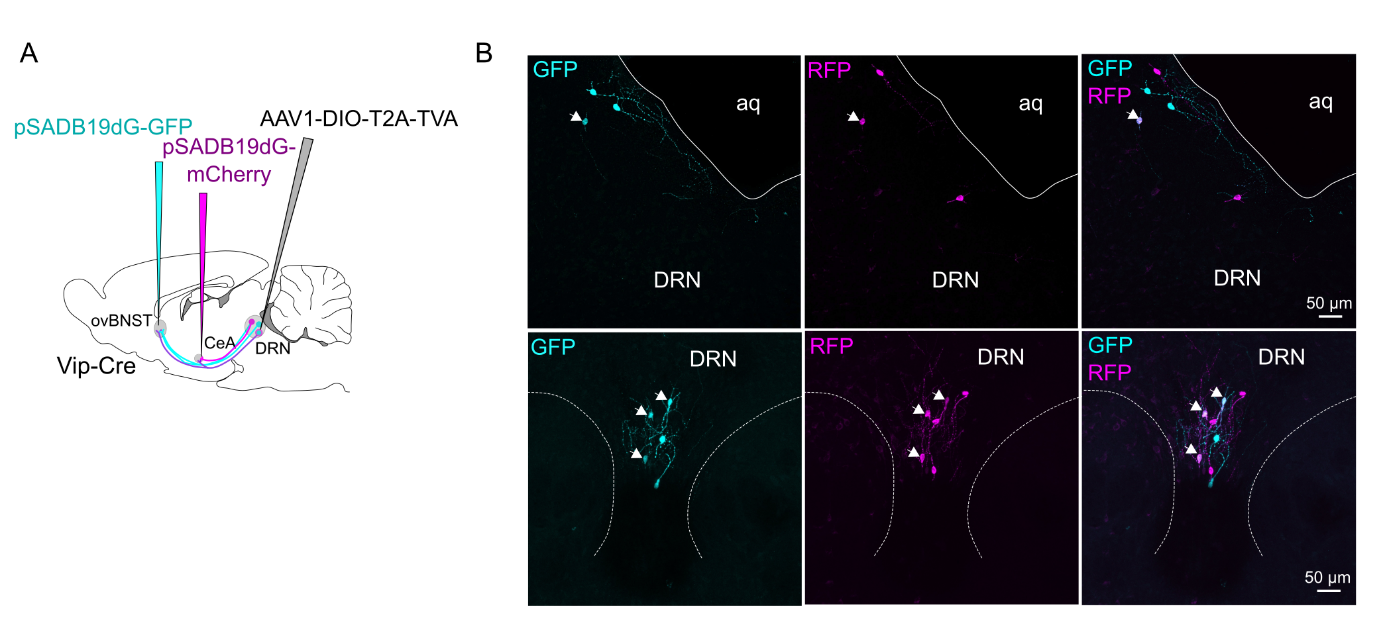
**

**Supplementary figure 4. Mapping of DRN_VIP_ collaterals to ovBNST and CeA. (A)** Schematic representation of the viral strategy used to demonstrate DRN_VIP_ collaterals to ovBNST and CeA. For this experiment a modified helper virus lacking the glycoprotein (AAV1-EF1a-DIO-TVA950-T2A-WPRE) was used associated with pseudorabies viruses conjugated to two different fluorescent reporters. **(B)** Confocal images of DRN neurons expressing either one or two of the pseudorabies reporters. On the top are neurons located near the aqueduct of Sylvius and below are DRN neurons located more ventrally.


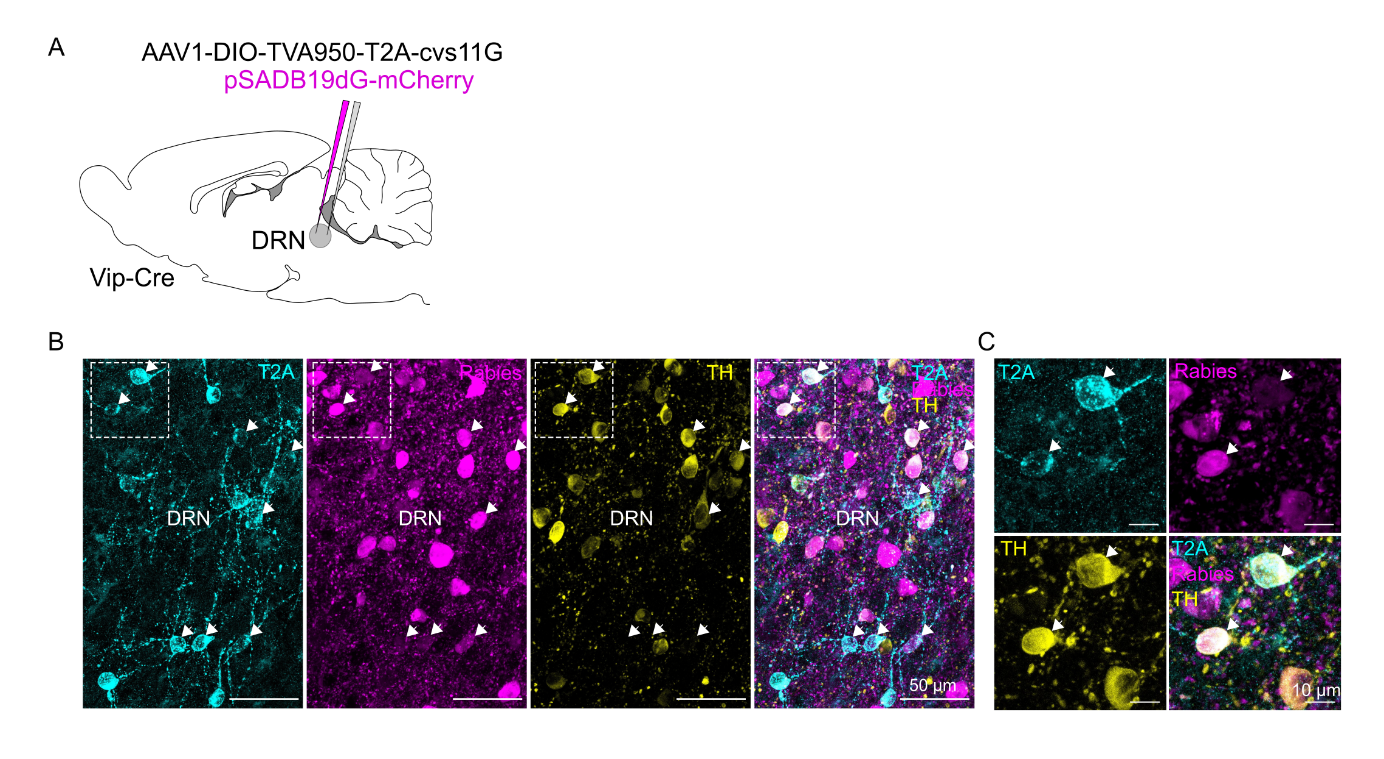


**Supplementary figure 5. Validation of the rabies strategy used for tracing inputs to DRN_VIP_ neurons. (A)** Schematic representation of the helper virus (AAV1-DIO-TVA950-T2A-csv11G) and pseudorabies virus (pSADB19dG-mCherry) injections in the DRN in Vip-Cre mice. **(B)** Confocal images of starter cells with colocalization of T2A, RFP and TH in helper cells.


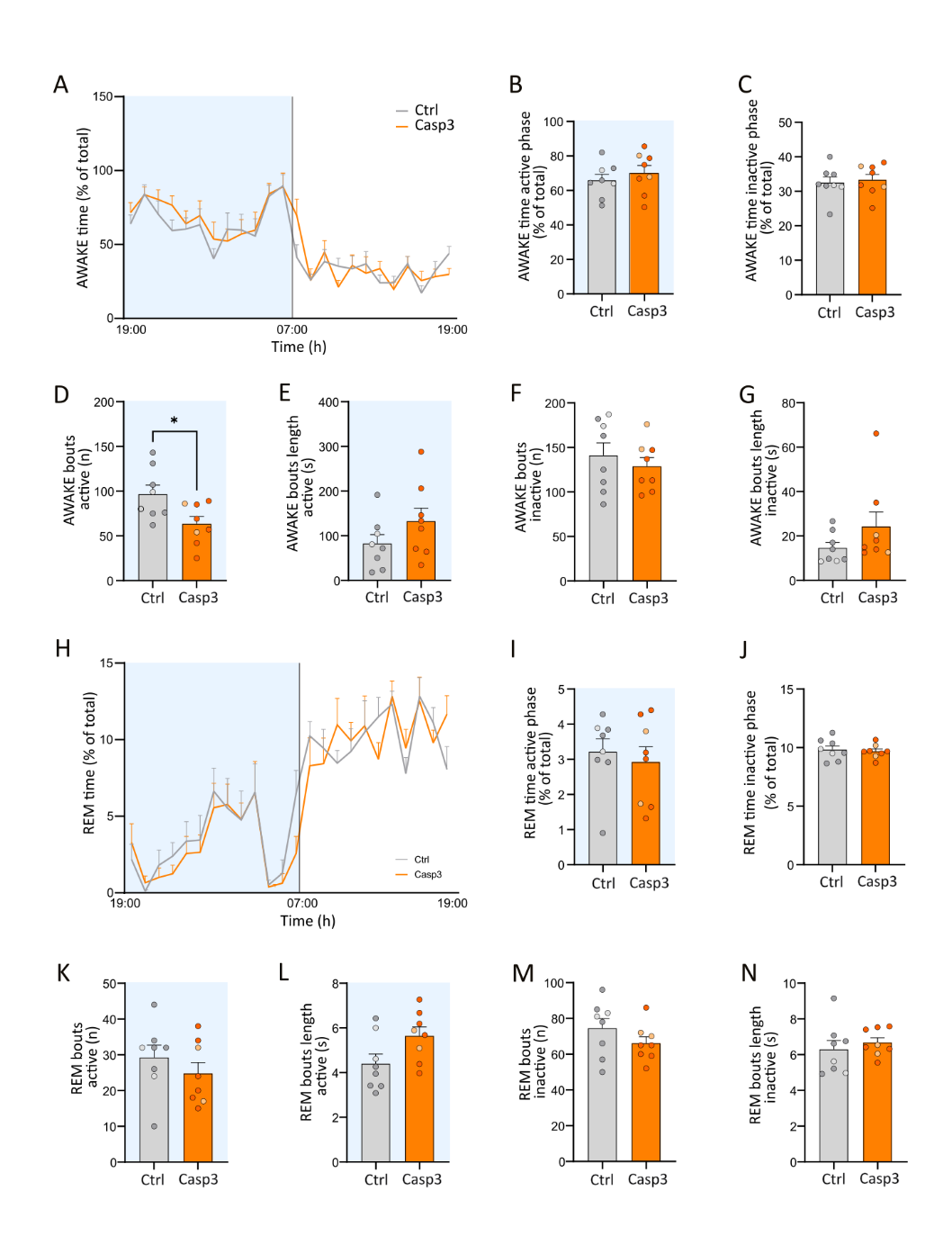


**Supplementary Figure 6. Effect of DRN_VIP_ genetic ablation on AWAKE and REM states in Vip-Cre mice. (A)** Graphics showing AWAKE percent time during the active (19- to 7 h) and the inactive (7- to 19 h) phases in Ctrl and Casp3 groups. **(B, C)** Bar graphs showing the AWAKE percent time during the active (19- to 7 h), (B, Unpaired T-test p-value=0.4488) and the inactive (7- to 19 h), (C, Unpaired T-test p-value=0.7368) phases in Ctrl and Casp3 groups. **(D, E)** Bar graphs showing the number (D, Unpaired T-test p-value=0.0232) and the length (E, Unpaired T-test p-value=0.1809) of AWAKE bouts during the active phase (19- to 7 h) in Ctrl and Casp3 groups. **(F, G)** Bar graphs showing the number (F, Mann-Whitney p-value=0.4889) and the length (G, Unpaired T-test p-value=0.1949) of AWAKE bouts during the inactive phase (7- to 19 h) in Ctrl and Casp3 groups. **(H)** Graphics showing REM percent time during the active (19- to 7 h) and the inactive (7- to 19 h) phases in Ctrl and Casp3 groups. **(I, J)** Bar graphs showing the REM sleep percent time during the active (19- to 7 h), (I, Unpaired T-test p-value=0.6160) and the inactive (7- to 19 h), (J, Unpaired T-test p-value=0.6850) phases in Ctrl and Casp3 groups. **(K, L)** Bar graphs showing the number (K, Unpaired T-test p-value=0.3492) and the length (L, Unpaired T-test p-value=0.0521) of REM sleep bouts during the active phase (19- to 7 h) in Ctrl and Casp3 groups. **(M, N)** Bar graphs showing the number (M, Unpaired T-test p-value=0.2228) and the length (N, Unpaired T-test p-value=0.5133) of REM sleep bouts during the inactive phase (7- to 19 h) in Ctrl and Casp3 groups. Light circles in graphs correspond to female mice while dark circles correspond to male mice. *p-value < 0.5.


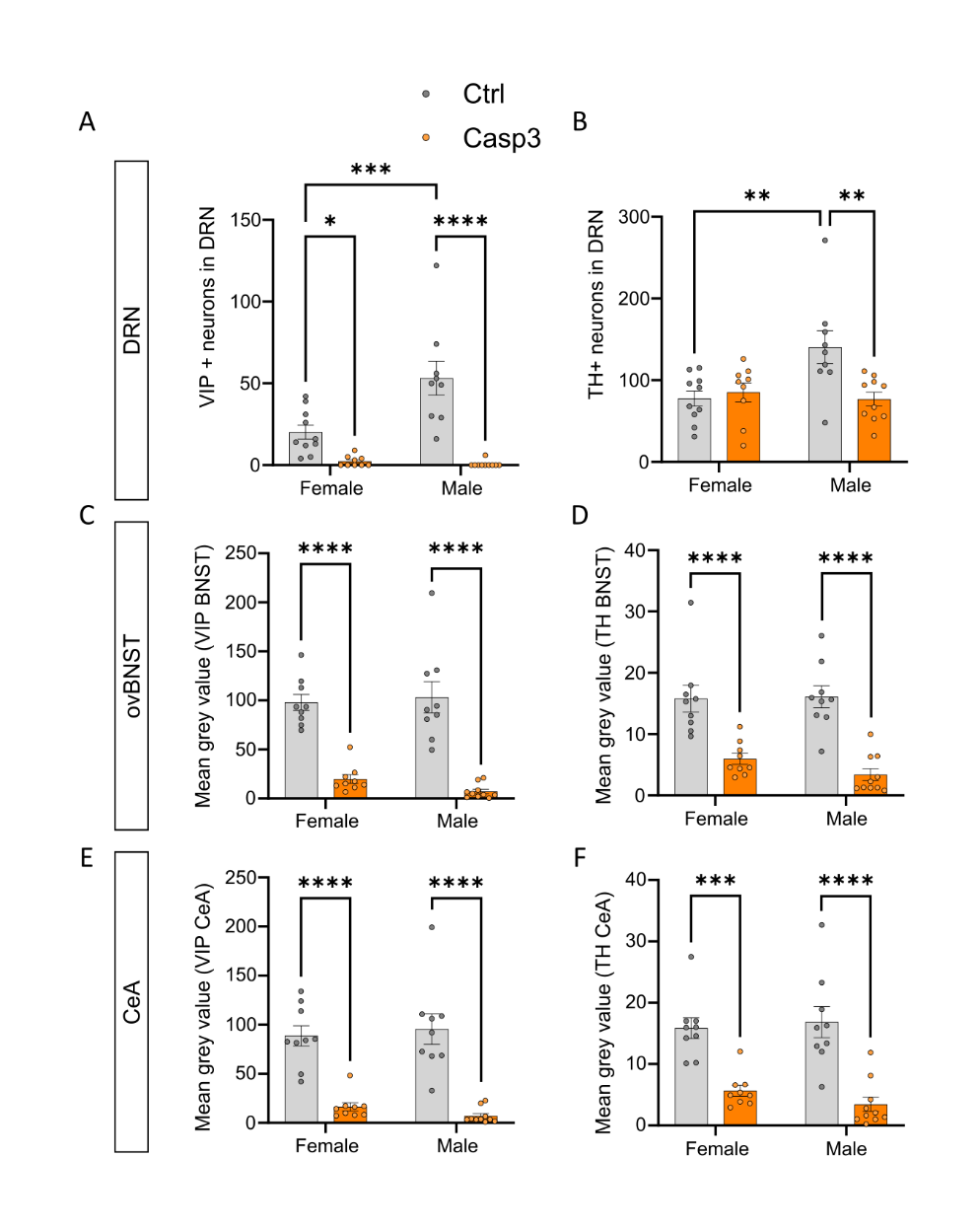


**Supplementary figure 7. Histological analysis of Casp3 according to sex. (A-B)** Graphs showing VIP and TH neurons quantification in the DRN in female and male mice. (C-D) Graphs showing mean grey value quantification of VIP and TH fibers in the ovBNST of female and male mice. (E-F) Graphs showing mean grey value quantification of VIP and TH fibers in the CeA of female and male mice. Graphs show mean ± SEM. *p-value < 0.5; **p-value < 0.01; ***p-value < 0.001; ****p-value < 0.0001.


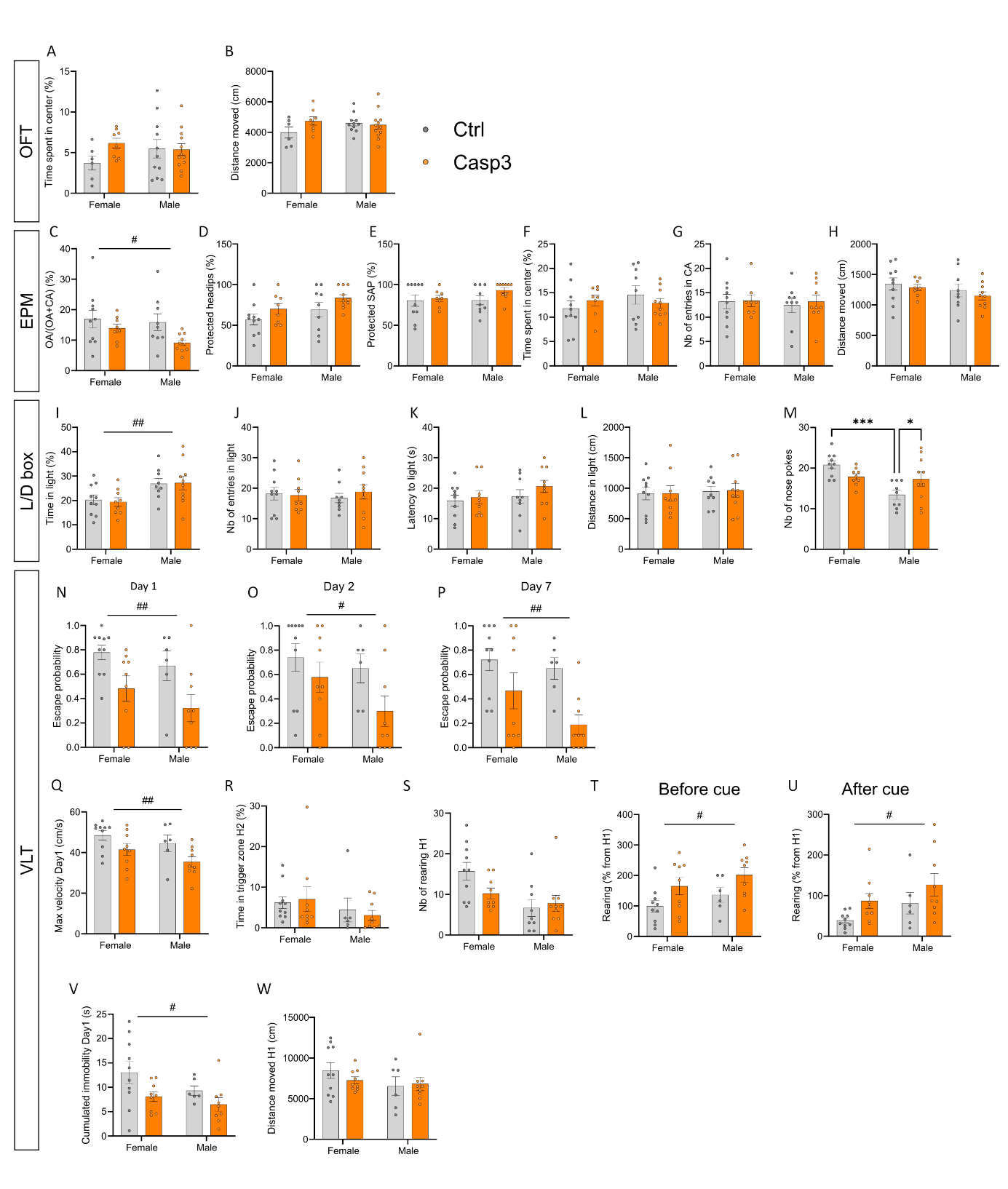


**Supplementary figure 8. Effect of DRN_VIP_ genetic ablation on risk assessment, anxiety and locomotion in Vip-Cre mice according to sex. (A)** In the OFT, quantification of the time in spent in center (2Way ANOVA, no effect) and **(B)** distance moved (2Way ANOVA, no effect) between control and Casp3 female and male mice. **(C)** In the EPM: quantification of the percentage of time spent in OA expressed in ratio (2Way ANOVA, Group effect #p-value = 0.0362), **(D)** protected headips (2Way ANOVA, no effect), **(E)** protected SAP (2Way ANOVA, no effect), **(F)** percentage of time spent in center (2Way ANOVA, no effect), **(G)** number of entries in CA (2Way ANOVA, no effect) and **(H)** distance moved (2Way ANOVA, no effect) between control and Casp3 female and male mice. **(I)** In the L/D box, quantification of the percentage of time spent in the light compartment (2Way ANOVA, Sex effect ##p-value = 0.0029), **(J)** number of entries in light compartment (2Way ANOVA, no effect), **(K)** latency to light compartment (2Way ANOVA, no effect), **(L)** distance moved in light compartment (2Way ANOVA, no effect) and **(M)** number of nose pokes (2Way ANOVA, Interaction p-value = 0.0085, Uncorrected Fisher’s test *p-value = 0.0310 and ***p-value = 0.0001). **(N, O and P)** In the VLT, quantification of the escape probability (2Way ANOVA, Group effect: ##p-value = 0.0031 at Day1, #p-value = 0.0493 at Day2, ##p-value = 0.0029 at Day7), **(Q)** the mean velocity at Day1 (2Way ANOVA, Group effect: ##p-value = 0.0093), (R) the percentage in the trigger zone in the habituation of Day2 (H2) (2Way ANOVA, no effect), (S) the number of rearing zone in the habituation of Day1 (H1) (2Way ANOVA, no effect), (T and U) the number of rearing 30 s before and after the onset of the visual cue in percentage of H1 (2Way ANOVA, Group effect: before cue #p-value = 0.0112 , after cue #p-value = 0.0324), (V) cumulated immobility at Day1 (2Way ANOVA, Group effect #p-value = 0.0272), (W) distance moved (2Way ANOVA, no effect).


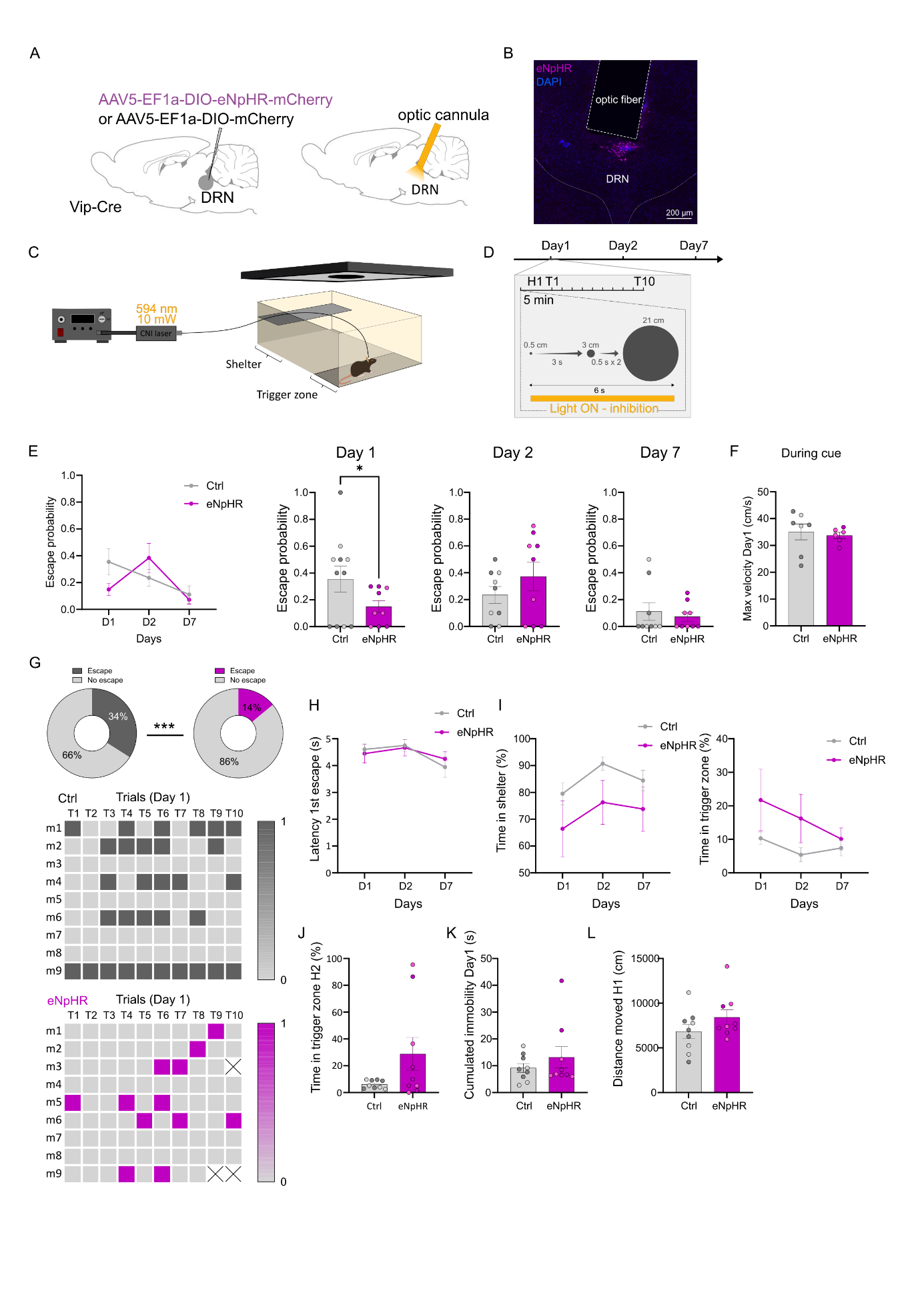


**Supplementary figure 9. Effect of optogenetic inhibition of DRN_VIP_ neurons on defensive behaviors. (A)** Schematic representation of AAV5-EF1a-DIO-eNpHR-mCherry virus or control virus injections and optic fiber placement in the DRN in Vip-Cre mice. **(B)** Epifluorescent image of the optic fiber track and mCherry neurons in the DRN. **(C)** Schematic representation of the visual looming test associated to optogenetic inhibition and **(D)** description of the visual cue protocol synchronized to the laser activation protocol. **(E)** Graphs showing first escape probability of eNpHR mice and controls along days and escape probability for each day (Unpaired T-test one-tailed, *p-value = 0.0463). **(F)** Graph showing the mean max velocity for eNpHR and control mice when the velocity threshold for “escape” trials was set on 20 cm/s to measure the vigor of the return to the shelter. **(G)** Graph showing escape probability along days (Pie chart graphs and heatmaps showing the number of trials counted as an escape or not in eNpHR mice and controls (Fisher's exact test one-sided, ***p-value < 0.0007). **(H)** Graph showing the latency to escape at the first escape along days in eNpHR and control mice. **(I)** Graphs showing the percentage of total time spent in shelter and trigger zone along days in eNpHR and control mice. **(J)** Graph showing the percentage of time spent in the trigger zone during the habituation period of Day 2. **(K)** Graph showing the cumulated immobility during visual cues and **(L)** Graph showing the distance traveled during habituation at Day 1. Light circles in graphs correspond to female mice while dark circles correspond to male mice. *p-value < 0.05, ***p-value < 0.001. Graphs show mean ± SEM.


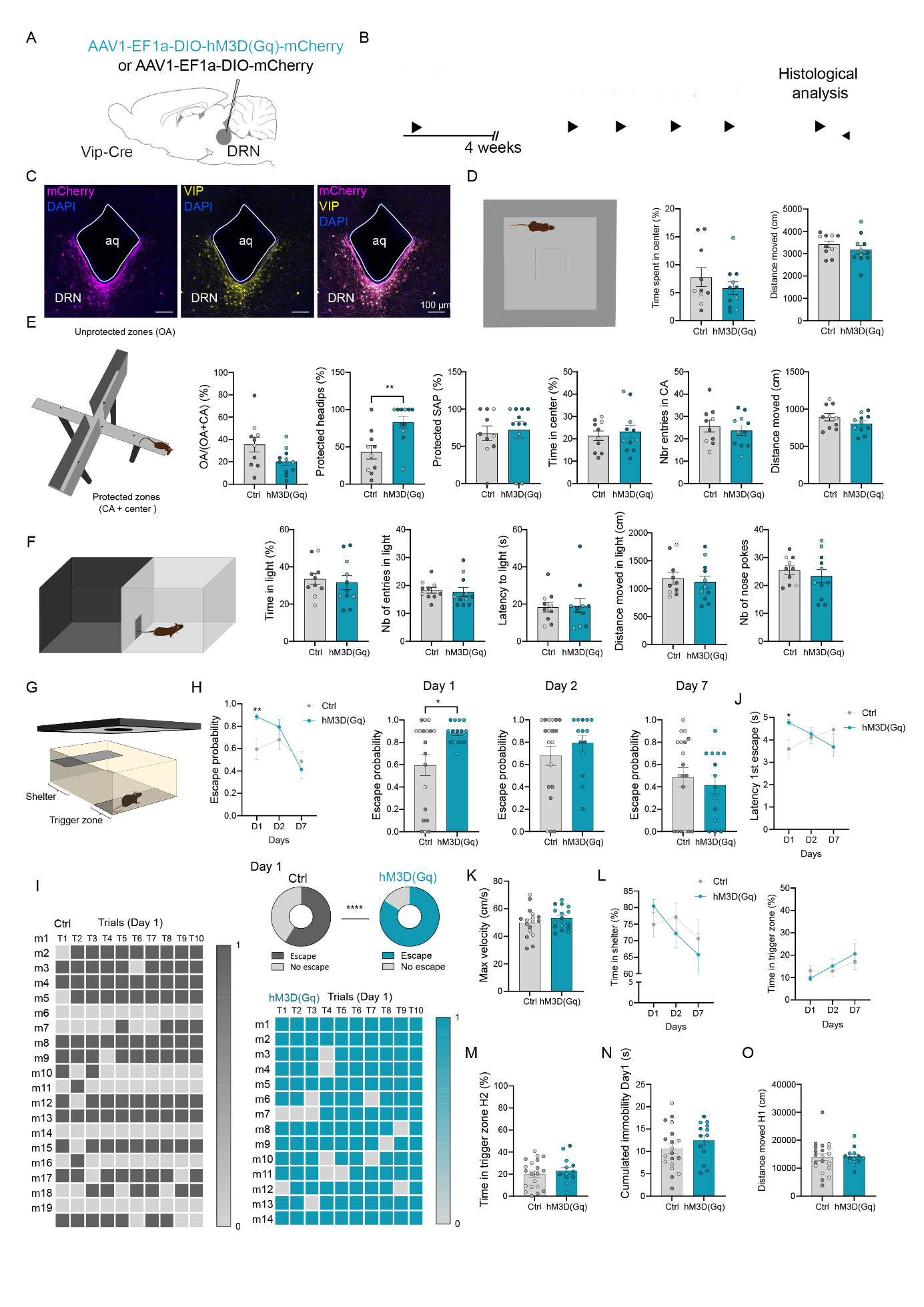


**Supplementary figure 10. Effect of chemogenetic activation of DRN_VIP_ neurons on risk assessment, anxiety, locomotion and defensive behaviors in Vip-Cre mice. (A)** Schematic representation of AAV1-EF1a-DIO-hM3D(Gq)-mCherry virus or control virus injections in the DRN in Vip-Cre mice. **(B)** Chronological timeline of the experimental procedure from virus injection to the four behavioral tests depicted below. **(C)** Confocal images showing colocalization of the mCherry fluorescent reporter in DRN_VIP_ neurons. **(D)** Schematic representation of the Open Field Test (OFT) and graphs showing the percentage of time spent in the center of the arena (Mann-Whitney, p-value = 0.5573) and the distance moved (Unpaired t-test, p-value = 0.3253). **(E)** Schematic representation of the Elevated Plus Maze (EPM) arena with arrows indicating protected (closed arms CA and center) and unprotected (open arms OA) zones. Quantification of the time spent in OA expressed in ratio (Unpaired T-test, p-value = 0.0513), protected headips (Mann-Whitney test, **p-value=0.0034), protected SAP (Mann-Whitney test, p-value=0.4696), time spent in center (Unpaired T-test, p-value=0.6294), number of entries in CA (Unpaired T-test, p-value = 0.5662) and distance moved (Unpaired T-test, p-value = 0.1753) between control and hM3D(Gq) mice. **(F)** Schematic representation of the Light/Dark box (L/D box) arena. Quantification of the percentage of time spent in the light compartment (Unpaired T-test, p-value = 0.6877), number of entries in light compartment (Mann-Whitney test, p-value=0.3016), latency to light compartment (Mann-Whitney test, p-value = 0.6398), distance moved in light compartment (Unpaired T-test, p-value = 0.6689) and number of nose pokes (Unpaired T-test, p-value=0.4775). **(G)** Schematic representation of the Visual Looming Test (VLT). **(H****)** Graphs showing escape probability along days (Mixed-effects analysis, **p-value = 0.0059) and escape probability for each day (Day 1: Mann-Whitney, One -tailed, *p-value = 0.0355). **(I)** Pie chart graphs and heatmaps showing the number of trials counted as an escape or not in hM3D(Gq) mice and controls (Fisher's exact test one-sided, ****p-value < 0.0001). **(J)**  Graph showing the latency of the first escape along days (Mixed-effects analysis, Day 1: *p-value = 0.016). **(K)** Graph showing the max velocity during escapes at Day 1 (Unpaired T-test one-tailed, p-value > 0.1662). (L) Graphs showing the percentage of total time spent in the shelter and in the trigger zone at Day 1. (M) Graph showing the percentage of time spent in the trigger zone during the habituation period of Day 2 (Unpaired t-test, p = 0.4847). (N) Graph showing the cumulated immobility time during the 7 s after the onset of the visual cue at Day 1 (Unpaired t-test one-tailed, p = 0.1376). (O) Graph showing the traveled distance in the VLT arena during the habituation period of Day 1 (Unpaired T-test, 0.9155). Light circles in graphs correspond to female mice while dark circles correspond to male mice. *p-value < 0.5, **p-value < 0.01, ****p-value < 0.0001. Graphs show mean ± SEM.


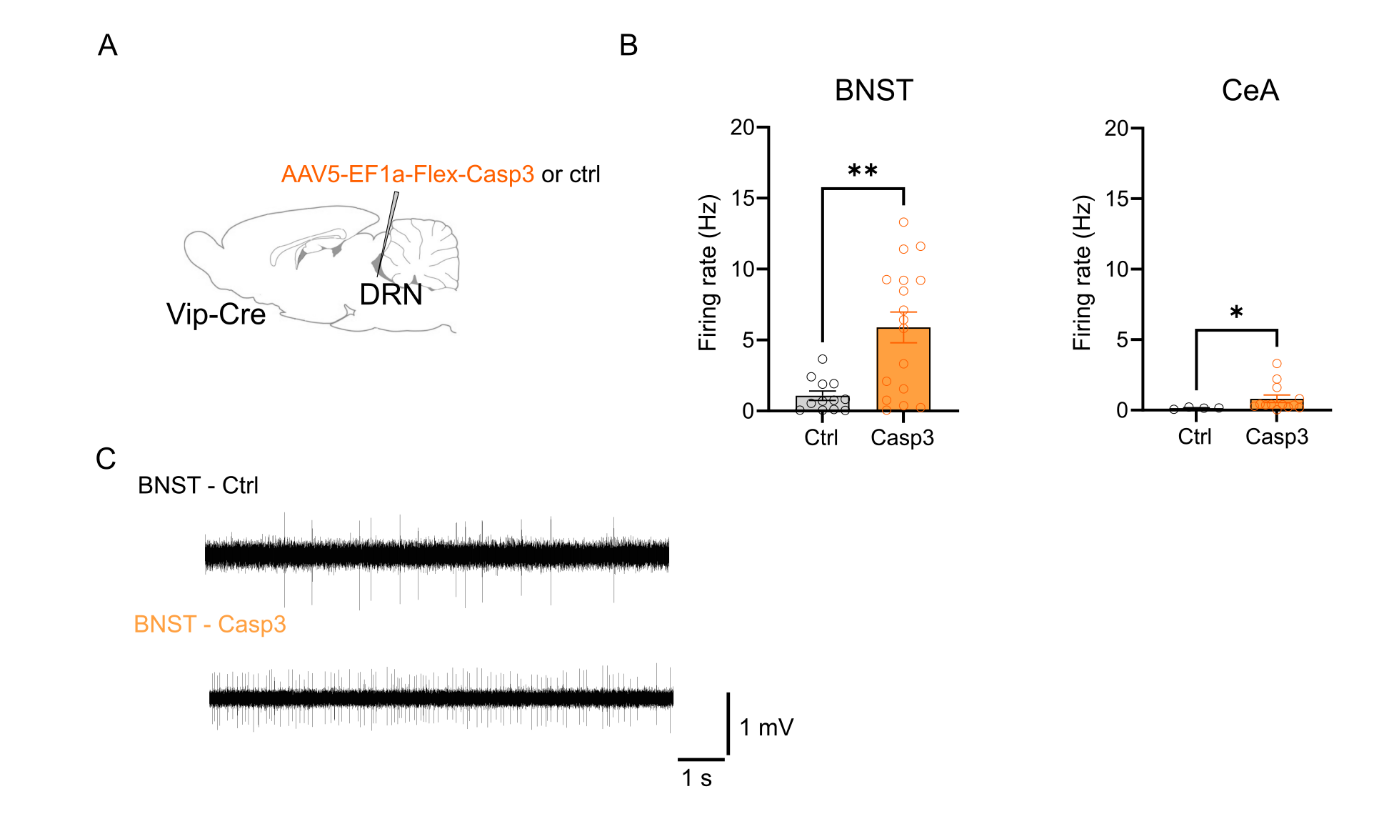


**Supplementary figure 11. Impact of DRN_VIP_ neurons genetic ablation on i*n vivo* electrophysiological properties of BNST and CeA neurons. (A)** Schematic representation of AAV5-EF1a-Flex-Casp3 or control virus injections in the DRN in Vip-Cre mice. **(B)** Graphs showing the firing rate of BNST and CeA neurons in Casp3 and control mice (BNST, Mann-Whitney p-value=0.0045); CeA, Mann-Whitney p-value=0.0227). **(C)** Representative single-cell extracellular recording traces of BNST neurons in control and Casp3 mice.
